# Dietary Patterns and Cardiovascular Diseases in Individuals with Type 2 Diabetes: A Systematic Review and Meta-Analysis of Prospective Observational Studies

**DOI:** 10.1016/j.advnut.2026.100640

**Published:** 2026-04-16

**Authors:** Janett Barbaresko, Lisa Kannenberg, Edyta Schaefer, Alexander Lang, Lukas Schwingshackl, Manuela Neuenschwander, Sabrina Schlesinger

**Affiliations:** 1 Institute for Biometrics and Epidemiology, German Diabetes Center, Leibniz Center for Diabetes Research at Heinrich Heine University Düsseldorf, Düsseldorf, Germany; 2 German Center for Diabetes Research (DZD), Partner Düsseldorf, Munich-Neuherberg, Germany; 3 Institute for Evidence in Medicine, Faculty of Medicine and Medical Center, University of Freiburg, Freiburg, Germany

**Keywords:** dietary patterns, cardiovascular diseases, type 2 diabetes, systematic review, meta-analysis

## Abstract

Individuals with type 2 diabetes are at increased risk of cardiovascular diseases (CVD). Dietary behavior plays an important role in both the management of diabetes and the prevention of CVD. We aimed to summarize the current evidence on associations between dietary patterns and the risk of CVD outcomes in individuals with type 2 diabetes. PubMed, Embase, and Cochrane Library were systematically searched for prospective observational studies investigating dietary patterns in association with CVD outcomes in individuals with diabetes. Summary risk ratios (SRR) with 95% confidence intervals (CIs) were calculated using random effects model. The certainty of evidence was evaluated using the Grading of Recommendations, Assessment, Development, and Evaluation approach. In total, we identified 57 studies. Moderate certainty of evidence was found for the association between the Mediterranean diet and CVD (SRR per 1 point: 0.95; 95% CI: 0.93, 0.97; *n* = 6). Other plant-based diets such as the Dietary Approaches to Stop Hypertension (per 5 points: 0.96; 95% CI: 0.89, 1.03; *n* = 3), EAT-Lancet diet (per 3 points: 0.65; 95% CI: 0.45, 0.95, *n* = 3) or country-specific dietary guidelines (high compared with low: 0.80; 95% CI: 0.76, 0.84; *n* = 9) were associated with a lower incidence of CVD outcomes (low or very low certainty of evidence). Higher adherence to the unhealthy plant-based index and dietary inflammatory index was positively associated with CVD (low and very low certainty of evidence). No clear associations were found for overall and vegetable-based plant-based index, low-carbohydrate diets, glycemic load, glycemic index, and CVD in persons with type 2 diabetes. Most studies were rated as high risk of bias due to confounding. In conclusion, the results suggest a possible beneficial association between plant-based dietary patterns and CVD outcomes in individuals with type 2 diabetes. However, the certainty of evidence was low to very low, highlighting the need for further well-designed prospective studies.

This trial was registered at PROSPERO as CRD42018110669.

**Statements of significance**

For the first time, the results of the present systematic review and meta-analysis suggest a possible beneficial role of several plant-based diets in association with CVD exclusively in persons with type 2 diabetes. However, confidence in these findings is low, underscoring the need for further high-quality research in this area.

## Introduction

It is well-known that the incidence and prevalence of diabetes are still rising. For 2024, the number of adults living worldwide with diabetes has been estimated to be ∼589 million [1]. Persistent hyperglycemia is associated with several complications and comorbidities, foremost cardiovascular diseases (CVD). Recent evidence indicated that compared with persons without diabetes, individuals with diabetes have a 60% higher relative risk of developing any type of CVD [1]. Subsequently, the presence of diabetes and its complications is associated with higher burden of diseases and premature death [2]. This indicates that the prevention of CVD in persons with type 2 diabetes is of urgent public health interest.

Diet plays an important role in both diabetes management and prevention of CVD. There are dietary guidelines for the prevention and management of diabetes-related complications available, such as the recently published recommendations by the American Diabetes Association [3] or Diabetes and Nutrition Study Group of the European Association for the Study of Diabetes [4]. Key aspects of these recommendations include the higher consumption of minimally processed plant foods, namely fruit and vegetables, whole grains, legumes, nuts, and seeds; and lower consumption of processed or ultraprocessed foods containing red and processed meat, refined grains, sugar, and sodium. However, these guidelines are based on findings from the general population [3,4]. Additionally, a recent comprehensive umbrella review on diet and diabetes management summarizing meta-analyses of clinical trials found strong evidence for beneficial effects of a low-carbohydrate diet on glycated hemoglobin **(**HbA1c) and triglyceride concentrations [5]. Moreover, plant-based diets showed clinically important reductions of anthropometric measures [5]. However, these findings are based on surrogate markers, and evidence on dietary behavior and clinically relevant outcomes such as CVD in persons with type 2 diabetes is lacking.

Thus, the aim of the present study was to systematically summarize the current evidence on associations between dietary patterns and the risk of CVD incidence or mortality in individuals with type 2 diabetes.

## Methods

The present systematic review and meta-analysis were planned, conducted, and reported according to the PRISMA 2020 statement [6] and registered in PROSPERO (registration number: CRD42018110669). We published a detailed protocol of the whole project on diet and diabetes-related outcomes in individuals with type 2 diabetes [7], and this report focuses on CVD outcomes. We deviate from the published protocol [7] by searching Embase and the Cochrane Library instead of Web of Science to identify further relevant studies. Moreover, we revised the search terms for this specific search for CVD, and we used the risk of bias in nonrandomized studies–of exposure (ROBINS-E) tool instead of the risk of bias in nonrandomized studies–of intervention tool, as the ROBINS-E tool has been published in the meantime and is more suitable for our study context.

### Eligibility criteria

The detailed inclusion and exclusion criteria are presented in Supplemental Table 1. We included studies if the following criteria were met: *1*) population: participants with type 2 diabetes aged ≥18 y; *2*) exposure: any dietary pattern, including a priori dietary patterns such as dietary indices [e.g., Healthy Eating Index (HEI) or plant-based dietary index (PDI)] and dietary scores (e.g., Mediterranean diet score, low-carbohydrate diet score) as well as exploratory dietary patterns (e.g., derived by principal component analysis); *3*) outcomes: CVD incidence and mortality, including coronary artery diseases, heart failure and cerebrovascular diseases (International Classification of Diseases (ICD) codes as defined by studies); *4*) study design: prospective observational studies (including prospective cohort studies, nested case-control and case-cohort studies) published in a peer-reviewed journal. We excluded studies which *1*) investigated mixed populations (participants without diabetes included) or solely investigated children, adolescents, participants with prediabetes, type 1 diabetes or gestational diabetes; *2*) reported dietary patterns only in combination with other lifestyle factors (e.g., lifestyle index composed of diet, physical activity, and smoking); *3*) focused solely on single foods/food groups (e.g., only fruit and vegetables); or *4*) were of cross-sectional or case-control design, conference abstracts, comments, and letters or reviews.

### Search strategy and data extraction

The systematic literature search was conducted in PubMed, Embase, and Cochrane Library on 10 January, 2025 by using predefined search terms (Supplemental Table 2). Thereafter, an e-mail alert from PubMed was followed to identify new relevant studies until 24 October, 2025. The study selection process was conducted independently by 2 researchers from a group of 4 (JB, ES, AL, and MN). Any disagreements were resolved by consensus or by consultation of a third researcher (SS). In addition, we conducted forward and backward citation searching using the Scopus database to identify further relevant studies. In case of multiple publications of the same exposure and outcome association in the same study population, we included the most comprehensive report, including the largest sample size and/or cases and longest duration of follow-up.

Relevant data were extracted by 1 researcher and checked by a second researcher for accuracy. Following data were extracted: first author’s last name, year of publication, cohort name and country, sample size, number of CVD cases, sex, and age of the participants at study entry, duration of follow-up, type of exposure and assessment method, quantity of dietary patterns as well as cases and person-years across categories, type of CVD and assessment, the fully-adjusted risk ratio (hazard or odds ratios) with corresponding 95% confidence interval (CI), and adjustment factors. We contacted authors for missing data and received additional data from 6 studies [[8], [9], [10], [11], [12], [13]].

### Risk of bias and certainty of evidence assessment

The risk of bias of each included study was assessed independently by 2 researchers (JB and LK) using the ROBINS-E tool [14]. A detailed description of the 7 domains (confounding, exposure measurement, selection of participants, post exposure interventions, missing data, outcome measurement, and selection of reported results) is presented in Supplemental Table 3. Based on the literature, we identified the following potentially relevant confounders: age, sex, socioeconomic status (e.g., education, income, or Townsend deprivation index), smoking, physical activity, total energy intake, and a measure for diabetes severity (diabetes duration or diabetes medication) (Supplemental Table 3). If a study was judged as being at high risk of bias in the first domain (due to confounding), the study was overall rated as high risk of bias, and no further assessment was performed. This triage approach has already been successfully used [15]. Any discrepancies were discussed to reach a consensus or by consultation with a third researcher (SS).

The certainty of evidence for each association was evaluated independently by 2 researchers (JB and LS) using the Grading of Recommendations, Assessment, Development, and Evaluation approach [16]. Using ROBINS-E for risk of bias assessment, the certainty of evidence starts with an initial high rating. The Grading of Recommendations, Assessment, Development, and Evaluation approach considers the risk of bias of included studies, inconsistency, indirectness, imprecision between the studies, publication bias, magnitude of the effect, and dose–response relationship.

### Statistical analysis

Summary risk ratios (SRRs) and corresponding 95% CI for each dietary pattern in association with CVD outcomes were calculated by using a random effects model by DerSimonian and Laird [17]. In case of separate estimates for men and women, we combined the data using a fixed effect model before entering the study estimate into the overall meta-analysis. If sufficient data (quantified exposure value, risk ratios with 95% CIs, and the number of cases and person-years) were available, we conducted linear and nonlinear dose–response meta-analyses as described by Crippa et al. [18] For linear dose–response, the dose was selected as provided by studies and/or depending on the overall dietary score, e.g., per 1 point for the Mediterranean diet (range of 0‒9 points) and per 5 points for scores with larger ranges such as Dietary Approaches to Stop Hypertension (DASH, range 8‒40 points). If the number of cases in single categories was not provided in a study, information on the total number of cases and total person-years or the number of total participants plus follow-up period was used to distribute the number of cases across the quantiles accordingly [19]. If a range of points/units were presented for dietary patterns, we used the midpoint value as exposure level, and for open categories, the same width as the adjacent category was assumed. Tau^2^ (τ^2^) was calculated to assess the statistical heterogeneity between studies, and inconsistency was assessed by calculating *I*^2^ statistic. If >3 studies were available, we calculated prediction intervals (PIs) to indicate the variance of estimates across the studies [20].

Furthermore, we planned to conduct subgroup analyses to investigate sources of heterogeneity [stratified analysis by e.g., sex, age, study length, study origin, outcome (types of CVD such as myocardial infarction or stroke), duration of diabetes] and potential publication bias visually using funnel plots and statistically by Egger’s test if ≥10 studies on a dietary pattern were available.

All statistical data analyses were conducted using the statistical software Stata (version 14.2, StataCorp LLC). For dose–response meta-analyses, we used the new command drmeta provided by Orsini [21].

## Results

**FIGURE 1:**
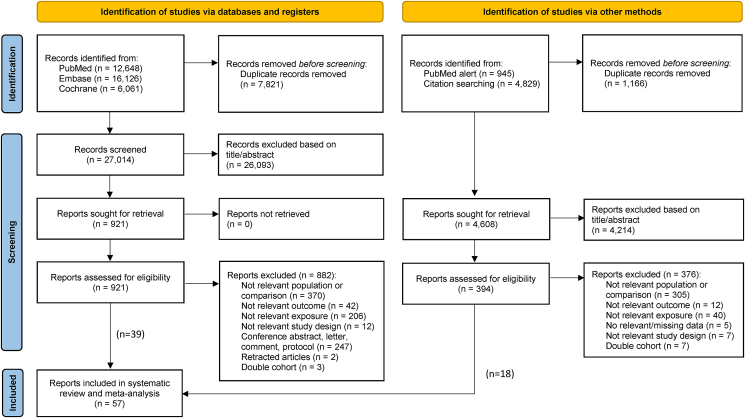
PRISMA flow chart illustrating the literature search process.

After removing duplicates, 27,014 titles and abstracts have been screened (Figure 1). Out of 921 full-text articles, we included 39 studies [9,10,13[9,10,13,[22], [23], [24], [25], [26], [27], [28], [29], [30], [31], [32], [33], [34], [35], [36], [37], [38], [39], [40], [41], [42], [43], [44], [45], [46], [47], [48], [49], [50], [51], [52], [53], [54], [55], [56], [57]. The list of included and excluded studies with exclusion reasons can be found in Supplemental Table 4. Eighteen additional studies could be identified by following the PubMed alert [11,[58], [59], [60], [61]] and citation searching [8,12,[62], [63], [64], [65], [66], [67], [68], [69], [70], [71], [72]]. Finally, we included a total of 57 studies in the present systematic review and meta-analysis.

The detailed characteristics of all included prospective studies are presented in Supplemental Table 5. Out of the 57 studies, 22 have been conducted in the United States, 21 in Europe, 11 in Asia, 1 in Australia, and 2 studies included different countries. Sample sizes ranged from 87 to 22,473 participants with type 2 diabetes. The follow-up duration ranged from 2.4 y to 30 y. Most studies assessed the diet using a food frequency questionnaire, followed by 24 h dietary recalls. We identified plant-based dietary patterns such as the EAT-Lancet planetary health diet, the PDI, Mediterranean diet, and DASH, dietary patterns measuring adherence to dietary guidelines (e.g., HEI), or nutrient-based dietary patterns such as low-carbohydrate diet score, dietary inflammatory index, glycemic index, or glycemic load. Most studies investigated total CVD outcomes, comprising nonfatal and fatal CVD events, as well as CVD incidence and CVD mortality separately. Some studies investigated coronary artery disease, stroke, heart failure, or peripheral artery disease (Supplemental Table 5).

An overview of the risk of bias of included studies is shown in Supplemental Table 6. Nine studies were judged as being at moderate and 48 studies were judged as being at serious risk of bias. The main reason for serious risk of bias was confounding, indicating that the majority of studies (82%) were not adjusted for the most relevant confounders (e.g., socioeconomic status, total energy intake, and/or diabetes duration/ severity).

### Plant-based dietary patterns

**FIGURE 2:**
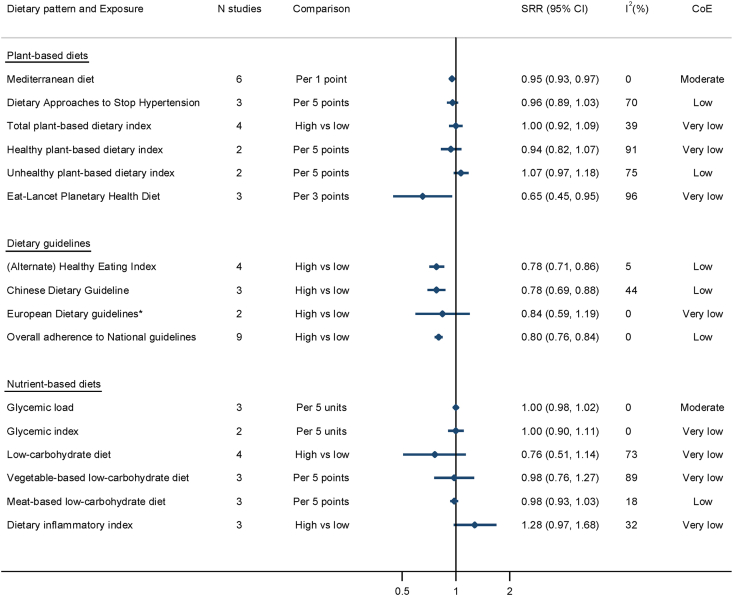
Results of meta-analyses on associations between dietary patterns and CVD outcomes in individuals with type 2 diabetes. CI, confidence interval; CoE, certainty of evidence; CVD, cardiovascular disease; SRR, summary risk ratio. ∗Including 1 Swedish and 1 Dutch study.

The results of all meta-analyses are shown in Table 1 and Figure 2. We found moderate certainty of evidence (Supplemental Table 7) for an inverse association between the Mediterranean diet and CVD outcomes (SRR per 1 point: 0.95; 95% CI: 0.93, 0.97; *n* = 6; τ^2^: <0.0001; PI: 0.92, 0.98; *I*^2^: 0%, Supplemental Figure 1 and Figure 2). A higher adherence to a Mediterranean diet was associated with a 31% lower CVD incidence/mortality (SRR high compared with low: 0.69; 95% CI: 0.58, 0.83; *n* = 8; τ^2^: 0.03; PI: 0.43, 1.13; *I*^2^: 62%), compared with a low adherence (Table 1). There was no indication of nonlinearity (Supplemental Figure 1). With regard to the DASH diet, a higher adherence was associated with lower CVD events (SRR high compared with low: 0.85; 95% CI: 0.73, 0.98; *n* = 6; τ^2^: 0.015; PI: 0.57, 1.26; *I*^2^: 51%), compared with a low adherence (Table 1 and Supplemental Figure 2). The linear dose–response meta-analysis showed a similar direction (SRR per 5 points: 0.96; 95% CI: 0.89, 1.03; *n* = 3; τ^2^: 0.003; I^2^: 70%), graded with low certainty of evidence (Supplemental Table 7). There was no indication of nonlinearity (Supplemental Figure 2). Higher adherence to the total PDI was not associated with CVD compared with lower adherence (Supplemental Figure 3, low certainty of evidence). A 5 points higher adherence to a healthy PDI was associated with a 6% lower incidence of CVD outcomes (SRR per 5 points: 0.94; 95% CI: 0.82, 1.07; *n* = 2; τ^2^: 0.009; *I*^2^: 91%, Supplemental Figure 4), whereas higher adherence to an unhealthy PDI was positively associated with CVD (SRR per 5 points: 1.07; 95% CI: 0.97, 1.18; *n* = 2; τ^2^: 0.004; *I*^2^: 75%, Supplemental Figure 5); however, both associations were imprecisely estimated and graded with very low certainty of evidence (Supplemental Table 7). Nonlinearity could not be tested due to missing data.

**TABLE 1:** Overview of all associations between dietary patterns and cardiovascular diseases in individuals with type 2 diabetes

Exposure	High vs. low		Linear dose–response			Indication for nonlinearity	Certainty of evidence
	*n*	SRR (95% CI)	*n*	Unit	SRR (95% CI)		
Plant-based dietary patterns							
Mediterranean diet	8	0.69 (0.58, 0.83)	6	1 point	0.95 (0.93, 0.97)	No	Moderate
DASH	6	0.85 (0.73, 0.98)	3	5 points	0.96 (0.89, 1.03)	No	Low
PDI	4	1.00 (0.92, 1.09)	—			—	Very low
Healthy PDI	5	0.90 (0.78, 1.04)	2	5 points	0.94 (0.82, 1.07)	—	Very low
Unhealthy PDI	4	1.13 (0.96, 1.33)	2	5 points	1.07 (0.97, 1.18)	—	Low
EAT-Lancet planetary health diet	—		3	3 points	0.65 (0.45, 0.95)	—	Very low
Dietary guidelines							
(Alternate) HEI	4	0.78 (0.71, 0.86)	—			—	Low
Chinese dietary guideline	3	0.78 (0.69, 0.87)	—			—	Low
European dietary guideline^1^	2	0.84 (0.59, 1.19)	—			—	Very low
Overall national dietary guidelines	9	0.80 (0.76, 0.84)	—			—	Low
Nutrient-based dietary patterns							
Glycemic load	—		3	5 units	1.00 (0.98, 1.02)	—	Moderate
Glycemic index	—		2	5 units	1.00 (0.90, 1.10)	—	Very low
Low-carbohydrate diet	4	0.76 (0.51, 1.14)	4	5 points	0.92 (0.81, 1.04)	Yes	Very low
Vegetable-based low-carbohydrate diet	2	1.02 (0.46, 2.24)	3	5 points	0.98 (0.75, 1.26)	Yes	Very low
Meat-based low-carbohydrate diet	3	0.91 (0.70, 1.17)	3	5 points	0.98 (0.93, 1.03)	Yes^2^	Low
Dietary inflammatory index	3	1.28 (0.97, 1.68)	—			—	Very low

Abbreviations: CI, confidence interval; DASH, Dietary Approaches to Stop Hypertension; HEI, Healthy Eating Index; PDI, plant-based dietary index; SRR, summary risk ratio.

1 Including 1 study from Sweden and 1 study from the Netherlands.

2 Formal testing indicated no nonlinearity, but the curve indicates presence of nonlinearity.

Adherence to the EAT-Lancet planetary health diet was inversely associated with CVD outcomes (SRR: 0.65; 95% CI: 0.45, 0.95; *n* = 3; τ^2^: 0.09; *I*^2^: 96%, Supplemental Figure 6), graded with very low certainty of evidence (Supplemental Table 7).

### Dietary guidelines

We investigated the adherence to the (Alternate) HEI and CVD outcomes in individuals with type 2 diabetes (Figure 2 and Supplemental Figure 7) and observed an inverse association (SRR high compared with low: 0.78; 95% CI: 0.71, 0.86; *n* = 4; τ^2^: 0.0006; PI: 0.62, 0.99; *I*^2^: 5%), graded with low certainty of evidence (Supplemental Table 7). Three studies investigated the adherence to the Chinese dietary guideline, and higher adherence showed an inverse association with CVD outcomes (SRR high compared with low: 0.78; 95% CI: 0.69, 0.88; *n* = 3; τ^2^: 0.005; *I*^2^: 44%, Supplemental Figure 8), graded with low certainty of evidence. Pooling all studies on dietary guidelines (including a study on adherence to the Swedish dietary guidelines and a study on adherence to the Dutch dietary guidelines, Supplemental Figure 9), we found an inverse association with CVD outcomes (SRR high compared with low: 0.80; 95% CI: 0.76, 0.84; *n* = 9; τ^2^: <0.0001; PI: 0.75, 0.85; *I*^2^: 0%, Supplemental Figure 10), graded with low certainty of evidence. A dose–response analysis was not possible due to missing data.

### Nutrient-based dietary patterns

No association was found for glycemic load and glycemic index (Supplemental Figures 11 and 12), graded with moderate and very low certainty of evidence, respectively. A nonlinear dose–response meta-analysis was not possible. We found an inverse association of a low-carbohydrate diet score and CVD outcomes (SRR high compared with low: 0.76; 95% CI: 0.51, 1.14; *n* = 4; τ^2^: 0.099; PI: 0.15, 3.81; *I*^2^: 73%, very low certainty of evidence), and there was an indication for a nonlinear dose–response relationship (Supplemental Figure 13) indicating lower CVD incidence for a moderate adherence to a low-carbohydrate diet. In contrast, no linear dose–response association was found for a vegetable-based (Supplemental Figure 14) or meat-based low-carbohydrate diet (Supplemental Figure 15). However, looking at the nonlinear dose–response of a vegetable-based low-carbohydrate diet, an inverse association with CVD outcomes could be shown for higher adherence (Supplemental Figure 14).

The dietary inflammatory index was positively associated with CVD mortality (SRR high compared with low: 1.28; 95% CI: 0.97, 1.68; *n* = 3; τ^2^: 0.02; *I*^2^: 32%, Supplemental Figure 16), graded with very low certainty of evidence.

## Discussion

The present systematic review and meta-analysis provide a comprehensive overview of the currently available evidence on dietary patterns and risk of CVD exclusively in individuals with type 2 diabetes. We identified 57 studies and conducted 15 meta-analyses. Moderate certainty of evidence was found for the inverse association of a Mediterranean diet and risk of CVD in individuals with type 2 diabetes. We found first indications for other plant-based diets, such as the EAT-Lancet planetary health diet, PDI, and DASH, as well as different national dietary guidelines in association with CVD, rated with low or very low certainty of evidence. In contrast, the dietary inflammatory index and unhealthy plant-based index were positively associated with CVD outcomes, rated with very low and low certainty of evidence, respectively. In contrast, no clear association was found for overall and vegetable-based plant-based index, and vegetable-based and meat-based low-carbohydrate diet. The results of the present meta-analysis indicate no association between glycemic load or glycemic index and CVD in persons with type 2 diabetes. Importantly, the main body of evidence in the present study came from studies at high risk of bias, mainly due to insufficiently controlled confounding, and thus, results should be interpreted with caution.

### Comparison to other studies

So far, there is no systematic review with meta-analysis on dietary patterns and risk of CVD exclusively in individuals with type 2 diabetes available. However, few systematic reviews and meta-analyses investigated single food groups in association with risk of CVD incidence or CVD mortality in individuals with type 2 diabetes. For example, a recent meta-analysis showed inverse associations between higher intakes of fruit (SRR: 0.90; 95% CI: 0.84, 0.97; *n* = 6) and vegetables (SRR: 0.97; 95% CI: 0.94, 0.99; *n* = 2) and CVD mortality in individuals with type 2 diabetes [73]. Another meta-analysis on fish consumption showed an inverse association with the risk of coronary artery disease (SRR per serving of fish per week: 0.92; 95% CI: 0.86, 0.98; *n* = 3) in individuals with type 2 diabetes [74]. These food groups are main components of the Mediterranean diet, which was inversely associated with CVD outcomes in the present meta-analysis. With regard to the general population, evidence for inverse associations between a Mediterranean diet [75], DASH [76], EAT-Lancet diet [77], and PDI [78] and CVD outcomes is available. For instance, a recent systematic review and meta-analysis found a 17% lower relative risk for higher adherence to a Mediterranean-style diet and CVD incidence (SRR: 0.83; 95% CI: 0.81, 0.85; *n* = 21), compared with lower adherence in the general population [75]. Additionally, a recent umbrella review of meta-analyses of randomized controlled trials (RCTs) found that adherence to a Mediterranean diet was beneficial with regard to cardiovascular disease risk factors, such as reducing body weight and triglyceride concentrations in persons with type 2 diabetes [5]. However, no long-term outcomes, such as CVD incidence, are available in these meta-analyses of RCTs. Moreover, another systematic review and meta-analysis showed an inverse association between DASH and CVD incidence and mortality (SRR: 0.81; 95% CI: 0.78, 0.85; *n* = 31) [76]. The results of the present study are in agreement with recommendations from the American Diabetes Association [3] and the European Association for the Study of Diabetes [4], emphasizing a Mediterranean or DASH diet.

Furthermore, with regard to the EAT-Lancet diet and the PDI, meta-analyses found inverse associations with CVD outcomes for higher adherence to the EAT-Lancet diet (SRR: 0.84; 95% CI: 0.81, 0.87) [77] and PDI (SRR: 0.84; 95% CI: 0.79, 0.89)[78]. These observations can be complemented by clinical evidence: the umbrella review of meta-analyses of RCTs showed beneficial effects of plant-based diets on cardiometabolic markers, particularly for the reduction of BMI (in kg/m^2^) and waist circumference [5]. In the present systematic review and meta-analyses, only pairwise meta-analysis was possible for the PDI, showing no association with CVD. However, the dose–response analyses between adherence to a healthy or unhealthy PDI indicated that a healthy PDI, rich in fruit, vegetables, whole grains, nuts, and legumes, is inversely associated with CVD, whereas an unhealthy plant-based index, reflecting a diet rich in sugar-sweetened beverages, sweets, and refined grains, showed a positive association with CVD in persons with type 2 diabetes. Thus, more research on different plant-based indices is needed.

### Strengths and limitations

The major strength of the present systematic review and meta-analysis is the use of rigorous methods to provide a comprehensive overview of the currently available evidence of association between dietary patterns and CVD outcomes in individuals with type 2 diabetes. We conducted a comprehensive systematic literature search through additional citation searching to ensure that all relevant studies were included. Moreover, we contacted many authors for additional data. We used validated tools to assess the risk of bias in all included studies and to evaluate the certainty of evidence of all associations. By including only prospective studies, we minimized some sources of bias, such as recall bias. Moreover, we conducted linear and nonlinear dose–response meta-analyses whenever possible.

However, there are also some limitations that have to be mentioned. First, due to the observational study design of the included studies, unmeasured, unknown, and residual confounding cannot be ruled out, and thus, causal inference is not possible. Second, the exposure was measured by self-reports and mostly only once at study baseline, and thus, potential misclassification of the exposure and changes in dietary behavior over time cannot be ruled out. Third, due to the small number of studies in the meta-analyses, the investigation of sources of heterogeneity based on study characteristics by subgroup analyses and assessment of potential publication bias was not possible. Last, many studies did not explicitly report that individuals with type 1 diabetes have been excluded, but as the proportion of type 1 diabetes is small compared with type 2 diabetes, the number of individuals with type 1 diabetes in the studies is expected to be very low in the studies.

In conclusion, this systematic review and meta-analysis presents first evidence for a potentially beneficial role of several plant-based diets that are rich in vegetables, fruit, whole grains, nuts, and legumes in association with CVD outcomes exclusively in individuals with type 2 diabetes. No clear association was observed for dietary patterns such as total and healthy PDI, and a vegetable-based low-carbohydrate diet. In addition, most meta-analyses were based on a limited number of studies, which restricts the robustness of the findings. Therefore, further well-conducted studies with careful control of relevant confounders are required to strengthen the evidence regarding the association between dietary patterns and risk of CVD in individuals with type 2 diabetes.

## Author contributions

The authors’ responsibilities were as follows – JB, SS: designed the study question and developed the search term of the systematic review and meta-analysis; JB, ES, AL, MN: conducted the systematic literature search; JB, LK, AL, MN: were involved in data acquisition; JB, LK, ES, SS: conducted the assessment of risk of bias; JB, LS: rated the certainty of evidence; JB: conducted the statistical analyses; JB, SS: interpreted the results; JB, SS: drafted the first version of the manuscript; JB, LK, AL, ES, LS, MN, SS: critically reviewed; and all authors: read and approved the final manuscript.

## Data availability

All data included in the present systematic review and meta-analyses are published, extracted data can be found in the supplemental material.

## Declaration of generative AI and AI-assisted technologies in the writing process

The author(s) declare that no generative AI or AI-assisted technologies were used in the writing of this manuscript.

## Funding

This project was founded by the German Research Foundation (DFG), project number: 543196256. The German Diabetes Center (DDZ) is funded by the German Federal Ministry of Health and the Ministry of Science and Culture of the state of North Rhine-Westphalia. The German Center for Diabetes Research (DZD) is funded by the Federal Ministry of Research, Technology and Space. The funders had no role in study design or data collection, analysis, and interpretation.

## Conflict of interest

LS is an editor for Advances in Nutrition and played no role in the journal’s evaluation of the manuscript. All other authors report no conflicts of interest.
