# Supplementary material for: Dietary Patterns and Cardiovascular Diseases in Individuals with Type 2 Diabetes: A Systematic Review and Meta-Analysis of Prospective Observational Studies
Source: Adv Nutr. 2026 Apr 16;17(6):100640. doi: 10.1016/j.advnut.2026.100640 (PMC13187600; doi:10.1016/j.advnut.2026.100640)
Supplement: multimedia component 1 [file mmc1.pdf]

Supplementary material

Dietary patterns and cardiovascular diseases in individuals with type 2 diabetes: a systematic review and meta-analysis of prospective observational studies

Janett Barbaresko, Lisa Kannenberg, Edyta Schaefer, Alexander Lang, Lukas Schwingshackl, Manuela Neuenschwander, Sabrina Schlesinger

Content

Table S1. Detailed eligibility criteria based on PICOS statement 2
Table S2. Search strategy in PubMed, Embase and Cochrane library....3
Table S3. Risk of bias judgements for each domain using the Risk Of Bias In Non-randomized Studies - of Exposures (ROBINS-E) tool....5
Table S4. List of included and excluded articles with reasons for exclusion. 10
Table S5. Characteristics of included prospective observational studies. 11
Table S6. Risk of bias of included studies using the Risk Of Bias In Non-randomized Studies - of Exposure (ROBINS-E) tool. 24
Table S7. Certainty of evidence for associations of dietary factors and cardiovascular diseases in individuals with type 2 diabetes. 26
Figure S1. Forest plots for the association between the Mediterranean diet and cardiovascular diseases. A) high vs low adherence, B) linear-dose-response (per 1 point) and C) non-linear dose-response meta-analysis....28
Figure S2. Forest plots for the association between Dietary Approaches to Stop Hypertension and cardiovascular diseases. A) high vs low adherence, B) linear-dose-response (per 5 points) and C) non-linear dose-response meta-analysis....29
Figure S3. Forest plot for the association between the plant-based dietary index and cardiovascular diseases (high vs low meta-analysis). 29
Figure S4. Forest plots for the association between the healthy plant-based dietary index and cardiovascular diseases. A) high vs low adherence, and B) linear-dose-response (per 5 points)....30
Figure S5. Forest plots for the association between the unhealthy plant-based dietary index and cardiovascular diseases. A) high vs low adherence, and B) linear-dose-response (per 5 points)....30
Figure S6. Forest plot for the association between the EAT-Lancet planetary health diet and cardiovascular diseases (linear dose-response per 3 points)....31
Figure S7. Forest plot for the association between adherence to the (alternate) Healthy Eating Index and cardiovascular diseases (high vs low adherence)....31
Figure S8. Forest plot for the association between adherence to the Chinese Dietary Guideline and cardiovascular diseases (high vs low adherence). 31
Figure S9. Forest plot for the association between adherence to European dietary guidelines and cardiovascular diseases (high vs low adherence). 31
Figure S10. Forest plots for the overall association between adherence to National dietary guidelines and cardiovascular diseases (high vs low adherence)....32
Figure S11. Forest plot for the association between glycemic load and cardiovascular diseases (linear-dose-response per 5 units)....32
Figure S12. Forest plot for the association between glycemic index and cardiovascular diseases (linear-dose-response per 5 units)....32
Figure S13. Forest plots for the association between adherence to a low-carbohydrate diet and cardiovascular diseases. A) high vs low adherence, B) linear-dose-response (per 5 points) and C) non-linear dose-response meta-analysis....33
Figure S14. Forest plots for the association between adherence to a vegetable-based low-carbohydrate diet and cardiovascular diseases. A) high vs low adherence, B) linear-dose-response (per 5 points) and C) non-linear dose-response meta-analysis. 34
Figure S15. Forest plots for the association between adherence to a meat-based low-carbohydrate diet and cardiovascular diseases. A) high vs low adherence, B) linear-dose-response (per 5 points) and C) non-linear dose-response meta-analysis. 35
Figure S16. Forest plot for the association between adherence to the dietary inflammatory index and cardiovascular diseases (high vs low adherence). 35
References 36

Table S1. Detailed eligibility criteria based on PICOS statement

	Inclusion criteria	Exclusion criteria
**P** (population)	Persons with type 2 diabetes aged 18 or older	Studies which solely focused on children, adolescents, participants with prediabetes, type 1 diabetes or gestational diabetes, participants with prevalent cardiovascular diseases
**I** (intervention/exposure)<br><br>**C** (comparison)	Any dietary pattern, including a priori dietary patterns such as dietary indices (e.g., Healthy Eating Index (HEI) or plant-based dietary index) and dietary scores (e.g., Mediterranean Diet Score, low-carbohydrate diet score) as well as exploratory dietary patterns (e.g., derived by principal component analysis) and hybrid methods such as reduced rank regression;<br>comparing high to low adherence as well as dose-response relationships	Studies reporting on dietary patterns solely in combination with other lifestyle factors (e.g., physical activity, lifestyle index), or solely focusing on single foods/food groups (e.g., only fruit and vegetables)
**O** (outcome)	Cardiovascular and cerebrovascular incidence and mortality, including non-fatal and fatal coronary heart diseases, heart failure and stroke	Non-cardiovascular outcomes, hypertension, biomarkers.
**S** (study design)	Prospective observational studies (including cohort, nested case-control, case-cohort studies and follow-up studies of intervention studies) published in a peer-reviewed journal.	Interventional studies, cross-sectional and case-control studies, case only studies, animal and cell culture studies, conference abstracts, comments, letters and reviews.

Table S2. Search strategy in PubMed, Embase and Cochrane library (last update on 10 January 2025)

**PubMed**		
#1	Exposure	diet [Mesh] OR diet [tiab] OR dietary [tiab] OR intake [tiab] OR consumption [tiab] OR food [tiab] OR foods [tiab] OR food [MeSH] OR beverage [tiab] OR beverages [tiab] OR beverages [Mesh] OR drink [tiab] OR drinking [tiab] OR bread [tiab] OR cereals [tiab] OR grains [tiab] OR whole-grain [tiab] OR soy [tiab] OR soya [tiab] OR potatoes [tiab] OR legumes [tiab] OR rice [tiab] OR pasta [tiab] OR vegetables [tiab] OR fruit [tiab] OR milk [tiab] OR dairy [tiab] OR eggs [tiab] OR meat [tiab] OR fish [tiab] OR seafood [tiab] OR nuts [tiab] OR sweets [tiab] OR nutrient [tiab] OR nutrients [tiab] OR coffee [tiab] OR tea [tiab] OR juice [tiab] OR macronutrients [tiab] OR fats [tiab] OR "fatty acids" [tiab] OR carbohydrates [tiab] OR fibre [tiab] OR fiber [tiab] OR sugar [tiab] OR protein [tiab] OR micronutrients [tiab] OR vitamin [tiab] OR vitamins [tiab] OR mineral [tiab] OR minerals [tiab] OR polyphenols [tiab]
#2	Population	diabetes [tiab] OR "Diabetes Mellitus, Type 2" [Mesh] OR diabetic [tiab]
#3	Outcomes	"Myocardial Ischemia"[Mesh] OR "Heart Failure"[Mesh] OR "Stroke"[Mesh] OR macrovascular [tiab] OR "cardiovascular disease" [tiab] OR "cardiovascular diseases" [tiab] OR CVD [tiab] OR "heart disease" [tiab] OR "myocardial infarction" [tiab] OR "heart failure" [tiab] OR “cerebrovascular diseases” [tiab] OR “peripheral artery disease” OR stroke [tiab] OR mortality [tiab] OR death [tiab]
#4	Study design	prospective [tiab] OR cohort [tiab] OR "Cohort Studies" [Mesh] OR follow-up [tiab] OR longitudinal [tiab] OR nested [tiab]
#5		#1 AND #2 AND #3 AND #4
**Embase**		
#1	Exposure	'diet'/exp OR 'dietary pattern'/exp OR 'dietary intake'/exp OR 'food'/exp OR 'nutrient'/exp OR diet:ti,ab OR dietary:ti,ab OR intake:ti,ab OR consumption:ti,ab OR food:ti,ab OR foods:ti,ab OR beverage:ti,ab OR beverages:ti,ab OR drink:ti,ab OR drinking:ti,ab OR bread:ti,ab OR cereals:ti,ab OR grains:ti,ab OR 'whole grain':ti,ab OR soy:ti,ab OR soya:ti,ab OR potatoes:ti,ab OR legumes:ti,ab OR rice:ti,ab OR pasta:ti,ab OR vegetables:ti,ab OR fruit:ti,ab OR milk:ti,ab OR dairy:ti,ab OR eggs:ti,ab OR meat:ti,ab OR fish:ti,ab OR seafood:ti,ab OR nuts:ti,ab OR sweets:ti,ab OR nutrient:ti,ab OR nutrients:ti,ab OR coffee:ti,ab OR tea:ti,ab OR juice:ti,ab OR ((dietary:ti,ab OR intake:ti,ab OR consumption:ti,ab) AND (macronutrients:ti,ab OR fats:ti,ab OR 'fatty acids':ti,ab OR carbohydrates:ti,ab OR fibre:ti,ab OR fiber:ti,ab OR sugar:ti,ab OR protein:ti,ab OR micronutrients:ti,ab OR vitamin:ti,ab OR vitamins:ti,ab OR mineral:ti,ab OR minerals:ti,ab OR polyphenols:ti,ab))
#2	Population	'non insulin dependent diabetes mellitus'/exp OR diabetes:ti,ab
#3	Outcome	'ischemic heart disease'/exp OR 'cerebrovascular accident'/exp OR 'heart failure'/exp OR 'diabetic heart disease'/exp OR macrovascular:ti,ab OR ‘cardiovascular disease’:ti,ab OR ‘cardiovascular diseases’:ti,ab OR CVD:ti,ab OR ‘heart disease’:ti,ab OR ‘myocardial infarction’:ti,ab OR ‘heart failure’:ti,ab OR ‘peripheral artery disease’:ti,ab OR ‘cerebrovascular diseases’:ti,ab OR stroke:ti,ab OR mortality:ti,ab OR death:ti,ab
#4	Study design	'cohort analysis'/exp OR prospective:ti,ab OR cohort:ti,ab OR follow-up:ti,ab OR longitudinal:ti,ab OR nested:ti,ab
#5		#1 AND #2 AND #3 AND #4
**Cochrane library**		
#1	Exposure	(1) MeSH descriptor: [Diet] explode all trees<br>(2) MeSH descriptor: [Food and Beverages] explode all trees<br>(3) diet OR dietary OR intake OR consumption OR food OR foods OR beverage OR beverages OR drink OR drinking OR bread OR cereals OR grains OR 'whole grain' OR soy OR soya OR potatoes OR legumes OR rice OR pasta OR vegetables OR fruit OR milk OR dairy OR eggs OR meat OR fish OR seafood OR nuts OR sweets OR nutrient OR nutrients OR coffee OR tea OR juice OR macronutrients OR fats OR 'fatty acids' OR carbohydrates OR fibre OR fiber OR sugar OR protein

		OR micronutrients OR vitamin OR vitamins OR mineral OR minerals OR polyphenols<br>(1) OR (2) OR (3)
#2	Population	(1) MeSH descriptor: [Diabetes Mellitus, Type 2] explode all trees<br>(2) diabetes<br>(1) OR (2)
#3	Outcome	(1) MeSH descriptor: [Heart Diseases] explode all trees<br>(2) MeSH descriptor: [Vascular Diseases] explode all trees<br>(3) macrovascular OR ‘cardiovascular disease’ OR ‘cardiovascular diseases’ OR CVD OR ‘heart disease’ OR ‘myocardial infarction’ OR ‘heart failure’ OR ‘peripheral artery disease’ OR ‘cerebrovascular diseases’ OR stroke OR mortality OR death<br>(1) OR (2) OR (3)
#4	Study design	(1) MeSH descriptor: [Cohort Studies] explode all trees<br>(2) prospective OR cohort OR follow-up OR longitudinal OR nested<br>(1) OR (2)
#5		#1 AND #2 AND #3AND #4

Table S3. Risk of bias judgements for each domain using the Risk Of Bias In Non-randomized Studies - of Exposures (ROBINS-E) tool (adapted from Higgins *et al.*(1))

Domain	Explanation	Judgements
**Risk of bias due to confounding**	• Is there potential for confounding of the effect of exposure in this study?<br>• Did the authors use a multivariable-adjusted analysis method that controlled at least for age, sex, education/socioeconomic status, smoking, physical activity, total energy intake and diabetes duration/diabetes medication?<br>• Were confounding factors that were controlled for measured validly and reliably by the variables available in this study?<br>• Did the authors avoid adjusting for post-exposure variables?<br><br>*Notes:* Confounding is expected in all observational studies; thus, no study was assigned low risk of bias. Time-varying confounding was expected to be unlikely and is not expected to cause risk of bias in the present study.<br>If a study was rated with high risk of bias in this domain, the study was overall rated as high risk of bias and no further assessment was performed.	<u>Low risk of bias:</u><br>No bias is expected due to confounding, including time-varying confounding.<br><br><u>Some concerns:</u><br>Confounding is expected for age, sex, education/socioeconomic status, smoking, physical activity, total energy intake, diabetes duration/diabetes medication, and the authors performed a multivariable-adjusted analysis to control for these confounding factors. The variables adjusted for are valid and reliable measures of the confounding factors;<br>*or*<br>one confounding factor is the only important covariate not included in the multivariable-adjusted analysis, but this factor is not expected to vary substantially within the cohort (e.g. education/ socioeconomic status in certain occupational cohorts like Nurses Health' Study or Health Professionals Follow-Up Study);<br>*or*<br>the authors statistically investigated whether the confounding factors have an effect on the risk estimate and excluded the confounder from the multivariable model if there was no effect on the overall effect estimate.<br><br><u>High risk of bias:</u><br>At least one known important confounding factor was not measured or appropriately controlled for;<br>*or*<br>the authors adjusted for post-exposure variables that are affected by exposure (e.g. dietary intake and risk of cardiovascular diseases [adjustment for anthropometric measures during follow-up = intermediate biological variable on the causal pathway] → overadjustment).
**Risk of bias arising from measurement of exposure**	• Does the measured exposure well-characterize the exposure metric specified to be of interest in this study?<br>• Was the exposure likely to be measured with error, or misclassified?	<u>Low risk of bias:</u><br>The exposure status is well characterised by the measurement and no measurement error is expected in its assessment (e.g., use of biomarkers for dietary intake);<br>*or*<br>the exposure was measured at multiple times, and is stable or changes only slightly over time.

	Notes: Differential misclassification is not expected to occur in prospective cohort studies, since dietary intake was reported before the occurrence of the outcome (Freedman 2011).<br>Some type of non-differential misclassification cannot be excluded (any dietary assessment method involves measurement error), thus no study was assigned low risk of bias.	<u>Some concerns:</u><br>The exposure status is well characterised by the measurement, and was measured using an established or validated tool (e.g., a validated food frequency questionnaires, multiple 24h recalls);<br>*and*<br>the exposure was measured at multiple times, and it is stable or changes only slightly over time;<br>*or*<br>the exposure was measured by a single measurement assessing longer periods of time (i.e. validated food frequency questionnaires), and is therefore assumed to be stable over time.<br><br><u>High risk of bias:</u><br>The exposure status is not well characterized by the measurement (e.g., assumed from an indirect measurement or important sources of dietary intake are not considered);<br>*and/or*<br>the exposure was measured using not validated subjective measurements;<br>*and/or*<br>the exposure was measured with a single measurement, which is unlikely to characterize the exposure over a longer period of time (e.g., single 24h recall) and therefore cannot be assumed to be representative;<br>*and/or*<br>the exposure cannot be assumed to be stable over time.
**Risk of bias in selection of participants into the study**	• Did follow-up begin at (or close to) the start of the exposure window for most participants?<br>• Is the effect of exposure likely to be constant over the period of follow up analysed?<br>• Was selection of participants into the study (or into the analysis) based on participant characteristics observed after the start of the exposure window being studied?<br>• Is it likely that the analysis corrected for all of the potential selection biases identified above?<br>• Did sensitivity analyses demonstrate that the likely impact of the potential selection biases identified in A or B above was minimal?	<u>Low risk of bias:</u><br>All participants who would have been eligible for the target study were included in the study;<br>*and*<br>the main part of the cohort (>50%) consists of participants with newly diagnosed type 2 diabetes (<1 year).<br><br><u>Some concerns:</u><br>Inclusion of not newly diagnosed participants (>1 year) with diabetes; and/or the selection into the study may have been related to exposure and outcome (e.g. patients with further comorbidities);<br>*and*<br>the authors used appropriate methods to correct for the selection bias (e.g., adjustment for diabetes duration);<br>*and/or*

	• Is the risk of bias (due to selection of participants into the study) sufficiently high, in the context of its likely direction and the magnitude of the estimated exposure effect, to threaten conclusions about whether the exposure has an important effect on the outcome?<br><br>Notes: In observational studies, it is unlikely that post-exposure variables influenced selection of participants into the study. Exclusion of participants may be mostly based on missing data, which will be considered in the domain referring to missings (see below). The start of follow-up is considered to coincide with the baseline exposure assessment. However, participants are already exposed at start of the study which might have influenced outcome measured that occurred shortly after start of the study.	the authors conducted a sensitivity analysis excluding the cases which occurred <2 years after start and results did not change substantially.<br><br>High risk of bias:<br>Selection into the study was related to exposure and outcome (e.g. only participants with specific comorbidities such as chronic kidney disease were included in the analysis);<br>*and/or*<br>inclusion of not newly diagnosed participants with diabetes (>1 year); and a potentially important amount of follow-up time may be missing from the analyses (e.g. no sensitivity analysis excluding the first two years of follow-up or excluding prevalent other chronic diseases than diabetes has been conducted); and/or the rate ratio is not constant over time;<br>*and*<br>This could not be corrected for in the analyses.
**Risk of bias due to post-exposure interventions**	• Were there post-exposure interventions that were influenced by prior exposure during the follow-up period?<br>• Is it likely that analysis corrected for the effect of post-exposure interventions?<br><br>*Notes:* In prospective observational studies, post-exposure interventions are unlikely. We don't expect any issues in this domain for our analysis.	Low risk of bias:<br>There were (probably) no interventions administered to alleviate the effect of exposures.<br><br>Some concerns:<br>Post-exposure interventions were identified and the analysis corrected for the effect of these interventions.<br><br>High risk of bias:<br>Post-exposure interventions were identified and the analysis did not correct for the effect of these interventions
**Bias due to missing data**	• Were complete data on exposure status available for all, or nearly all, participants?<br>• Were complete data on the outcome available for all, or nearly all, participants?<br>• Were complete data on confounding variables available for all, or nearly all, participants?<br>• Did the authors perform a complete case analysis?<br>• Was an appropriate method used to correct for bias due to missing data?	Low risk of bias:<br>There was little loss-to-follow-up (<20%);<br>*and*<br>data on exposure and other variables were reasonably complete (<10% missing data) and was unlikely to introduce bias;<br>*or*<br>the analysis addressed missing data and is likely to have removed any risk of bias.<br><br>Some concerns:<br>There is a proportion of missing data (>10%) in the original cohort or a high proportion of loss-to-follow-up (>20%);

<table>
<tr><td></td><td>Notes: Missing data on exposure variables and other variables are expected to be missing at random and not related to exposure or outcome that have been assessed during follow-up.</td><td>and
the analysis is unlikely to have removed the risk of bias arising from the missing data (e.g., using logistic regression);
or
there is a significant proportion (>20%) of missing data but the authors addressed this issue by appropriate methods (e.g., imputation of data).

<u>High risk of bias:</u>
There are high proportions (>50%) of missing data.
and
The analysis is unlikely to have removed the risk of bias arising from the missing data.
or
The nature of the missing data means that the risk of bias cannot be removed through appropriate analysis.</td></tr>
<tr><td>Risk of bias due to measurement of the outcome</td><td>• Could measurement or ascertainment of the outcome have differed between exposure groups or levels of exposure?
• Were outcome assessors aware of study participants' exposure history?
• Could assessment of the outcome have been influenced by knowledge of participants' exposure history?
• Was any systematic error in measurement of the outcome related to exposure status?</td><td><u>Low risk of bias:</u>
The methods of outcome assessment were comparable across all exposure groups;
and
the outcome measure was unlikely to be influenced by knowledge of the exposure status of study participants;
and
any error in measuring the outcome is unrelated to exposure status (i.e. objective measures or self-reported outcomes that are mostly (≥90%) confirmed by a second source, e.g. medical records, record linkage, death certificates).

<u>Some concerns:</u>
The methods of the outcome assessment were comparable across exposure groups;
and
any error in measuring the outcome may be minimally related to exposure status;
or
the measurement of the outcome is not reliable (i.e. confirmed records are available for <90% of all participants and the authors did not perform an additional analysis separating confirmed and probable cases).

<u>High risk of bias:</u>
The methods of outcome assessment were not comparable across exposure groups;
or
the outcome measure was subjective (i.e. self-reports by study participants or next of kin, without confirmation by a second source);
and/or
any error in measuring the outcome was related to exposure status.</td></tr>
</table>

<table>
<tr><td>Risk of bias due to selection of the reported result</td><td>Was the result reported in accordance with an available, pre-determined analysis plan?
Is the reported effect estimate likely to be selected, based on desirability of the magnitude (or statistical significance) of the estimated effect of exposure on outcome,
- from multiple exposure measurements within the exposure domain?
- from multiple outcome measurements within the outcome domain
- from multiple analyses of the exposure-outcome relationship?
Is the reported effect estimate likely to be selected, based on the basis of desirability of the results (e.g., statistical significance), from different subgroups?
Notes: In observational studies, it is unusual to publish an a priori analysis plan or protocol. Therefore, if the authors present a clear description of the conducted analyses in the methods, and it appears to be consistent with the reported results; and the reported results correspond to all intended outcomes, analyses and subcohorts (e.g. postmenopausal women), low risk of bias can be adequate.</td><td><u>Low risk of bias:</u>
The results are reported according to an a-priori analysis plan or protocol;
or
there is a clear description of all analysis, the analyses are consistent, and all reported results correspond to all intended outcomes, analyses and sub-cohorts.

<u>Some concerns:</u>
There is an a-priori analysis plan or protocol available, and there is indication of selection of the reported analysis among multiple analyses; or there is indication of selection of the cohort or subgroups for analysis and reporting on basis of the results (e.g. estimates not shown for all analyses).
or
there is no a-priori analysis plan or protocol and there appears to be no issues with the exposure, multiple analyses (e.g., effect estimates were similar when different multiple analyses were used), or the selection or definition of subgroups, but there are inconsistencies/or no information between intended and reported analyses.

<u>High risk of bias:</u>
There is a high risk of selective reporting from multiple exposure measurements, outcomes measurements, or multiple analyses of data;
or
the cohort or subgroup is selected from a larger study for analysis and appears to be reported based on the results.</td></tr>
<tr><td rowspan="3">Overall judgement</td><td>Low risk of bias</td><td>The study is judged to be at low risk of bias for all domains.</td></tr>
<tr><td>Some concerns</td><td>The study is judged to be at low risk of bias or some concerns for all domains.</td></tr>
<tr><td>High risk of bias</td><td>The study is judged to be at high risk of bias in at least one domain, but no domains are at very high risk of bias.</td></tr>
</table>

Table S4. List of included and excluded articles with reasons for exclusion.

	**References**
**All included studies (including hand search)**	(2-58)
**Excluded studies with reasons for exclusion**	
Not relevant population	(59-428)
Not relevant exposure	(429-632)
Not relevant outcome	(633-674)
Not relevant study design	(675-686)
Conference abstracts	(687-931)
Editorial/letter, review	(932, 933)
Retracted articles	(934, 935)
Double cohort	(936-938)

Table S5. Characteristics of included prospective observational studies.

Author, year	Cohort name, country, Follow-up	Number of participants, sex, age	Exposure assessment	Outcome assessment	Outcome	N cases	Exposure(s)	Exposure categories	RR (95% CI)	Adjustment factors
Bonaccio, 2016 (2)	Moli-sani study, Italy, 4 y	1,995 men and women with T2D, 62.6 y	FFQ	Italian mortality registry, validated by Italian death certificates	CVD mortality	51	Mediterranean diet score	Poor (0-3)<br>Average (4–5)<br>High (≥6)<br><br>Per 2-point increase	1.00<br>0.71 (0.37, 1.38)<br>0.43 (0.19, 0.99)<br><br>0.66 (0.46, 0.95)	Age, sex, education, total energy intake, leisure-time physical activity, smoking, years from diagnosis of diabetes, blood glucose, hypercholesterolaemia
Burger, 2012 (3)	EPIC, Europe, 9.2 y	6,192 men and women with T2D, 57.4 y	FFQ	Follow-up mailings and subsequent inquiries to municipal registries, regional health departments, physicians, hospitals, record linkages with local, regional, central cancer registries, boards of health, death indexes	CVD mortality	306	Glycemic load	per 22.0 g/d	0.95 (0.78, 1.15)	Age, sex, country, smoking, education, BMI, WHR, physical activity, menopausal status, HRT use, alcohol, diabetes duration, insulin use, HbA1c, total energy, energy-adjusted nutrients, vitamin C, saturated, MUFA, PUFA, fiber intake;<br>Glycemic index also adjusted for energy-adjusted carbohydrate intake
							Glycemic index	per 3.9	0.96 (0.85,1.10)	
Chen, 2025 (4)	UK Biobank, UK, 12.1 y	9,942 men and women with diabetes, 58.9 y	24h dietary recalls	Death certificates	CVD mortality	323	Dietary inflammatory index	Q1<br>Q2<br>Q3<br>Q4<br><br>Per unit	1.00<br>1.15 (0.81, 1.65)<br>1.48 (1.03, 2.12)<br>1.67 (1.13, 2.48)<br><br>1.13 (1.04, 1.21)	Age, sex, total energy intake, BMI, ethnicity, education, Townsend Deprivation Index, alcohol, smoking, physical activity, diabetes duration
Dai, 2024 (5)	NHANES, USA, 9.3 y	2,911 men and women with diabetes, 57.4 y	24h dietary recalls	Linkage with the National Death Index	CVD mortality	190	Glycemic load (per 10 units)	GL in early morning<br>GL in late morning<br>GL in afternoon<br>GL in evening<br>GL at night<br><br>Substitution with 10 units GL in late morning<br>- in early morning<br>- in afternoon<br>- in evening<br><br>Substitution with 10 units GL in early morning<br>- in afternoon<br>- in evening<br><br>Substitution with 10 units GL in afternoon<br>- in evening	1.03 (0.91, 1.16)<br>0.86 (0.77, 0.95)<br>1.07 (0.95, 1.20)<br>0.98 (0.87, 1.12)<br>1.44 (1.17, 1.77)<br><br><br>0.90 (0.79, 1.03)<br>0.84 (0.74, 0.95)<br>0.86 (0.75, 1.00)<br><br><br>0.94 (0.82, 1.06)<br>0.96 (0.82, 1.12)<br><br><br>1.03 (0.90, 1.17)	Age, sex, ethnicity, educational attainment, ratio of family income to poverty, health insurance, smoking status, alcohol intake, physical activity, BMI, hypertension, dyslipidemia, family history of heart disease, glucose-lowering medication use, diabetes duration, total energy intake, energy-adjusted intake of total GL, fiber, total fat, SFA, MUFA, PUFA
Damigou, 2025 (6)	ATTICA, Greece, 20 y*	226 men and women with T2D, 45.2 y*	FFQ	Through study physicians, medical records	Total CVD	10	EAT-Lancet index	per 1 point	0.58 (0.35, 0.98)	Age, sex, BMI, socio-economic status, medical history, smoking habits, physical activity

Damigou, 2025 (7)	ATTICA, Greece, 20 y*	445 men and women with T2D, 45.2 y*	FFQ	Through study physicians, medical records	CVD incidence	330	Mediterranean diet score	Per 1 point	0.33 (0.02, 4.94)	Age, sex, BMI, socio-economic status, medical history, smoking habits, physical activity
Dehghan, 2012 (8)	ONTARGET and TRANSCEND, International, 4.7 y*	12,869 men and women with diabetes, 66.5 y*	FFQ	Na	Total CVD	na	mAHEI	Q1: 16<br>Q2: 20.5<br>Q3: 24.3<br>Q4: 28.7<br>Q5: 35.7	1.00<br>0.96 (0.85, 1.09)<br>0.91 (0.80, 1.04)<br>0.86 (0.75, 0.99)<br>0.75 (0.65, 0.87)	Age, sex, region, trial enrollment allocation, education, smoking, physical activity, BMI, blood pressure, history of hypertension, and stroke, β-blockers, calcium channel blockers, antiplatelets, statin
							Dietary Risk Score (*a posteriori*-based)	0-1<br>2<br>3<br>4<br>5	1.00<br>0.92 (0.79, 1.06)<br>0.89 (0.77, 1.04)<br>0.94 (0.79, 1.12)<br>0.67 (0.48, 0.92)	
Del Gobbo, 2015 (9)	CHS, USA, 21.5 y*	na, men and women with diabetes, 72 y*	FFQ	Medical records, diagnostic tests, clinical consultations, interviews	Heart failure	na	Biological dietary pattern	Q1: 11 - 24<br>Q5: 37-49	1.00<br>0.91 (0.64, 1.30)	Age, sex, race, enrolment site, education, annual income, total kcal expended, walking pace, smoking, alcohol intake, BMI, prevalent treated hypertension, prevalent coronary heart disease
							DASH score	Q1: 9-19<br>Q5: 30-38	1.00<br>0.96 (0.66, 1.38)	
							AHEI score	Q1: 5.5-27.5<br>Q5: 52.5-80.5	1.00<br>0.84 (0.57, 1.24)	
							AHA 2020 score	Q1: 7-37<br>Q5: 63-77	1.00<br>0.90 (0.63, 1.28)	
Deng, 2017 (10)	NHANES III, USA, 11.3 y	968 men and women with T2D, 61.5 y	24-h dietary recall	National Death Index	CVD mortality	240	Dietary inflammatory index	T1<br>T2<br>T3	1.00<br>0.91 (0.60, 1.38)<br>0.98 (0.57, 1.67)	Age, sex, race, HbA1C, current smoking, physical activity, BMI, SBP
Fan, 2024 (11)	NHANES, USA, 7.8 y	3,801 men and women with T2D, 57.4 y	24h dietary recalls	National Death Index	CVD mortality	277	Dietary oxidative balance score	Q1: 7.6<br>Q2: 13.3<br>Q3: 18.4<br>Q4: 24.2	1.00<br>0.89 (0.54, 1.44)<br>0.59 (0.35, 0.99)<br>0.40 (0.21, 0.78)	Age, sex, ethnicity, income-poverty ratio, education, marital status, HbA1c, antidiabetic medication use, CVD, hypertension, CKD, TG, TC, HDL-C
Gamba, 2023 (12)	CoLaus study, Switzerland, 9 y*	326 men and women with T2D, 57.2 y*	FFQ	Medical records, medical databases, population register, general practitioners, death certificates	CVD (fatal and non-fatal)	na	Healthy Dietary Phytochemical Index	T1:8-14<br>T2: 19-24<br>T3: 31-43	1.00<br>0.95 (0.47, 1.91)<br>0.92 (0.43, 1.99)	Age, sex, education, smoking, alcohol, physical activity, BMI, total energy intake (only for hPDI), dieting, hypertension, hypercholesterolemia, family history of CVD
							Dietary Phytochemical Index	T1: 12-18<br>T2: 23-28<br>T3: 35-46	1.00<br>0.74 (0.36, 1.50)<br>0.92 (0.44, 1.90)	
							hPDI	T1: 39-44<br>T2: 47-50<br>T3: 53-59	1.00<br>0.95 (0.49, 1.84)<br>0.76 (0.35, 1.64)	
Gao, 2023 (13)	NHANES, USA, na	4,951 men and women with diabetes, 47.4 y*	24h dietary recalls	National Center for Health Statistics	CVD mortality	na	DII	T1:-5.28 - 0.72<br>T2: 0.73 - 2.51<br>T3: 2.51 - 5.80	1.00<br>1.41 (1.08, 1.83)<br>1.19 (0.89, 1.59)	Age, sex, education, marital status, family poverty income ratio, hypertension, smoking, alcohol, CHD, congestive HF, angina pectoris, MI, stroke, SBP, DBP, BMI, waist circumference, eGFR, hemoglobin, FBG, HDL-C, total cholesterol, triglyceride, blood urea nitrogen, uric acid, serum creatinine

Ghaemi,<br>2021 (14)	NPPCD,<br>Iran,<br>na	20,338 men and women with T2D,<br>59.9 y	14-point Mediterranean Diet Adherence Screener	physician-diagnosed nonfatal CAD, noninvasive tests, self reports	CVD	na	Mediterranean diet	Non-adherence<br>Adherence	1.00<br>0.61 (0.57, 0.89)	Age, sex, time, HbA1c, FBG, HDL, total cholesterol, triglyceride, SBP, obesity, use of statin, smoking, duration of diabetes
Han,<br>2021 (15)	CBK,<br>China,<br>11.2 y	14,215 men and women with T2D,<br>51.2 y*	FFQ	Linkages to disease and mortality registries, national health insurance claim data base and annual active confirmation, death certificates	CVD	2,094	Dietary behaviour	Healthy<br>Unhealthy (non-daily eating of vegetables, fruits, eggs, and eating red meat daily or less than weekly)	1.00<br>0.95 (0.69, 1.31)	Age, study area, sex, education, marital status, parental family history of cardiometabolic multimorbidity, smoking, physical activity, BMI, WHR, alcohol (for diet), healthy diet (for alcohol)
Hardy,<br>2010 (16)	ARIC,<br>USA,<br>17 y	1,378 men and women with diabetes,<br>55.4 y	FFQ	Discharge hospital lists, death certificates, next-to-kin interviews, physician-completed questionnaires	CHD incidence	371	Glycemic index	per 5-units<br><br>per 5-units	Whites:<br>1.01 (0.86, 1.16)<br><br>African americans:<br>1.10 (0.86, 1.40)	Age, sex, BMI, SBP, total cholesterol, HDL-C, anti-hypertensive medications, smoking, sports activity index, Keys dietary score, FBG, total calories on energy-adjusted glycemic index or last, propensity score with glycemic index or last as exposure
							Glycemic load	per 30-units<br><br>per 30-units	Whites:<br>1.03 (0.83, 1.29)<br><br>African americans:<br>1.24 (0.90, 1.71)	
Harriss,<br>2007 (17)	MCCS,<br>Australia,<br>10.4 y	1,524 men and women with diabetes,<br>na	FFQ	Victorian Cancer Registry, Australian Bureau of Statistics	IHD mortality	na	Mediterranean dietary pattern	Q1<br>Q2<br>Q3<br>Q4	1.00<br>0.42 (0.18, 0.97)<br>0.40 (0.17, 0.97)<br>0.21 (0.09, 0.47)	Sex, country of birth, activity, daily energy intake, education, smoking, social isolation, dietary factors, CVD history, and family history of CVD
							Vegetable dietary pattern	Q1<br>Q2<br>Q3<br>Q4	1.00<br>2.32 (1.02, 5.26)<br>2.50 (1.07, 5.84)<br>1.74 (0.71, 4.27)	
He,<br>2023 (18)	UK Biobank,<br>UK,<br>12.1 y	13,543 men and women with T2D,<br>59.6 y	Baseline touchscreen questionnaire	Linkage with hospital admission data and death register records	Total CVD	3,279	Healthy diet score	Poor<br>Ideal	1.00<br>0.88 (0.80, 0.97)	Age, sex, ethnicities, SBP, glycated hemoglobin, non-HDL-C, Townsend deprivation index, family history of diabetes, smoking, alcohol, BMI, physical activity, sleep duration
Hirahatake,<br>2019 (19)	WHI,<br>USA,<br>12.4 y	5,809 women with T2D,<br>64 y	FFQ	Medical questionnaires, medical records, outpatient coronary revascularization procedures	CVD	1,454	DASH diet	Q1: 18.9<br>Q2: 23.1<br>Q3: 25.5<br>Q4: 27.9<br>Q5: 31.7<br><br>per SD	1.00<br>0.85 (0.72, 0.99)<br>0.85 (0.72, 1.01)<br>0.87 (0.75, 1.02)<br>0.69 (0.58, 0.83)<br><br>0.90 (0.85, 0.95)	Age, race, education, income, marital status, physical activity, smoking, BMI, WHI study arm, geographical region, age at T2D diagnosis, energy intake, insulin use, blood pressure, history of high cholesterol, smoking x time, SBP x time, DBP x time, DASH model additionally adjusted for alcohol
							Alternate Mediterranean diet score	Q1: 1.6<br>Q2: 3.0<br>Q3: 4.0<br>Q4: 5.0<br>Q5: 6.5<br><br>per SD	1.00<br>0.90 (0.77, 1.05)<br>0.86 (0.73, 1.01)<br>0.82 (0.69, 0.97)<br>0.77 (0.65, 0.93)<br><br>0.92 (0.87, 0.98)	
							ADA diet	Q1: 24.1	1.00	

								Q2: 27.5<br>Q3: 30.0<br>Q4: 32.9<br>Q5: 36.7<br><br>per SD	0.89 (0.75, 1.06)<br>0.69 (0.59, 0.81)<br>0.76 (0.64, 0.90)<br>0.71 (0.59, 0.86)<br><br>0.87 (0.82, 0.93)	
							Paleo diet	Q1: 32.0<br>Q2: 37.1<br>Q3: 40.0<br>Q4: 43.5<br>Q5: 48.6<br><br>per SD	1.00<br>1.08 (0.92, 1.27)<br>1.07 (0.90, 1.26)<br>0.90 (0.76, 1.06)<br>0.91 (0.75, 1.09)<br><br>0.96 (0.90, 1.01)	
					CHD	635	DASH diet	Q1: 18.9<br>Q2: 23.1<br>Q3: 25.5<br>Q4: 27.9<br>Q5: 31.7<br><br>per SD	1.00<br>0.93 (0.73, 1.19)<br>0.88 (0.68, 1.14)<br>0.89 (0.69, 1.13)<br>0.75 (0.57, 0.98)<br><br>0.90 (0.82, 0.98)	Age, race, education, income, marital status, physical activity, smoking, BMI, WHI study arm, geographical region, age at T2D diagnosis, energy intake, insulin use, blood pressure, history of high cholesterol, DASH model additionally adjusted for alcohol
							Alternate Mediterranean diet score	Q1: 1.6<br>Q2: 3.0<br>Q3: 4.0<br>Q4: 5.0<br>Q5: 6.5<br><br>per SD	1.00<br>0.75 (0.59, 0.95)<br>0.81 (0.63, 1.02)<br>0.68 (0.52, 0.88)<br>0.69 (0.53, 0.91)<br><br>0.89 (0.82, 0.98)	
							ADA diet	Q1: 24.1<br>Q2: 27.5<br>Q3: 30.0<br>Q4: 32.9<br>Q5: 36.7<br><br>per SD	1.00<br>0.78 (0.60, 1.02)<br>0.73 (0.58, 0.93)<br>0.71 (0.55, 0.91)<br>0.57 (0.42, 0.76)<br><br>0.82 (0.75, 0.90)	
							Paleo diet	Q1: 32.0<br>Q2: 37.1<br>Q3: 40.0<br>Q4: 43.5<br>Q5: 48.6<br><br>per SD	1.00<br>1.18 (0.92, 1.52)<br>1.22 (0.95, 1.57)<br>1.01 (0.78, 1.31)<br>1.04 (0.78, 1.39)<br><br>0.99 (0.90, 1.08)	
					Stroke	372	DASH diet	Q1: 18.9<br>Q2: 23.1<br>Q3: 25.5<br>Q4: 27.9<br>Q5: 31.7<br><br>per SD	1.00<br>0.67 (0.49, 0.93)<br>0.63 (0.45, 0.90)<br>0.89 (0.66, 1.21)<br>0.56 (0.40, 0.80)<br><br>0.88 (0.79, 0.99)	Age, race, education, income, marital status, physical activity, smoking, BMI, WHI study arm, geographical region, age at T2D diagnosis, energy intake, insulin use, blood pressure, history of high cholesterol, income x time, age at T2D onset x time, HRT, clinical trial arm x time, DASH model additionally adjusted for alcohol
							Alternate Mediterranean diet score	Q1: 1.6<br>Q2: 3.0<br>Q3: 4.0<br>Q4: 5.0<br>Q5: 6.5	1.00<br>0.88 (0.65, 1.20)<br>0.78 (0.56, 1.07)<br>0.82 (0.58, 1.14)<br>0.67 (0.47, 0.96)	

								per SD	0.87 (0.77, 0.97)	
							ADA diet	Q1: 24.1<br>Q2: 27.5<br>Q3: 30.0<br>Q4: 32.9<br>Q5: 36.7<br><br>per SD	1.00<br>0.84 (0.60, 1.19)<br>0.77 (0.56, 1.05)<br>0.85 (0.61, 1.18)<br>0.74 (0.51, 1.09)<br><br>0.89 (0.78, 1.00)	
							Paleo	Q1: 32.0<br>Q2: 37.1<br>Q3: 40.0<br>Q4: 43.5<br>Q5: 48.6<br><br>per SD	1.00<br>1.08 (0.78, 1.48)<br>1.04 (0.75, 1.44)<br>0.69 (0.49, 0.97)<br>0.84 (0.58, 1.21)<br><br>0.90 (0.79, 1.01)	
Hu,<br>2023 (20)	NHS, HPFS, USA,<br>>30 y*	10,101 men and women with T2D,<br>30 – 75 y*	FFQ	Vital records, National Death Index, reports by participants' next of kin or postal service; death certifications or review of medical records	CVD mortality	1,389	Total low-carbohydrate diet score	Q1: 9.5<br>Q2: 14.5<br>Q3: 18.0<br>Q4: 21.2<br>Q5: 25.5<br><br>Per 10 points	1.00<br>0.85 (0.72, 1.00)<br>0.77 (0.65, 0.92)<br>0.91 (0.76, 1.09)<br>0.86 (0.71, 1.04)<br><br>0.90 (0.82, 1.00)	Age, race, total energy, physical activity, alcohol, prediagnosis AHEI, smoking, BMI, multivitamin use, family history of diabetes, family history of MI, family history of cancer, diabetes duration, time interval betreen diagnosis date and latest postdiagnosis FFQ return date, postmenopausal hormone use, oral hypoglycemic drug use, insulin use
							Vegetable low-carbohydrate diet score	Q1: 10.0<br>Q2: 13.3<br>Q3: 16.0<br>Q4: 18.8<br>Q5: 22.7<br><br>Per 10 points	1.00<br>0.94 (0.80, 1.11)<br>1.03 (0.87, 1.22)<br>1.00 (0.84, 1.20)<br>0.69 (0.56, 0.85)<br><br>0.84 (0.74, 0.95)	
							Animal low-carbohydrate diet score	Q1: 8.5<br>Q2: 14.0<br>Q3: 18.0<br>Q4: 21.8<br>Q5: 26.0<br><br>Per 10 points	1.00<br>0.94 (0.79, 1.12)<br>0.94 (0.79, 1.12)<br>0.90 (0.75, 1.08)<br>0.97 (0.81, 1.18)<br><br>0.96 (0.87, 1.05)	
							Healthy low-carbohydrate diet score	Q1: 10.0<br>Q2: 13.7<br>Q3: 16.0<br>Q4: 19.0<br>Q5: 23.0<br><br>Per 10 points	1.00<br>0.98 (0.82, 1.17)<br>0.96 (0.81, 1.15)<br>0.93 (0.78, 1.12)<br>0.79 (0.64, 0.97)<br><br>0.86 (0.75, 0.97)	
							Unhealthy low-carbohydrate diet score	Q1: 8.0<br>Q2: 13.3<br>Q3: 17.0<br>Q4: 20.0<br>Q5: 24.5<br><br>Per 10 points	1.00<br>1.04 (0.88, 1.24)<br>0.92 (0.77, 1.10)<br>0.93 (0.77, 1.12)<br>1.01 (0.83, 1.23)<br><br>0.98 (0.89, 1.09)	

Huang, 2024 (21)	UK Biobank, UK, 12.1 y	11,033 men and women with T2D, 58.9 y	na	Hospital inpatient admissions	CVD	1,975	Dietary score (fruit, vegetables, processed meat, red meat, total fish, milk, spread type, cereals, salt, water)	Non-ideal<br>Ideal	1.00<br>0.88 (0.82, 0.96)	Age, sex, alcohol, annual income, antihypertensive medication, diabetes duration, education, ethnicity, family history of diabetes, family history of CVD, glucose-lowering medication, lipid-lowering medication, Townsend deprivation index, blood pressure, non-HDL-C, BMI, HbA1c, smoking, sleep time, physical activity
Ibsen, 2022 (22)	COSM, SMC, Sweden, 22 y*	3,587 men and women with diabetes, 59 y*	FFQ	Swedish Patient Register	HF	1,040	DASH	Q1<br>Q5	1.00<br>0.78 (0.62, 0.97)	Age, sex, education, smoking, alcohol, walking/cycling, corticosteroid use, aspirin use, dietary supplements use, use of HRT in women, family history of MI, total energy intake
Johansson, 2021 (23)	MDCS, Sweden, 21.2 y*	1,111 men and women with diabetes, 58 y*	Diet history method: 7-day menu book and semiquantitative FFQ	Swedish National Patient register, Cause of Death Register	Stroke	203	Diet quality index (based on Swedish nutrition recommenda-tions)	Low (0-1 points)<br>Medium (2-4 points)<br>High (5-6 points)	1.00<br>0.85 (0.52, 1.41)<br>0.80 (0.46, 1.39)	Age, sex, stroke heredity score, educational level, smoking, BMI, physical activity, alcohol
Kim, 2019 (24)	ARIC, USA, 25 y*	1,230 men and women with diabetes, 53.7 y*	FFQ	Annual telephone calls, local hospital discharge records, state death records, National Death Index	CVD	na	PDI	Q1<br>Q5	1.00<br>0.80 (0.59, 1.04)	Age, sex, race, center, total energy intake, education, smoking, physical activity, alcohol, margarine consumption, total cholesterol, lipid medication use, baseline kidney function, hypertension, BMI
							hPDI	Q1<br>Q5	1.00<br>0.76 (0.58, 1.01)	
							uPDI	Q1<br>Q5	1.00<br>1.04 (0.79, 1.39)	
							Provegetarian Diet Index	Q1<br>Q5	1.00<br>0.75 (0.58, 0.97)	
Lara, 2019 (25)	REGARDS, USA, 8.7 y*	2,381 men and women with diabetes, 64 y*	FFQ	Self reported hospitalizations, medical records	HF	108	Plant based diet (PCA derived)	Q1<br>Q2<br>Q3<br>Q4	1.00<br>1.22 (0.70, 2.14)<br>0.80 (0.44, 1.45)<br>0.63 (0.32, 1.27)	Age, sex, race, education, household income, region, total energy intake, smoking, physical activity, sodium intake, BMI, waist circumference, hypertension, dyslipidemia, artrial fibrillation, eGFR, albumin creatinine ratio
							Convenience diet (PCA derived)	Q1<br>Q2<br>Q3<br>Q4	1.00<br>0.96 (0.53, 1.74)<br>1.28 (0.70, 2.34)<br>1.15 (0.56, 2.39)	
							Alcohol and salads diet (PCA derived)	Q1<br>Q2<br>Q3<br>Q4	1.00<br>1.11 (0.65, 1.91)<br>0.89 (0.49, 1.62)<br>0.92 (0.49, 1.75)	
							Sweets diet (PCA derived)	Q1<br>Q2<br>Q3<br>Q4	1.00<br>1.03 (0.58, 1.84)<br>1.38 (0.76, 2.49)<br>0.78 (0.34, 1.80)	
							Southern diet (PCA derived)	Q1<br>Q2<br>Q3<br>Q4	1.00<br>0.97 (0.43, 2.21)<br>0.95 (0.43, 2.10)<br>1.00 (0.44, 2.26)	
Li, 2024 (26)	Kailuan Study, China, 11.5 y	19,915 men and women with T2D, 55.3 y	Questionnaire	Municipal Social Insurance Institution, Hospital Discharge Register	CVD	3,295	Diet quality (measured by intake of salt, tea, fatty foods)	Per 20 points	0.97 (0.96, 0.99)	Age, sex, education, income, marital status, alcohol, family history of diabetes, family history of CVD
					Heart disease	1,751		Per 20 points	0.97 (0.95, 0.99)	
					Stroke	1,776		Per 20 points	0.97 (0.95, 0.99)	

Liu,<br>2025 (27)	NHANES,<br>USA,<br>9.8 y*	561 men and women with diabetes and sarcopenia,<br>51.4 y*	24h dietary recalls	National Death Index	CVD mortality	na	PDI	Continuous	0.94 (0.89, 1.01)	Age, sex, race, marital status, poverty-income ratio, education, smoking, alcohol, hypertension, physical activity, history of cancer or CVD, statin use, total energy intake
Liu,<br>2024 (28)	UK Biobank,<br>UK,<br>12.7 y	15,860 men with T2D,<br>59.9 y	Short FFQ	Death registration records	CVD mortality	253	Healthy diet (fruit, vegetables, whole grains, fish, red meat, processed meat, refined grains, alcohol consumption)	Nonadherence<br>Adherence	1.00<br>0.84 (0.73, 0.98)	Age, ethnicity, Townsend deprivation index, sedentary time, hypertension, hyperlipidemia, HbA1c levels, diabetes duration, diabetes medication, BMI, smoking, physical activity, sleep duration, menopausal status, use of HRT (in women)
		9,992 women with T2D,<br>59.1 y				95		Nonadherence<br>Adherence	1.00<br>0.90 (0.70, 1.17)	
Liu,<br>2023 (29)	na,<br>China,<br>8.2 y	19,863 men and women with T2D,<br>63 y	FFQ	Jiangsu Provincial CDC and Prevention Death Surveillance System	CVD mortality	1,124	Adherence to Dietary Guidelines for Chinese Residents	0-1 food group<br>2 food groups<br>3 food groups<br>4 food groups<br>5-9 food groups<br><br>Per food group	1.00<br>0.88 (0.72, 1.08)<br>0.90 (0.74, 1.10)<br>0.75 (0.60, 0.94)<br>0.67 (0.52, 0.87)<br><br>0.91 (0.87, 0.95)	Age, sex, family income, education, BMI, smoking, alcohol, physical activity, diabetes course, abnormal blood lipids, hypertension, hypoglycemic drugs, insulin, history of CHD and stroke
Liu,<br>2023 (30)	UK Biobank,<br>UK,<br>10.8 y	22,473 men and women with diabetes,<br>58.4 y	Short FFQ	Hospital inpatient records	CVD	5,209	Healthy diet (fruits, vegetables, whole grains, fish, dairy, vegetable oils, refined grains, processed meat, unprocessed meat, SSB)	Q1<br>Q2<br>Q3<br>Q4<br>Q5	1.00<br>0.99 (0.91, 1.08)<br>0.97 (0.89, 1.06)<br>0.93 (0.85, 1.02)<br>0.86 (0.79, 0.95)	Age, sex, race, center, BMI, household income, Townsend deprivation index, smoking, alcohol consumption, physical activity, history of hypertension, history of high cholesterol, family history of cardiovascular disease, family history of diabetes, vitamin supplement use, mineral supplement use, aspirin use, diabetes duration
					CHD	3,552		Q1<br>Q2<br>Q3<br>Q4<br>Q5	1.00<br>0.95 (0.86, 1.05)<br>0.97 (0.87, 1.07)<br>0.90 (0.81, 1.00)<br>0.83 (0.75, 0.93)	
					Stroke	881		Q1<br>Q2<br>Q3<br>Q4<br>Q5	1.00<br>0.87 (0.71, 1.06)<br>0.77 (0.63, 0.95)<br>0.79 (0.64, 0.97)<br>0.71 (0.57, 0.88)	
		7,922 men and women with diabetes,<br>58.4 y	24h recalls		CVD	1,585	DASH	T1<br>T2<br>T3	1.00<br>1.03 (0.91, 1.16)<br>0.96 (0.85, 1.09)	
					CHD	1,100		T1<br>T2<br>T3	1.00<br>1.09 (0.94, 1.26)<br>0.98 (0.85, 1.14)	
					Stroke	262		T1<br>T2<br>T3	1.00<br>1.25 (0.93, 1.68)<br>1.00 (0.73, 1.37)	
		7,922 men and women with diabetes,<br>58.4 y	24h recalls		CVD	1,585	Alternate Mediterranean diet score	T1<br>T2<br>T3	1.00<br>0.87 (0.77, 0.99)<br>0.84 (0.73, 0.96)	
					CHD	1,100		T1<br>T2<br>T3	1.00<br>0.94 (0.81, 1.10)<br>0.88 (0.75, 1.04)	
					Stroke	262		T1<br>T2<br>T3	1.00<br>0.77 (0.57, 1.05)<br>0.90 (0.65, 1.24)	

Liu,<br>2018 (31)	NHS, HPFS,<br>USA<br>13.3 y	11,527 men and women with T2D,<br>62.6 y	FFQ	Medical Records, National Death Index, next of kin or postal authorities, death certificates, hospital records, autopsy records	CVD incidence	2311	AHEI	Q1: 38.8<br>Q2: 45.1<br>Q3: 50.<br>Q4: 54.9<br>Q5: 62.1	1.00<br>0.97 (0.86, 1.11)<br>0.89 (0.79, 1.02)<br>0.93 (0.82, 1.06)<br>0.84 (0.74, 0.97)	Age, sex, ethnicity, BMI at diabetes diagnosis, menopausal status, family history of diabetes, family history of MI, current aspirin use, current multivitamin use, diabetes duration, physical activity, cigarette smoking, alcohol consumption
					CHD incidence	1844		Q1: 38.8<br>Q2: 45.1<br>Q3: 50.<br>Q4: 54.9<br>Q5: 62.1	1.00<br>1.02 (0.89, 1.18)<br>0.91 (0.79, 1.05)<br>0.96 (0.83, 1.10)<br>0.87 (0.75, 1.02)	
					Stroke incidence	498		Q1: 38.8<br>Q2: 45.1<br>Q3: 50.<br>Q4: 54.9<br>Q5: 62.1	1.00<br>0.87 (0.66, 1.13)<br>0.83 (0.63, 1.09)<br>0.88 (0.67, 1.15)<br>0.78 (0.59, 1.04)	
					CVD mortality	858		Q1: 38.8<br>Q2: 45.1<br>Q3: 50.<br>Q4: 54.9<br>Q5: 62.1	1.00<br>0.97 (0.79, 1.19)<br>0.90 (0.74, 1.11)<br>0.84 (0.68, 1.04)<br>0.77 (0.62, 0.97)	
Mita,<br>2025 (32)	na (participants from outpatient clinic),<br>Japan,<br>7.5 y	731 men and women with T2D,<br>57.8 y	Brief DHQ	Clinical assessment	CVD incidence	50	Total low-carbohydrate diet score	Q1: ≤ 10<br>Q2: 10-15<br>Q3: 15-20<br>Q4: >20<br><br>Per point	1.00<br>0.61 (0.27, 1.39)<br>1.14 (0.56, 2.31)<br>0.16 (0.04, 0.71)<br><br>0.95 (0.91, 1.00)	Age, gender, total energy intake, HDL-C, triglycerides, uric acid, urinary albumin excretion, use of antiplatelet agents
							Animal low-carbohydrate diet score	Q1: ≤ 9.5<br>Q2: 9.5-15<br>Q3: 15-20<br>Q4: >20<br><br>Per point	1.00<br>1.10 (0.49, 2.44)<br>1.21 (0.56, 2.63)<br>0.30 (0.08, 1.08)<br><br>0.96 (0.92, 1.01)	
							Plant low-carbohydrate diet score	Per point	0.95 (0.89, 1.01)	
Mokhtari,<br>2019 (33)	GCS,<br>Iran,<br>10.6 y*	3,371 men and women with diabetes,<br>40-87 y*	FFQ	Annual follow-up, confirmed by physician visit and verbal autopsy questionnaire	CVD mortality	465	DASH score	9 - 20<br>21 - 25<br>26 - 30<br>31 - 39	1.00<br>0.89 (0.60, 1.32)<br>0.92 (0.62, 1.35)<br>0.97 (0.63, 1.49)	Age, sex, energy intake, BMI, smoking, opium use, wealth score, physical activity, history of hypertension
Murai,<br>2024 (34)	na (participants from health insurance),<br>Japan,<br>5.2 y*	5,892 men and women with diabetes,<br>52 y	Questionnaires	Medical records, prescribed medications, medical procedures, and questionnaires	CVD	163	Snacking habits after dinner	Non-ideal (≥3 times/wk)<br>Ideal (< 3 times/wk)	1.00<br>1.31 (0.79, 2.18)	Age, sex, antihypertensive drug use, cholesterol-lowering drug use
							Breakfast skipping	Non-ideal (≥3 times/wk)<br>Ideal (<3 times/wk)	1.00<br>0.95 (0.62, 1.46)	
							Eating habits (ideal breakfast and snacking habits, and eating dinner within 2 h before bedtime < 3 times/wk)	Non-ideal<br>Ideal	1.00<br>1.22 (0.89, 1.67)	
Nilsson,<br>2012 (35)	VIP,<br>Sweden,		FFQ		CVD mortality	Men:<br>53		Low (2-8 points)<br>High (14-20 points)	1.00<br>0.30 (0.14, 0.68)	

	10 y*	2,141 men and women with diabetes,<br>49 y*		Swedish national cause-of-death registry			Low-carbohydrate, high-protein score	per point	0.90 (0.84, 0.96)	Age, BMI, sedentary lifestyle, education, smoking, energy intake, alcohol, SFA
						Women:<br>17		Low (2-8 points)<br>High (14-20 points)<br><br>per point	1.00<br>5.30 (1.02, 27.70)<br><br>1.13 (0.98, 1.29)	
Pierucci,<br>2012 (36)	ONCONUT,<br>Italy,<br>5 y	87 men and women with diabetes,<br>67.1 y	FFQ	Self-report, confirmed by family physicians, hospital records	MI	29	Glycemic load	T1<br>T2<br>T3	1.00<br>0.71 (0.22, 2.33)<br>1.87 (0.61, 5.71)	Age, sex, BMI, smoking, hypertension
							Glycemic index	T1<br>T2<br>T3	1.00<br>2.11 (0.62, 7.11)<br>3.38 (1.00, 11.39)	
Rui,<br>2025 (37)	PEACE-Shanxi,<br>China,<br>2.4 y*	2,643 men and women with diabetes,<br>57.2 y*	FFQ	Self-reports	CVD incidence	na	PDI	T1: 28-46<br>T2: 47-49<br>T3: 50-60	1.00<br>1.22 (0.92, 1.64)<br>1.15 (0.83, 1.59)	Age, sex smoking, alcohol, physical activity, marital status, geographic region, education, BMI, household income, hypertension, dyslipidemia
Sattler,<br>2025 (38)	ARIC,<br>USA,<br>15 y	806 men and women with diabetes,<br>55.4 y	FFQ	Hospital records, neuroimaging reports, hospital discharges	CVD	393	Change in diet score	non-ideal to non-ideal<br>Non-ideal to ideal<br>Ideal to non-ideal<br>Ideal to ideal	1.00<br>1.21 (0.80, 1.84)<br>1.10 (0.70, 1.74)<br>0.49 (0.16, 1.53)	Age, sex, ethnicity, education, baseline total Life's Simple 7 score
Sijtsma,<br>2015 (39)	Alpha Omega Trial,<br>Netherlands,<br>6.5 y*	Na, >603 men and women with MI and diabetes,<br>60-80 y*	FFQ	Municipal registries, Dutch National Mortality Registry	CVD mortality	na	Dutch Healthy Nutrient and Food Score	< median<br>> median	1.00<br>0.87 (0.56, 1.39)	Age, sex, energy intake, alcohol intake, education, physical activity, smoking status
Song,<br>2023 (40)	NHANES,<br>USA,<br>10.0 y*	1,021 men and women with T2D,<br>47.1 y*	FFQ	National Death Index	CVD mortality	130	MIND diet score	Low score (≤ 8)<br>High score (>8)	1.00<br>0.50 (0.29, 0.87)	Age, sex, ethnicity, education level, family income to poverty ratio, smoking status, BMI, physical activity, hypertension, dyslipidemia, energy intake, eGFR
Sotos-Prieto,<br>2024 (41)	UK Biobank,<br>UK,<br>9.4 y*	3,513 men and women with diabetes,<br>58.8 y*	24h dietary recalls	Hospital Episode Statistics, National Death Index	Total CVD	389	EAT-Lancet planetary Health Diet Index	Per 20 points	0.83 (0.70, 0.98)	Age, sex, ethnicity, education, deprivation index, region of assessment, smoking status, energy intake, alcohol intake, physical activity, BMI, cancer, hypertension, cholesterol-lowering medication, number of medications, vitamin supplement use
		3,594 men and women with diabetes,<br>58.8 y*			MI	308		Per 20 points	0.83 (0.68, 1.00)	
		3,804 men and women with diabetes,<br>58.8 y*			Stroke	98		Per 20 points	0.85 (0.61, 1.18)	
Su,<br>2024 (42)	Comprehensive Research on the Prevention and Control of the Diabetes,<br>China,<br>8 y	15,191 men and women with diabetes,<br>62 y	Short FFQ	Local disease records and death certificates	Stroke	706	Healthy diet score (fruits, vegetables, eggs, grains, fish, red meat, beans, milk)	Nonadherence<br>Adherence	1.00<br>0.91 (0.83, 1.00)	Age, sex, education, marital status, family history of stroke, family history of MI, BMI, duration of diabetes, diabetes medication use, smoking, alcohol intake, physical activity, sleep hours
					Ischaemic stroke	602		Nonadherence<br>Adherence	1.00<br>0.90 (0.81, 0.99)	
					Intracerebral haemorrhage	42		Nonadherence<br>Adherence	1.00<br>0.87 (0.60, 1.25)	
	China Kadoorie Biobank,	26,123 men and women			Stroke	1,414		Nonadherence<br>Adherence	1.00<br>0.83 (0.78, 0.90)	

	China,<br>9.1 y	with diabetes,<br>57.4 y			Ischaemic stroke	1,306		Nonadherence<br>Adherence	1.00<br>0.83 (0.77, 0.90)	
					Intracerebral haemorrhage	135		Nonadherence<br>Adherence	1.00<br>0.69 (0.56, 0.85)	
Sun,<br>2022 (43)	GBCS,<br>China,<br>14.8 y*	2,790 men and women with diabetes,<br>64.1 y	FFQ	Record linkage with the Death Registry	CVD mortality	411	Low-carbohydrate diet score	Q1: 6<br>Q2: 13<br>Q3: 18<br>Q4: 24	1.00<br>0.81 (0.60, 1.09)<br>0.94 (0.70, 1.27)<br>1.15 (0.86, 1.52)	Age, sex, education, family income, smoking, drinking, physical activity, BMI, history of cancer and CVD, SBP, FBG, total cholesterol, self-rated health at baseline
							Vegetable-based low-carbohydrate score	Q1: 11<br>Q2: 14<br>Q3: 17<br>Q4: 20	1.00<br>1.21 (0.89, 1.63)<br>1.59 (1.18, 2.15)<br>1.54 (1.11, 2.13)	
							Meat-based low-carbohydrate score	Q1: 6<br>Q2: 13<br>Q3: 19<br>Q4: 24	1.00<br>0.79 (0.59, 1.05)<br>0.94 (0.72, 1.24)<br>0.94 (0.70, 1.24)	
Tao,<br>2024 (44)	NHANES,<br>USA,<br>7.2 y	4,621 men and women with diabetes,<br>56.8-63.6 y	24-h dietary recall interviews	National Center for Health Statistics Public-Use Linked Mortality Files, National Death index	CVD mortality	329	hPDI	Q1: <42<br>Q2<br>Q3<br>Q4: ≥51<br><br>Per 10 point increase	1.00<br>1.08 (0.78, 1.50)<br>0.94 (0.69, 1.30)<br>0.61 (0.43, 0.87)<br><br>0.76 (0.63, 0.91)	Age, sex, ethnicity, education, marital status, PIR, BMI, smoking, drinking status, total energy intake, physical activity, use of antidiabetic medications, hyperlipidemia, hypertension, CVD, cancer
							uPDI	Q1: <41<br>Q2<br>Q3<br>Q4: ≥50<br><br>Per 10 point increase	1.00<br>1.29 (0.92, 1.81)<br>1.44 (1.03, 2.02)<br>1.71 (1.22, 2.38)<br><br>1.31 (1.10, 1.57)	
Tektonidis,<br>2016 (45)	COSM,<br>Sweden,<br>10.9 y*	2,229 men with diabetes,<br>59.1 y*	FFQ	Linkage to Swedish National Patient and the Swedish Cause of Death Registers	Heart failure	181	Modified Mediterranean diet score	Low score: 0-3<br>High score: 6-8	1.00<br>0.91 (0.55, 1.48)	Age, education, family history of MI, smoking, walking or/and cycling, exercise, BMI, history of hypertension, history of hypercholesterolaemia, aspirin use, total energy intake
Wang,<br>2024 (46)	EPIC-Norfolk,<br>UK,<br>21.4 y*	1,662 men and women with T2D,<br>58.6 y*	FFQ	Hospital admission records, death certificates	Total CVD	353	Mediterranean diet score (median-based, 0-9 points)	Q1: 0-3<br>Q2: 4<br>Q3: 5<br>Q4: 6-9<br><br>per SD	1.00<br>1.06 (0.81, 1.39)<br>0.85 (0.62, 1.17)<br>0.77 (0.57, 1.04)<br><br>0.93 (0.84, 1.04)	Age, sex, BMI, smoking status, physical activity, social class, marital status, education level, medication use (anti-hypertensive drugs or lipid-lowering drugs), family history of MI, stroke, or diabetes
							Mediterranean diet score (based on Mediterranean Diet Foundation, 0-15 points)	Q1: 0-7.55<br>Q2: 7.56-8.45<br>Q3: 8.46-9.34<br>Q4: 9.35-15<br><br>per SD	1.00<br>1.08 (0.80, 1.39)<br>0.92 (0.68, 1.25)<br>0.89 (0.65, 1.23)<br><br>0.95 (0.84, 1.06)	
Wang,<br>2022 (47)	NHANES,<br>USA,<br>6.3 y	4,415 men and women with diabetes,<br>58.3 y	24-h dietary recall	National Death Index	CVD mortality	na	DASH score	≤2<br>>2	1.00<br>0.88 (0.57, 1.37)	Age, sex, race, BMI, history of hypertension, smoking, total cholesterol, CKD, energy intake

Wang,<br>2022 (48)	NHANES,<br>USA,<br>5.9 y	4,699 men and women with diabetes,<br>58.8 y	24-h dietary recalls	National death index	CVD mortality	215	Dietary Antioxidant Quality Score	0-2<br>3-4<br>5-6	1.00<br>0.75 (0.50, 1.13)<br>0.56 (0.35, 0.90)	Age, sex, ethnicity, BMI, smoking, alcohol, education, income level, exercise, total energy intake, dietary supplements use, self-reported hypertension, dyslipidemia, heart disease, cancer, family history of diabetes, diabetes medication, duration of diabetes, HbA1c
							Dietary Antioxidant Index	T1<br>T2<br>T3	1.00<br>0.74 (0.51, 1.06)<br>0.51 (0.31, 0.82)	
Weston,<br>2022 (49)	JHS,<br>USA,<br>13 y*	Na, men and women with diabetes,<br>54.2 y*	FFQ	Annual telephone interviews, surveillance of hospitalizations and death certificates	CVD incidence	na	PDI	T1: 48<br>T3: 61	1.00<br>1.04 (1.00, 1.09)	Age, sex, total energy intake, education, smoking, alcohol, margarine intake, physical activity, BMI, total cholesterol, hypertension, eGFR, HRT use, statin use
							hPDI	T1: 48<br>T3: 60	1.00<br>1.01 (0.96, 1.05)	
							uPDI	T1: 48<br>T3: 61	1.00<br>1.00 (0.96, 1.04)	
Xie,<br>2023 (50)	NHANES,<br>USA,<br>7.1 y	4,924 men and women with diabetes,<br>57.8 y	24-h dietary recalls	National Death Index	CVD mortality	272	Eating frequency	<3<br>3<br>4<br>>4<br><br>per 1 time increment	1.00<br>0.89 (0.49, 1.60)<br>0.54 (0.30, 0.97)<br>0.53 (0.26, 1.08)<br><br>0.77 (0.63, 0.93)	Age, sex, ethnicity, BMI, education, family income-poverty ratio, alcohol, smoking, physical activity, HEI score, energy intake, breakfast skipping, diet record days, diabetes duration, diabetes medication, hypertension, hypercholesterolemia, CVD, hypertension and hypercholesterolemia medication use, HbA1c, HOMA2_IR, SBP, DBP, total cholesterol, triglycerides, HDL-C, LDL-C, eGFR
Youqi,<br>2025 (51)	NHANES,<br>USA,<br>9.3 y	5,875 men and women with diabetes,<br>Na	24h dietary recalls	National Death Index	CVD mortality	486	HEI	Men<br>Q1<br>Q2<br>Q3<br>Q4<br><br>Women<br>Q1<br>Q2<br>Q3<br>Q4	 1.00<br>1.05 (0.69, 1.61)<br>0.80 (0.52, 1.23)<br>0.46 (0.30, 0.70)<br><br><br>1.00<br>1.02 (0.62, 1.70)<br>1.02 (0.65, 1.59)<br>1.03 (0.60, 1.75)	Age, ethnicity, education, alcohol, smoking, exercise level, baseline hypertension, baseline CVD, HbA1c, BMI, cholesterol, blood glucose, triglycerides, triglyceride-glucose index, BMI-adjusted triglyceride-glucose index
							AHEI	Men<br>Q1<br>Q2<br>Q3<br>Q4<br><br>Women<br>Q1<br>Q2<br>Q3<br>Q4	 1.00<br>0.92 (0.63, 1.34)<br>0.56 (0.36, 0.86)<br>0.39 (0.25, 0.62)<br><br><br>1.00<br>1.04 (0.64, 1.71)<br>0.58 (0.35, 0.95)<br>0.86 (0.52, 1.40)	
							Alternate Mediterranean diet score	Men<br>Q1<br>Q2<br>Q3<br>Q4	 1.00<br>0.90 (0.56, 1.46)<br>0.63 (0.36, 1.11)<br>0.58 (0.36, 0.95)	

								Women<br>Q1<br>Q2<br>Q3<br>Q4	 1.00<br>0.65 (0.40–1.08)<br>0.63 (0.32–1.27)<br>0.71 (0.36–1.40)	
Yu,<br>2025 (52)	CRPCD,<br>China,<br>9 y	13,776 men and women with T2D,<br>62 y	Short FFQ	Provincial death registration system	CVD mortality	751	Chinese Dietary Guideline	< 4 recommended food categories<br>≥ 4 recommended food categories	1.00<br><br>0.74 (0.62, 0.88)	Age, sex, education, household income, BMI, waist circumference, hypertension, dyslipidemia, diabetes duration, antidiabetic medication, insulin use, alcohol, smoking, physical activity, sleep duration, sedentary behavior
Yuan,<br>2023 (53)	NHANES,<br>USA,<br>8.6 y*	1,804 men and women with undiagnosed diabetes,<br>57.9 y	24h dietary recall	National Death Index	Heart disease mortality	na	HEI-2015	Per SD	0.87 (0.68,1.12)	Age, sex, educational level, BMI, smoke, hypertension, hyperlipidemia, diabetes diagnosis, alcohol consumption
					Cerebrovascular disease mortality			Per SD	0.82 (0.55, 1.24)	
		4,101 men and women with diagnosed diabetes,<br>60.4 y			Heart disease mortality			Per SD	0.94 (0.76,1.15)	
					Cerebrovascular disease mortality			Per SD	0.78 (0.58, 1.05)	
Zhang,<br>2024 (54)	MDC, MDC-CC,<br>Sweden,<br>25 y*	984 men and women with diabetes,<br>57.8 y*	Combined method of 7-day food diary, FFQ, and a diet interview	Swedish Hospital Discharge Register	HF	190	EAT Lancet Planetary Health Diet index	Per 3 points	0.95 (0.83, 1.09)	Age, sex, dietary assessment version, season, total energy intake, physical activity, alcohol, smoking, education, BMI, hypertension, use of lipid-lowering drugs, family history of MI
Zhang,<br>2023 (55)	UK Biobank,<br>UK,<br>na	19,181 men and women with diabetes,<br>59.7 y	24-h dietary recalls	National Health Service Digital and the National Health Service Central Register	CVD incidence	3,309	Diet (dietary recommendations for cardiovascular health)	Unhealthy diet<br>Healthy diet (meeting ≥5 items)	1.00<br>0.93 (0.86, 1.00)	Age, sex, race, education, household income, employment status, hypertension, cancer, years since diabetes diagnoses, uses of glucose-lowering, antihypertensive, and lipid-lowering medications, family history of diabetes, CVD, and cancer, FBG/HbA1c, total cholesterol, smoking, alcohol, physical activity, body weight
	DFTJ,<br>China,<br>na	5,788 men and women with diabetes, 65.3 y*	Short FFQ	Dongfeng Motor Corporation's healthcare service system		1,704	Diet (vegetables, fruit, meat)	Unhealthy diet<br>Healthy diet (daily vegetables/fruit, no daily meat intake)	1.00<br>0.96 (0.86, 1.07)	Age, sex, marital status, education, hypertension, cancer, years since diabetes diagnoses, uses of glucose-lowering, antihypertensive, and lipid-lowering medications, family history of diabetes, CVD, and cancer, FBG/ HbA1c, total cholesterol, smoking, alcohol, physical activity, body weight
Zhang,<br>2023 (56)	NHANES,<br>USA,<br>5.6 y	5,676 men and women with diabetes,<br>59.4 y	24h dietary recall	National Death Index	CVD mortality	291	Composite dietary antioxidant index	Q1: <-2.60<br>Q2: -2.60 to -0.74<br>Q3: -0.74 to 1.79<br>Q4: ≥1.79	1.00<br>0.72 (0.44, 1.18)<br>1.13 (0.69, 1.85)<br>0.74 (0.49, 1.12)	Age, sex, ethnicity, BMI, education level, family income-poverty ratio, smoking status, physical activity, HbA1c, hypertension, hyperuricemia
Zhuang,	UK Biobank,					1,369	PDI	T1: 33-50	1.00	

2024 (57)	UK,<br>9.6 y	7,798 men and women with diabetes,<br>58.1 y	24h dietary recalls	Hospital inpatient records, hospital admission records	CVD incidence			T2: 51-55<br>T3: 56-72	0.98 (0.86, 1.11)<br>0.96 (0.85, 1.09)	Age, sex, race, centers, BMI, household income, Townsend deprivation index, smoking, alcohol, physical activity, history of hypertension, high cholesterol at baseline, family history of CVD, family history of diabetes, vitamin supplementation, mineral supplementation, aspirin use, diabetes duration
							hPDI	T1: 32-51<br>T2: 52-56<br>T3: 57-76	1.00<br>0.94 (0.83, 1.07)<br>1.00 (0.89, 1.14)	
							uPDI	T1: 31-51<br>T2: 52-56<br>T3: 57-75	1.00<br>1.02 (0.90, 1.16)<br>1.14 (1.00, 1.29)	
					CHD incidence	994	PDI	T1: 33-50<br>T2: 51-55<br>T3: 56-72	1.00<br>1.04 (0.89, 1.21)<br>0.94 (0.80, 1.10)	
							hPDI	T1: 32-51<br>T2: 52-56<br>T3: 57-76	1.00<br>0.98 (0.84, 1.14)<br>1.02 (0.87, 1.18)	
							uPDI	T1: 31-51<br>T2: 52-56<br>T3: 57-75	1.00<br>1.11 (0.95, 1.30)<br>1.13 (0.97, 1.32)	
					Stroke incidence	242	PDI	T1: 33-50<br>T2: 51-55<br>T3: 56-72	1.00<br>0.75 (0.55, 1.02)<br>0.83 (0.61, 1.13)	
							hPDI	T1: 32-51<br>T2: 52-56<br>T3: 57-76	1.00<br>0.81 (0.58, 1.11)<br>0.93 (0.69, 1.26)	
							uPDI	T1: 31-51<br>T2: 52-56<br>T3: 57-75	1.00<br>0.85 (0.61, 1.18)<br>1.18 (0.87, 1.59)	
Zhu,<br>2024 (58)	UK Biobank,<br>UK,<br>13.5 y	14,543 men and women with T2D,	na	Hospital inpatient admissions and death registries	Peripheral artery disease	628	Dietary score (fruit, vegetable, whole grains, fish/shellfish, dairy, vegetable oils, refined grains, processed meats, unprocessed meats, SSB)	< 5 ideal components<br>≥ 5 ideal components	1.00<br>0.92 (0.75, 1.13)	Age, sex, ethnicity, education, Townsend Deprivation Index, smoking, physical activity, alcohol, waist-to-hip ratio, sleep duration, family history of CVD, hypertension, antihypertensive medication, lipid lowing medication, aspirin, diabetes duration, HbA1c, diabetes medication

AOT, Alpha Omega Trial; ARIC, Atherosclerosis Risk in Communities; BMI, body mass index; CAD, Coronary Artery Disease; CHD, coronary heart disease; CHS, Cardiovascular Health Study; CKD, chronic kidney diseas; COSM, Cohort of Swedish Men; CRPCD, Comprehensive Research on the Prevention and Control of Diabetes; CVD, cardiovascular disease; DASH, dietary approach to stop hypertension; DBP, diastolic blood pressure; DCS, Hoorn Diabetes Care System; DFTJ, Dongfeng-Tongji cohort; DHQ, diet history questionnaire; eGFR, estimated glomerular filtration rate; FA, fatty acids; FBG, fasting blood glucose; FDS1, Fremantle Diabetes Study Phase I; FFQ, food frequency questionnaire; GBCS, Guangzhou Biobank Cohort Study; HDL-C, high-density lipoprotein cholesterol; HF, heart failure; hPDI, healthful plant-based diet index; HPFS, Health Professionals Follow-up Study; HRT, hormone replacement therapy; JDCS, Japan Diabetes Complications Study; JHS, Jackson Heart Study; JPHC, Japan Public Health Center–Based Prospective Study; KoGES, Korean genome and epidemiology study; LDL-C, low-density lipoprotein cholesterol; MDC, Malmö Diet and Cancer study; MDC-CC, Malmö Diet and Cancer Cardiovascular Cohort; MESA, Multi-Ethnic Study of Atherosclerosis; MI, myocardial infarction; MUFA, monounsaturated fatty acids; NHANES, National Health and Nutrition Examination Survey; NHS, Nurses' Health Study; NPPCD, National Program for Prevention and Control of Diabetes; PEACE, China Patient-centered Evaluative Assessment of Cardiac Events; PHS, Physicians' Health Study; PREDIMED, Prevención con Dieta Mediterránea; PUFA, polyunsaturated fatty acids; REGARDS, Reasons for Geographic and Racial Differences in Stroke; SBP, systolic blood pressure; SFA, saturated fatty acids; SSB, sugar-sweetened beverages; T2D, type 2 diabetes; uPDI, unhealthful plant-based diet index; WHI, Women's Health Initiative; WHS, Women's Health Study;

* data for entire study population, no data for participants with diabetes provided

Table S6. Risk of bias of included studies using the Risk Of Bias In Non-randomized Studies - of Exposure (ROBINS-E) tool.

Reference	D1	D2	D3	D4	D5	D6	D7	Overall
Bonaccio 2016 (2)	-	-	-	+	+	+	+	-
Burger 2012 (3)	-	-	-	+	+	+	+	-
Chen 2025 (4)	-	X	-	+	-	+	+	X
Dai 2024 (5)	-	-	-	+	-	+	+	-
Damigou 2025 (Eat Lancet) (6)	X							X
Damigou 2025 (MDS) (7)	X							X
Dehghan 2012 (8)	X							X
Del Gobbo 2015 (9)	X							X
Deng 2017 (10)	X							X
Fan 2024 (11)	X							X
Gamba 2023 (12)	X							X
Gao 2023 (13)	X							X
Ghaemi 2021 (14)	X							X
Han 2021 (15)	X							X
Hardy 2010 (16)	X							X
Harriss 2007 (17)	X							X
He 2023 (18)	X							X
Hirahatake 2019 (19)	-	-	-	+	+	-	+	-
Hu 2023 (20)	-	-	+	+	+	+	-	-
Huang 2024 (21)	X							X
Ibsen 2022 (22)	X							X
Johansson 2021 (23)	X							X
Kim 2019 (24)	X							X
Lara 2019 (25)	X							X
Li 2024 (26)	X							X
Liu 2025 (27)	X							X
Liu 2024 (28)	X							X
Liu 2023 (China) (29)	X							X
Liu 2023 (UK Biobank) (30)	-	-	-	+	-	+	+	-
Liu 2018 (31)	X							X
Mita 2025 (32)	X							X
Mokhtari 2019 (33)	X							X
Murai 2024 (34)	X							X
Nilsson 2012 (35)	X							X
Pierucci 2012 (36)	X							X
Rui 2025 (37)	X							X
Sattler 2025 (38)	X							X
Sijtsma 2015 (39)	X							X
Song 2023 (40)	X							X
Sotos-Prieto 2024 (41)	X							X
Su 2024 (42)	X							X
Sun 2022 (43)	X							X
Tao 2024 (44)	-	-	-	+	-	+	+	-
Tektonidis 20216 (45)	X							X
Wang 2024 (46)	X							X
Wang 2022 (DASH) (47)	X							X
Wang 2022 (DAI) (48)	-	-	-	+	+	+	+	-
Weston 2022 (49)	X							X

Xie 2023 (50)	-	-	-	+	+	+	+	-
Youqi 2025 (51)	X							X
Yu 2025 (52)	X							X
Yuan 2023 (53)	X							X
Zhang 2024 (MDC) (54)	X							X
Zhang 2023 (UK Biobank,DFTJ) (55)	X							X
Zhang 2023 (NHANES) (56)	X							X
Zhuang 2024 (57)	X							X
Zhu 2024 (58)	X							X

DAI, Dietary Antioxidant Indices; DASH, Dietary Approaches to Stop Hypertension; DFTJ, Dongfeng-Tongji cohort; MDC, Malmö Diet and Cancer study; MDS, Mediterranean diet score; NHANES, National Health and Nutrition Examination Survey.

Domains:

D1: Risk of bias due to confounding

D2: Risk of bias arising from measurement of the exposure

D3: Risk of bias in selection of participants into the study/analysis

D4: Risk of bias due to post-exposure interventions

D5: Risk of bias due to missing data

D6: Risk of bias arising from measurement of the outcome

D7: Risk of bias in selection of the reported results

Judgement

+ Low

- Some concerns

X High

Table S7. Certainty of evidence for associations of dietary factors and cardiovascular diseases in individuals with type 2 diabetes.

Certainty assessment							№ of participants		Effect		Certainty
№ of studies	Study design	Risk of bias	Inconsistency	Indirectness	Imprecision	Other considerations	Cases	Total	Relative (95% CI)	Absolute (95% CI)	
**Mediterranean diet (per 1 point)**											
6	prospective observational studies	serious[a]	not serious	not serious	not serious	dose-response gradient*	4259	23708	**HR 0.95** (0.93 to 0.97)	**8 fewer per 1.000** (from 11 fewer to 5 fewer)	⊕⊕⊕◯ Moderate[a]
**Dietary Approaches to Stop Hypertension (per 5 points)**											
3	prospective observational studies	serious[a]	not serious	not serious	serious[b]	none	3504	17102	**HR 0.96** (0.89 to 1.03)	**7 fewer per 1.000** (from 20 fewer to 5 more)	⊕⊕◯◯ Low[a,b]
**Total plant-based dietary index (high vs low adherence)**											
4	prospective observational studies	very serious[c]	not serious	not serious	very serious[d]	none	>1369**	12353	**HR 1.00** (0.92 to 1.09)	**0 fewer per 1.000** (from 10 fewer to 12 more)	⊕◯◯◯ Very low[c,d]
**Healthy plant-based dietary index (per 5 points)**											
2	prospective observational studies	serious[a]	serious[e]	not serious	very serious[d]	none	1698	12419	**HR 0.94** (0.82 to 1.07)	**8 fewer per 1.000** (from 23 fewer to 9 more)	⊕◯◯◯ Very low[a,d,e]
**Unhealthy plant-based dietary index (per 5 points)**											
2	prospective observational studies	serious[a]	not serious	not serious	serious[b]	none	1698	12419	**HR 1.07** (0.97 to 1.18)	**9 more per 1.000** (from 4 fewer to 23 more)	⊕⊕◯◯ Low[a,b]
**Eat Lancet diet (per 3 points)**											
3	prospective observational studies	very serious[c]	serious[e]	not serious	not serious	dose-response gradient*	589	4723	**HR 0.65** (0.45 to 0.95)	**42 fewer per 1.000** (from 67 fewer to 6 fewer)	⊕◯◯◯ Very low[c,e]
**(Alternate) Healthy Eating Index (high vs low adherence)**											
4	prospective observational studies	very serious[c]	not serious	not serious	not serious	none	>2797**	>30271**	**HR 0.78** (0.71 to 0.86)	**33 fewer per 1.000** (from 44 fewer to 21 fewer)	⊕⊕◯◯ Low[c]
**Chinese Dietary Guideline (high vs low adherence)**											
3	prospective observational studies	very serious[c]	not serious	not serious	not serious	none	3289	59762	**HR 0.78** (0.69 to 0.87)	**12 fewer per 1.000** (from 17 fewer to 7 fewer)	⊕⊕◯◯ Low[c]
**European Dietary Guidelines (high vs low adherence)**											
2	prospective observational studies	very serious[c]	not serious	serious[f]	very serious[d]	none	>195**	>1714**	**HR 0.84** (0.59 to 1.19)	**23 fewer per 1.000** (from 59 fewer to 26 more)	⊕◯◯◯ Very low[c,d,f]

Certainty assessment							№ of participants		Effect		Certainty
№ of studies	Study design	Risk of bias	Inconsistency	Indirectness	Imprecision	Other considerations	Cases	Total	Relative (95% CI)	Absolute (95% CI)	
**Overal adherence to national dietary guidelines (high vs low adherence)**											
9	prospective observational studies	very serious[c]	not serious	not serious	not serious	none	>7735**	>84687**	**HR 0.80** (0.76 to 0.84)	**18 fewer per 1.000** (from 21 fewer to 14 fewer)	⊕⊕◯◯ Low[c]
**Glycemic load (per 5 units)**											
3	prospective observational studies	serious[a]	not serious	not serious	not serious	none	867	10481	**HR 1.00** (0.98 to 1.02)	**0 fewer per 1.000** (from 2 fewer to 2 more)	⊕⊕⊕◯ Moderate[a]
**Glycemic index (per 5 units)**											
2	prospective observational studies	serious[a]	not serious	not serious	very serious[d]	none	677	7570	**HR 1.0** (0.9 to 1.1)	**0 fewer per 1.000** (from 9 fewer to 8 more)	⊕◯◯◯ Very low[a,d]
**Low carbohydrate diet (high vs low adherence)**											
4	prospective observational studies	serious[a]	not serious	not serious	very serious[d]	none	1920	15763	**HR 0.76** (0.51 to 1.14)	**28 fewer per 1.000** (from 58 fewer to 16 more)	⊕◯◯◯ Very low[a,d]
**Vegetable-based low-carbohydrate diet (per 5 points)**											
3	prospective observational studies	serious[a]	serious[g]	not serious	very serious[d]	none	1850	13622	**HR 0.98** (0.75 to 1.26)	**3 fewer per 1.000** (from 32 fewer to 32 more)	⊕◯◯◯ Very low[a,d,g]
**Meat-based low-carbohydrate diet (per 5 points)**											
3	prospective observational studies	serious[a]	not serious	not serious	serious[b]	none	1850	13622	**HR 0.98** (0.93 to 1.03)	**3 fewer per 1.000** (from 9 fewer to 4 more)	⊕⊕◯◯ Low[a,b]
**Dietary inflammatory index (high vs low adherence)**											
3	prospective observational studies	very serious[c]	not serious	not serious	serious[b]	none	>563**	15861	**HR 1.28** (0.97 to 1.68)	**16 more per 1.000** (from 2 fewer to 40 more)	⊕◯◯◯ Very low[b,c]

CI: confidence interval; SRR: summary risk ratio.

Explanations

a. Downgraded by 1 level due to risk of bias: less than 2/3 of studies (or corresponding weight) rated with high risk of bias.
b. Downgraded by 1 level due to imprecision: 95% CI crosses threshold of a minimal important difference (absolute risk: 5 fewer or 5 more events per 1.000).
c. Downgraded by 2 levels due to risk of bias: more than 2/3 of studies (or corresponding weight) rated with high risk of bias.
d. Downgraded by 2 levels due to imprecision: 95% CI crosses threshold of both minimal important difference (absolute risk: 5 fewer and 5 more events per 1.000).
e. Downgraded by one level due to inconsistency: no overlap of 95% CIs.
f. Downgraded by one level due to indirectness: exposure definition and outcome varies between studies.
g. Downgraded by one level due to inconsistency: effect estimate of at least one study points in opposite direction.
* Although we observed a dose-response gradient, we did not upgrade for dose-response since this would results in a "high" certainty rating, with a simultaneous serious risk of bias within the primary studies due to confounding and selction bias
** Number of cases/participants unknown for at least one study

Figure S1. Forest plots for the association between the Mediterranean diet and cardiovascular diseases. A) high vs low adherence, B) linear-dose-response (per 1 point) and C) non-linear dose-response meta-analysis.

A)

Reference	Study	Comparison	N total	N cases	HR (95% CI)
Youqi 2025 (51)	NHANES	Q4 vs Q1	5875	486	0.62 (0.42, 0.92)
Wang 2024 (46)	EPIC-Norfolk	Q4 vs Q1	1662	353	0.77 (0.57, 1.04)
Liu 2023 (31)	UK Biobank	T3 vs T1	7922	1585	0.84 (0.73, 0.96)
Ghaemi 2021 (15)	NPPCD	≥8 vs ≤7 points	20338	na	0.61 (0.49, 0.76)
Hirahatake 2019 (20)	WHI	6.5 vs 1.6	5809	1454	0.77 (0.64, 0.92)
Bonaccio 2016 (2)	MOLI-SANI	High vs. poor MDS score	1995	51	0.43 (0.19, 0.98)
Tektonidis 2016 (45)	COSM	≥6 vs ≤3	2229	181	0.91 (0.55, 1.49)
Harriss 2007 (18)	MCCS	Q4 vs Q1	1524	na	0.21 (0.09, 0.48)
Overall, DL (I^2 = 62.0%, p = 0.010)					0.69 (0.58, 0.83)

.2 .5 1 2

B)

Reference	Study	Outcome	N total	N cases	HR (95% CI)
Youqi 2025 (51)	NHANES	CVD mortality	5875	486	0.91 (0.85, 0.98)
Damigou 2024 (7)	ATTICA	CVD incidence	445	330	0.33 (0.03, 3.86)
Wang 2024 (46)	EPIC-Norfolk	Total CVD	1662	353	0.96 (0.91, 1.01)
Liu 2023 (31)	UK Biobank	CVD incidence	7922	1585	0.96 (0.93, 0.99)
Hirahatake 2019 (20)	WHI	CVD incidence	5809	1454	0.95 (0.92, 0.98)
Bonaccio 2016 (2)	MOLI-SANI	CVD mortality	1995	51	0.87 (0.76, 1.00)
Overall, DL (I^2 = 0.0%, p = 0.485)					0.95 (0.93, 0.97)

.5 1 2

C)

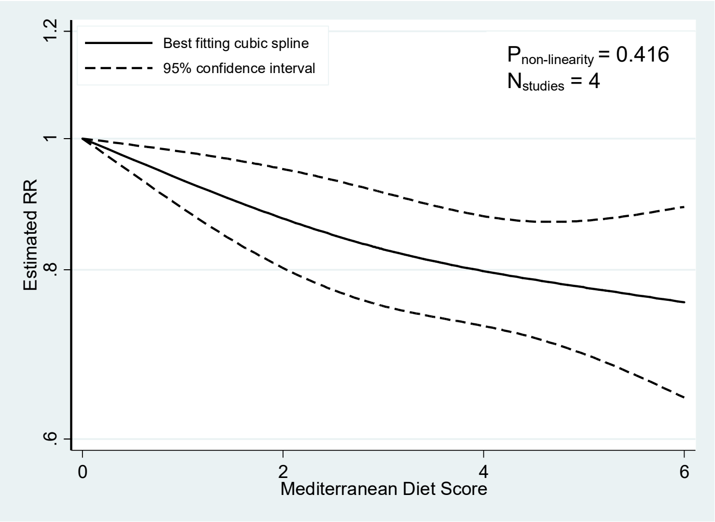

Figure S2. Forest plots for the association between Dietary Approaches to Stop Hypertension and cardiovascular diseases. A) high vs low adherence, B) linear-dose-response (per 5 points) and C) non-linear dose-response meta-analysis.

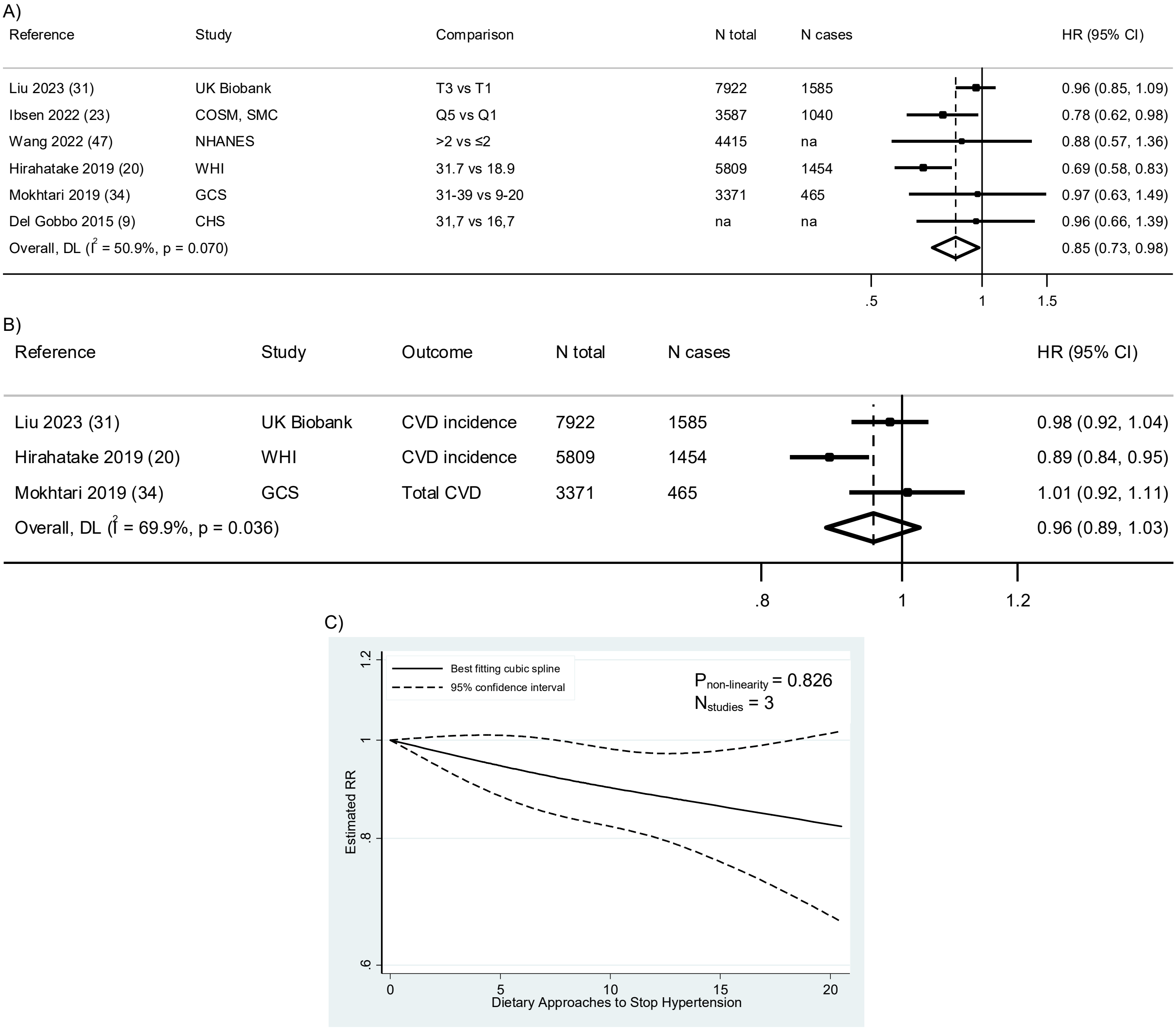

Figure S3. Forest plot for the association between the plant-based dietary index and cardiovascular diseases (high vs low meta-analysis).

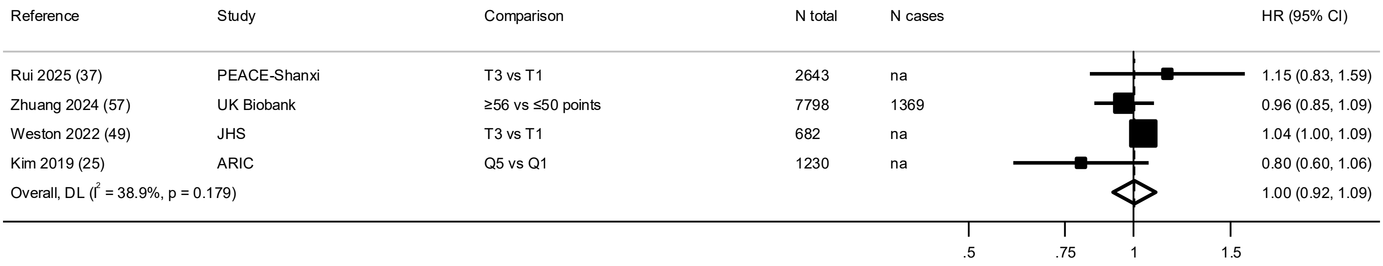

Figure S4. Forest plots for the association between the healthy plant-based dietary index and cardiovascular diseases. A) high vs low adherence, and B) linear-dose-response (per 5 points).

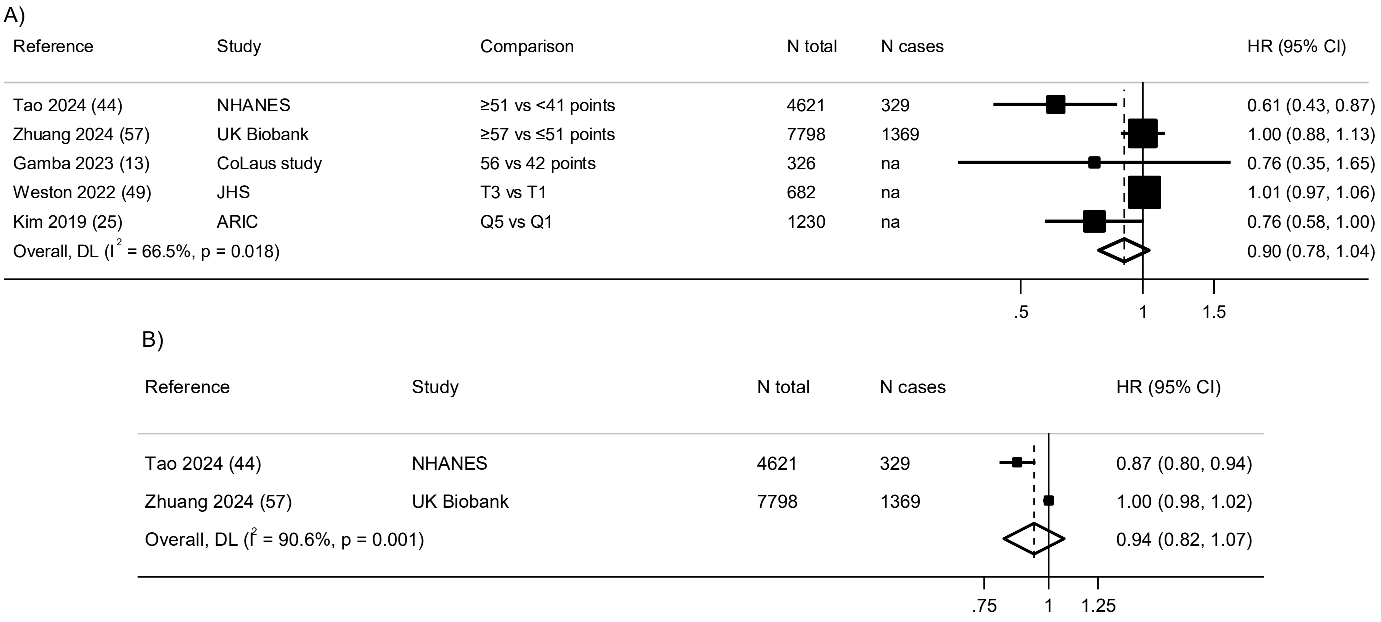

Figure S5. Forest plots for the association between the unhealthy plant-based dietary index and cardiovascular diseases. A) high vs low adherence, and B) linear-dose-response (per 5 points).

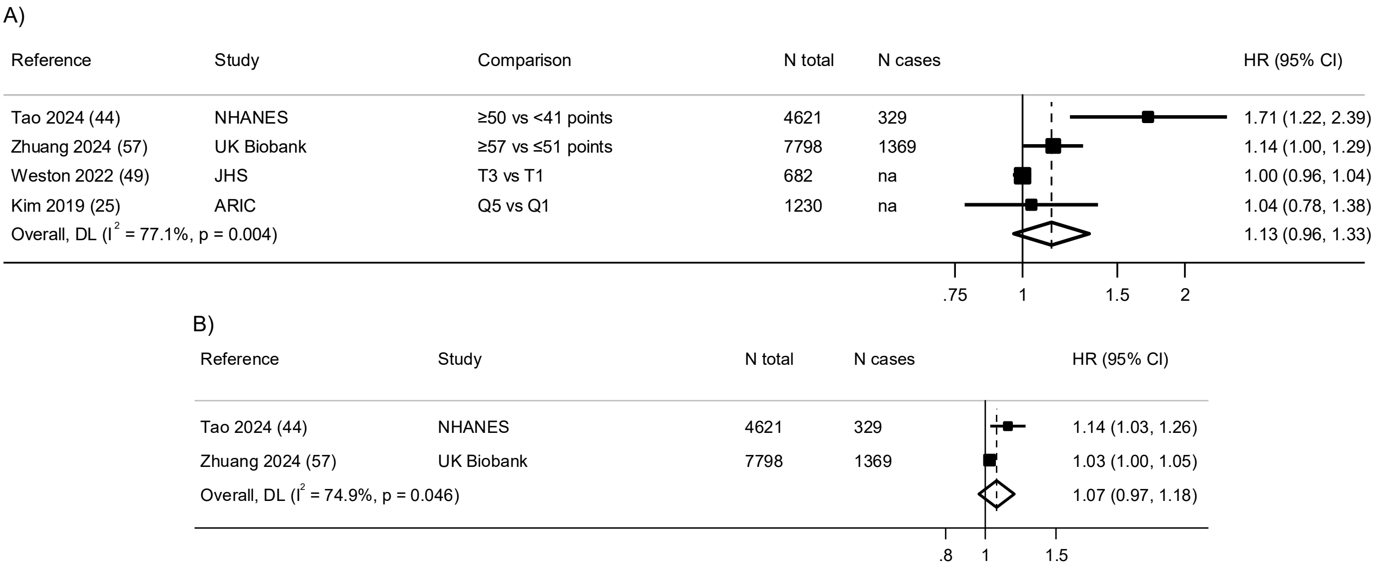

Figure S6. Forest plot for the association between the EAT-Lancet planetary health diet and cardiovascular diseases (linear dose-response per 3 points).

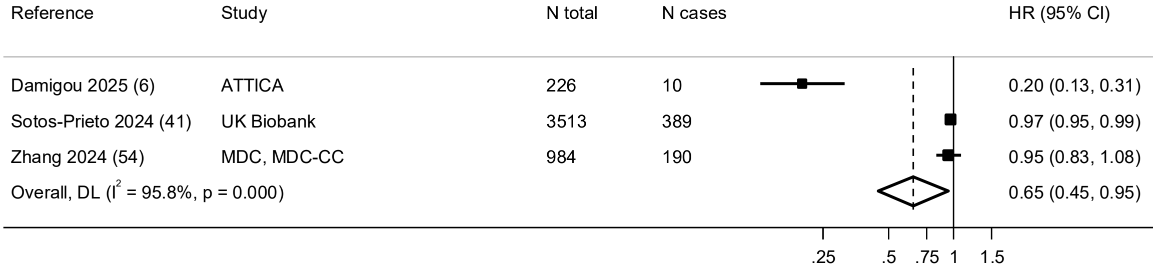

Figure S7. Forest plot for the association between adherence to the (alternate) Healthy Eating Index and cardiovascular diseases (high vs low adherence).

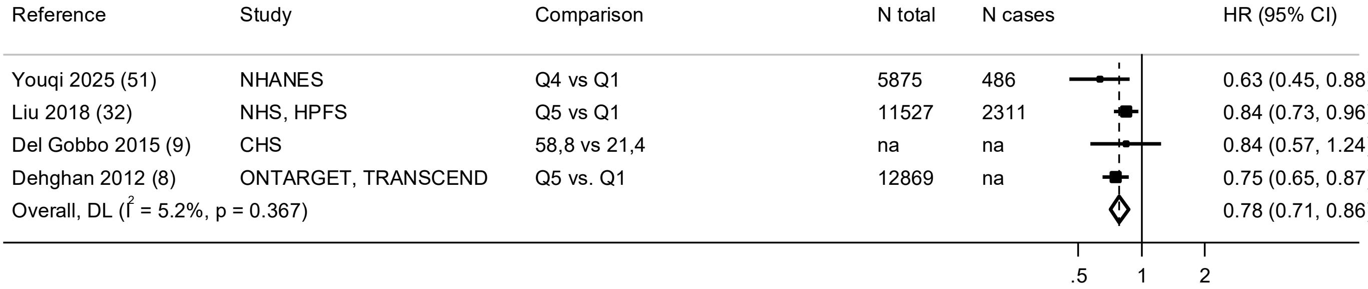

Figure S8. Forest plot for the association between adherence to the Chinese Dietary Guideline and cardiovascular diseases (high vs low adherence).

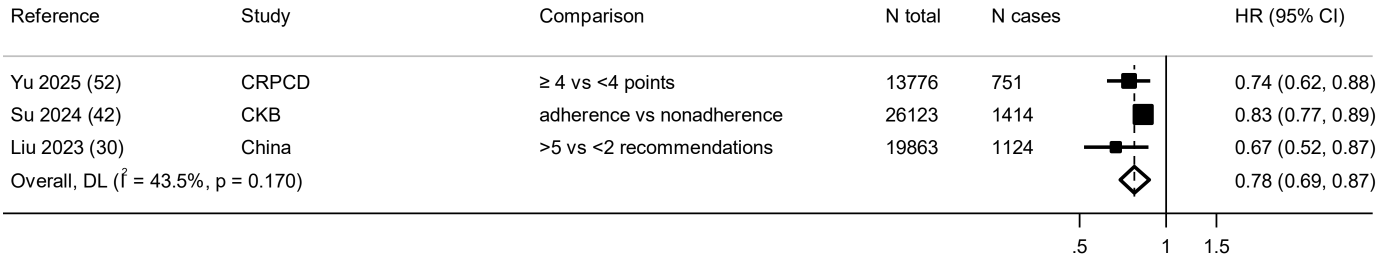

Figure S9. Forest plot for the association between adherence to European dietary guidelines and cardiovascular diseases (high vs low adherence).

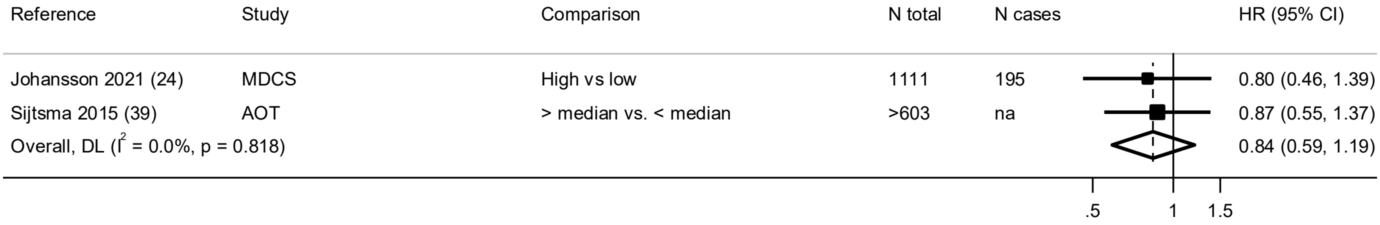

Figure S10. Forest plots for the overall association between adherence to National dietary guidelines and cardiovascular diseases (high vs low adherence).

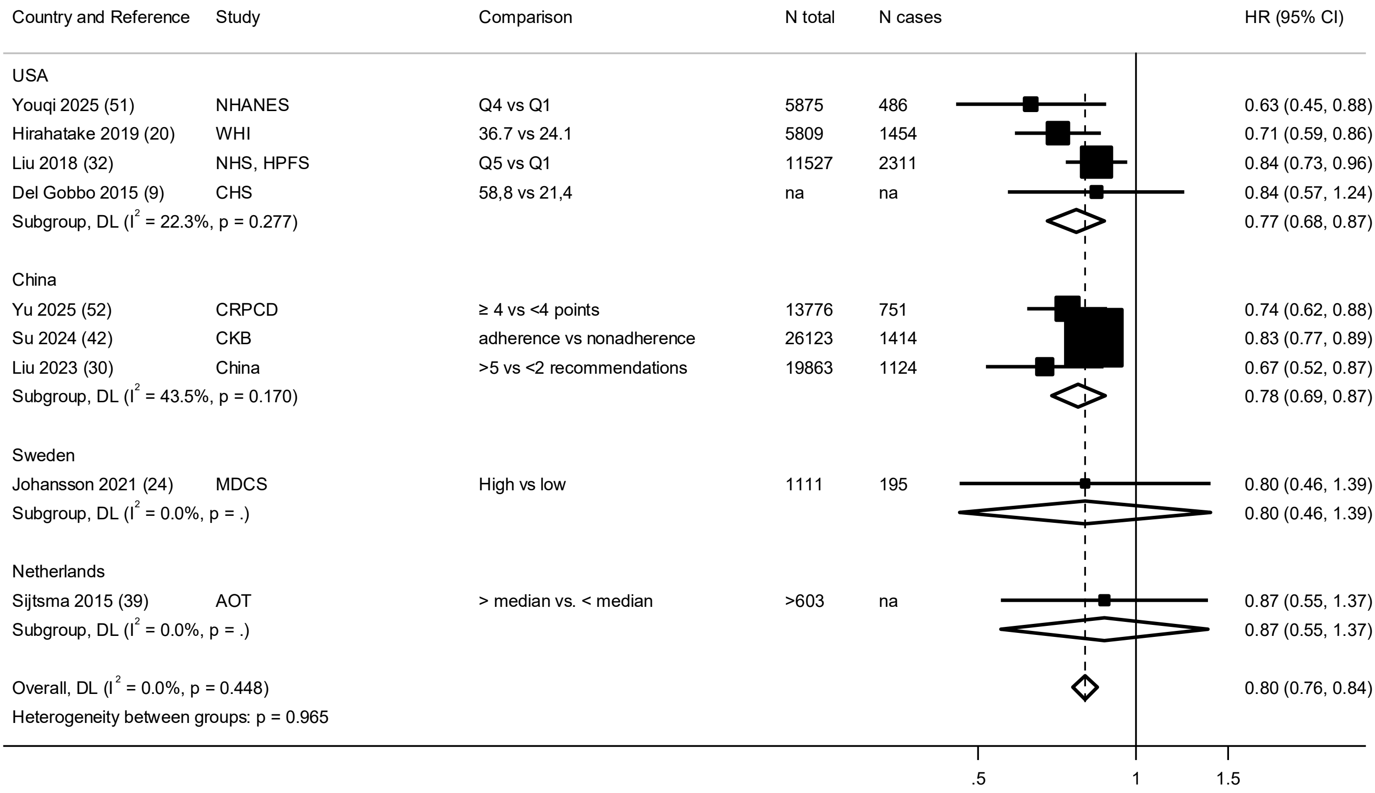

Figure S11. Forest plot for the association between glycemic load and cardiovascular diseases (linear-dose-response per 5 units).

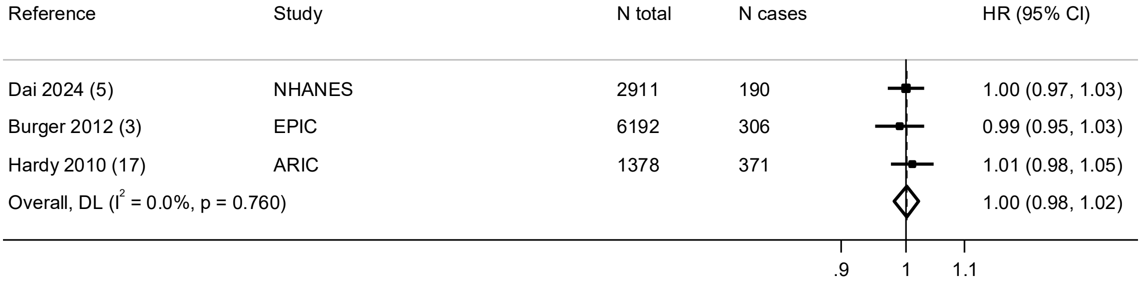

Figure S12. Forest plot for the association between glycemic index and cardiovascular diseases (linear-dose-response per 5 units).

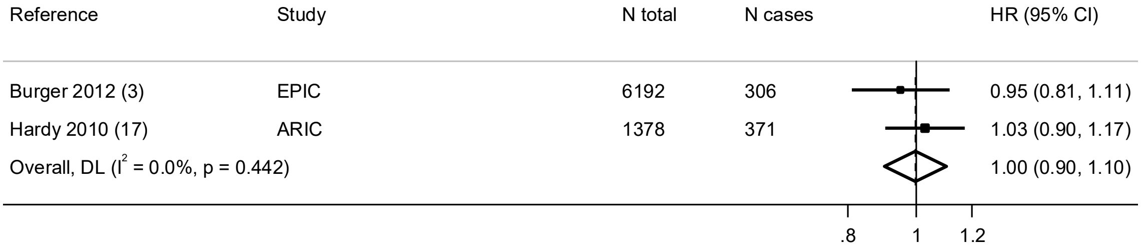

Figure S13. Forest plots for the association between adherence to a low-carbohydrate diet and cardiovascular diseases. A) high vs low adherence, B) linear-dose-response (per 5 points) and C) non-linear dose-response meta-analysis.

A)

Reference	Study/Country	Comparison	N total	N cases	HR (95% CI)
Mita 2025 (33)	Japan	Q4 vs Q1	731	50	0.24 (0.08, 0.72)
Hu 2023 (21)	NHS, HPFS	25.5 vs 9.5	10101	1389	0.86 (0.71, 1.04)
Sun 2022 (43)	GBCS	24 vs 6	2790	411	1.15 (0.87, 1.53)
Nilsson 2012 (36)	VIP	>14 vs <8 points	2141	70	0.51 (0.25, 1.05)
Overall, DL (I^2 = 72.8%, p = 0.012)					0.76 (0.51, 1.14)

Axis: .25 .5 1 1.5

B)

Reference	Study/Country	N total	N cases	HR (95% CI)
Mita 2025 (33)	Japan	731	50	0.76 (0.57, 1.01)
Hu 2023 (21)	NHS, HPFS	10101	1389	0.96 (0.91, 1.01)
Sun 2022 (43)	GBCS	2790	411	1.05 (0.97, 1.14)
Nilsson 2012 (36)	VIP	2141	70	0.73 (0.59, 0.91)
Overall, DL (I^2 = 78.0%, p = 0.003)				0.92 (0.81, 1.04)

Axis: .5 1 1.5

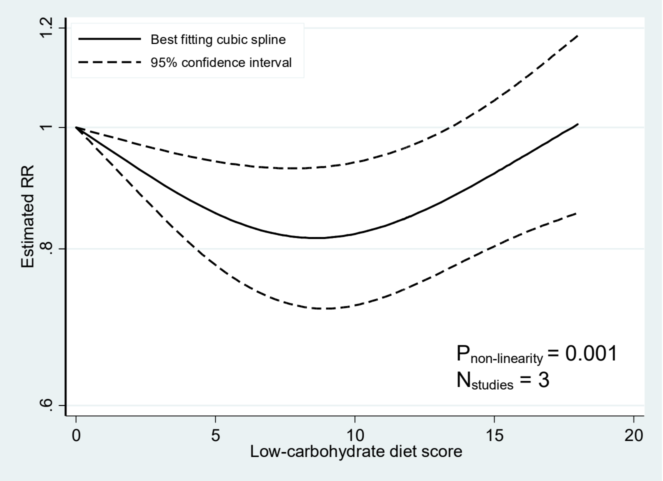

Figure S14. Forest plots for the association between adherence to a vegetable-based low-carbohydrate diet and cardiovascular diseases. A) high vs low adherence, B) linear-dose-response (per 5 points) and C) non-linear dose-response meta-analysis.

A)

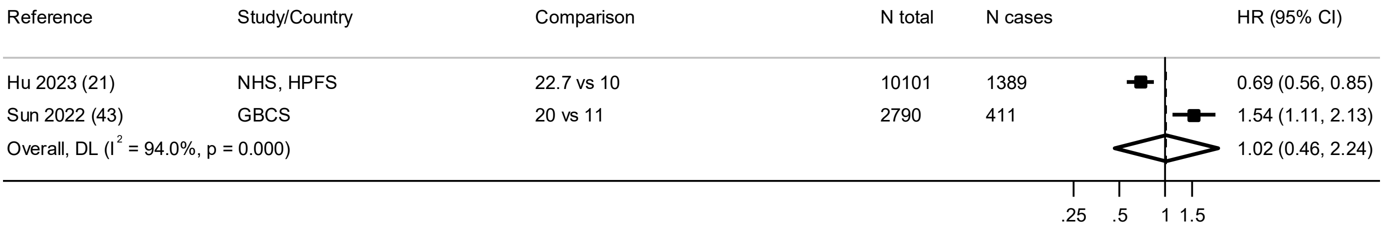

B)

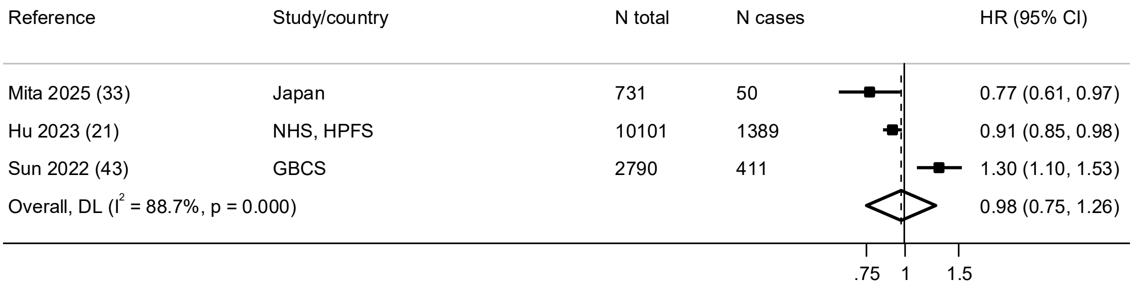

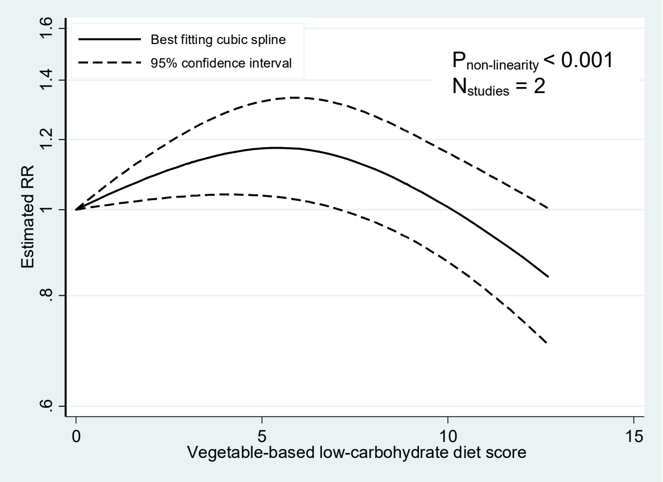

Figure S15. Forest plots for the association between adherence to a meat-based low-carbohydrate diet and cardiovascular diseases. A) high vs low adherence, B) linear-dose-response (per 5 points) and C) non-linear dose-response meta-analysis.

A)

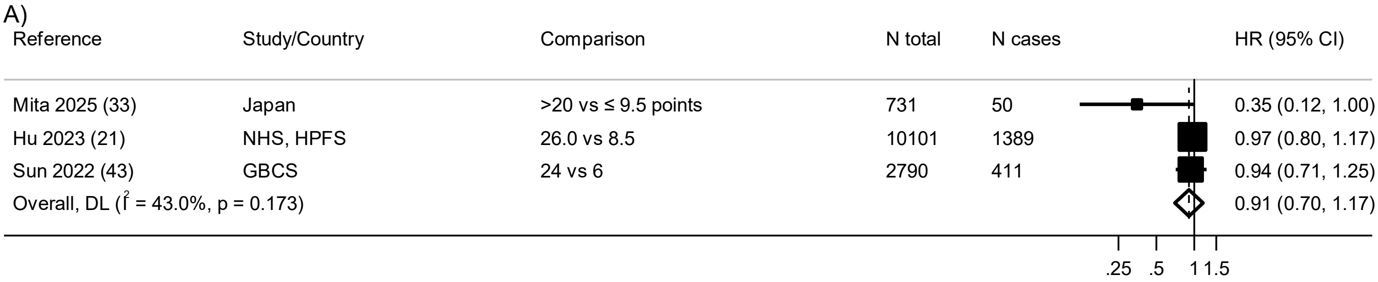

B)

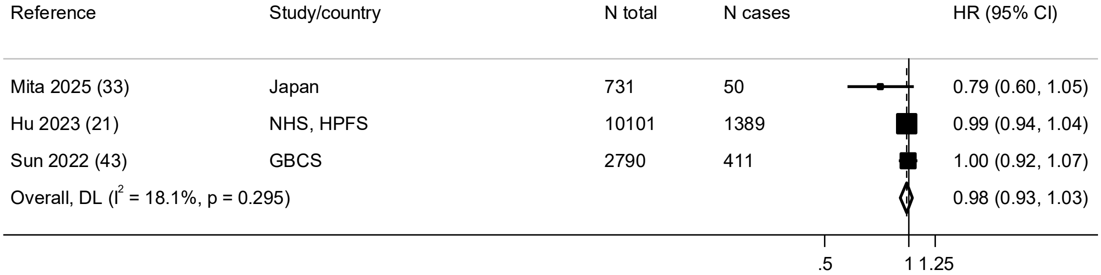

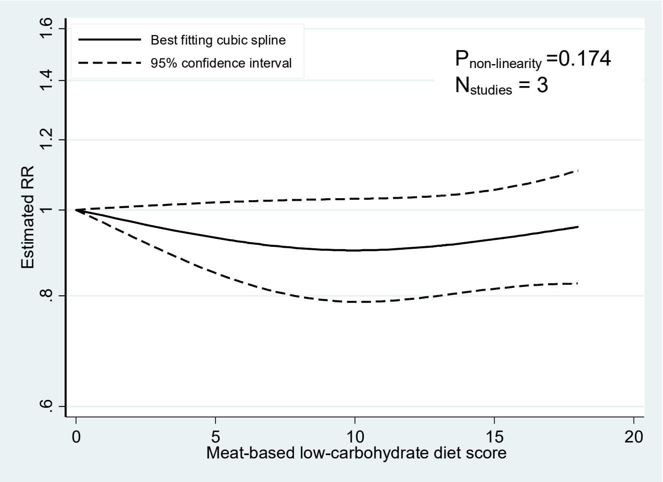

Figure S16. Forest plot for the association between adherence to the dietary inflammatory index and cardiovascular diseases (high vs low adherence).

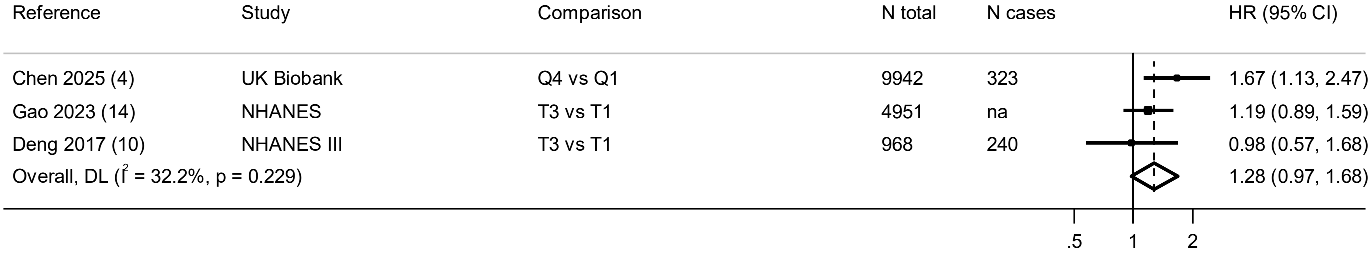

References

1. Higgins JPT, et al. A tool to assess risk of bias in non-randomized follow-up studies of exposure effects (ROBINS-E). Environ Int. 2024;186:108602.
2. Bonaccio M, et al. Adherence to the traditional Mediterranean diet and mortality in subjects with diabetes. Prospective results from the MOLI-SANI study. Eur J Prev Cardiol. 2016;23(4):400-7.
3. Burger KN, et al. Dietary fiber, carbohydrate quality and quantity, and mortality risk of individuals with diabetes mellitus. Plos One. 2012;7(8):e43127.
4. Chen X, et al. Diabetes duration-specific association of dietary inflammatory index with the risk of mortality among individuals with diabetes. Diabetol Metab Syndr. 2025;17(1):243.
5. Dai J, et al. Association of Energy Intake and Dietary Glycemic Load in Different Time Periods With Cardiovascular Disease Mortality Among U.S. Adults With Type 2 Diabetes. Diabetes Care. 2024;47(12):2172-9.
6. Damigou E, et al. Sustainable, planetary healthy dietary patterns are associated with lower 20-year incidence of cardiovascular disease: the ATTICA study (2002-2022). Eur J Clin Nutr. 2025;79(6):536-43.
7. Damigou E, et al. Prevented fractions of cardiovascular disease cases, by long-term adherence to the Mediterranean diet; the ATTICA study (2002-2022). Nutr Metab Cardiovasc Dis. 2025;35(5):103777.
8. Dehghan M, et al. Relationship between healthy diet and risk of cardiovascular disease among patients on drug therapies for secondary prevention: a prospective cohort study of 31 546 high-risk individuals from 40 countries. Circulation. 2012;126(23):2705-12.
9. Del Gobbo LC, et al. Contribution of Major Lifestyle Risk Factors for Incident Heart Failure in Older Adults. The Cardiovascular Health Study. JACC: Heart Failure. 2015;3(7):520-8.
10. Deng FE, et al. Association between diet-related inflammation, all-cause, all-cancer, and cardiovascular disease mortality, with special focus on prediabetics: findings from NHANES III. Eur J Nutr. 2017;56(3):1085-93.
11. Fan M, et al. Association of oxidative balance score with cardiovascular disease and all-cause and cardiovascular mortality in American adults with type 2 diabetes: data from the National Health and Nutrition examination survey 1999-2018. Front Endocrinol (Lausanne). 2024;15:1458039.
12. Gamba M, et al. Association between Total Dietary Phytochemical Intake and Cardiometabolic Health Outcomes-Results from a 10-Year Follow-Up on a Middle-Aged Cohort Population. Nutrients. 2023;15(22).
13. Gao Y, et al. The Relationship Between Dietary Inflammatory Index and All-Cause, Cardiovascular Disease-Related, and Cancer-Related Mortality. J Multidiscip Healthc. 2023;16:2543-56.
14. Ghaemi F, et al. Effects of a Mediterranean diet on the development of diabetic complications: A longitudinal study from the nationwide diabetes report of the National Program for Prevention and Control of Diabetes (NPPCD 2016-2020). Maturitas. 2021;153:61-7.
15. Han Y, et al. Lifestyle, cardiometabolic disease, and multimorbidity in a prospective Chinese study. Eur Heart J. 2021;42(34):3374-84.
16. Hardy DS, et al. Association of glycemic index and glycemic load with risk of incident coronary heart disease among Whites and African Americans with and without type 2 diabetes: the Atherosclerosis Risk in Communities study. Ann Epidemiol. 2010;20(8):610-6.
17. Harriss LR, et al. Dietary patterns and cardiovascular mortality in the Melbourne Collaborative Cohort Study. American Journal of Clinical Nutrition. 2007;86(1):221-9.

18. He P, et al. Association of a Healthy Lifestyle, Life's Essential 8 Scores With Incident Macrovascular and Microvascular Disease Among Individuals With Type 2 Diabetes. J Am Heart Assoc. 2023;12(17):e029441.

19. Hirahatake KM, et al. Diet Quality and Cardiovascular Disease Risk in Postmenopausal Women With Type 2 Diabetes Mellitus: The Women's Health Initiative. Journal of the American Heart Association. 2019;8(19).

20. Hu Y, et al. Low-Carbohydrate Diet Scores and Mortality Among Adults With Incident Type 2 Diabetes. Diabetes Care. 2023;46(4):874-84.

21. Huang ZG, et al. Cardiovascular health metrics defined by Life's Essential 8 scores and subsequent macrovascular and microvascular complications in individuals with type 2 diabetes: A prospective cohort study. Diabetes, Obesity and Metabolism. 2024;26(7):2673-83.

22. Ibsen DB, et al. The DASH diet is associated with a lower risk of heart failure: a cohort study. Eur J Prev Cardiol. 2022;29(7):1114-23.

23. Johansson A, et al. Modifiable and non-modifiable risk factors for atherothrombotic ischemic stroke among subjects in the malmö diet and cancer study. Nutrients. 2021;13(6).

24. Kim H, et al. Plant-Based Diets Are Associated With a Lower Risk of Incident Cardiovascular Disease, Cardiovascular Disease Mortality, and All-Cause Mortality in a General Population of Middle-Aged Adults. J Am Heart Assoc. 2019;8(16):e012865.

25. Lara KM, et al. Dietary Patterns and Incident Heart Failure in U.S. Adults Without Known Coronary Disease. Journal of the American College of Cardiology. 2019;73(16):2036-45.

26. Li W, et al. Life's Essential 8 in Relation to Cardiovascular Disease and Mortality in Individuals With Diabetes. JACC: Asia. 2024;4(6):456-64.

27. Liu S, et al. Quality of plant-based diets in relation to all-cause and cardiovascular disease mortality in US adults with sarcopenia: a population-based study. Aging Clin Exp Res. 2025;37(1):176.

28. Liu YJ, et al. Relationship of microvascular complications and healthy lifestyle with all-cause and cardiovascular mortality in women compared with men with type 2 diabetes. Clinical Nutrition. 2024;43(4):1033-40.

29. Liu K, et al. Association of category of dietary intake and physical activity with the risk of mortality in patients with type 2 diabetes mellitus: a prospective cohort study. Zhonghua liu xing bing xue za zhi = Zhonghua liuxingbingxue zazhi. 2023;44(10):1591-8.

30. Liu X, et al. Healthy dietary patterns and risk of cardiovascular disease in diabetic patients: a prospective cohort study. Food & function. 2023;14(18):8604-14.

31. Liu G, et al. Influence of Lifestyle on Incident Cardiovascular Disease and Mortality in Patients With Diabetes Mellitus. Journal of the American College of Cardiology. 2018;71(25):2867-76.

32. Mita T, et al. Relationship of carbohydrate intake proportion to cardiovascular events in Japanese people with type 2 diabetes mellitus. J Clin Endocrinol Metab. 2025.

33. Mokhtari Z, et al. Adherence to the Dietary Approaches to Stop Hypertension (DASH) diet and risk of total and cause-specific mortality: results from the Golestan Cohort Study. Int J Epidemiol. 2019;48(6):1824-38.

34. Murai K, et al. Impact of health practice index and cardiovascular health metrics on incident cardiovascular disease according to glucose tolerance status. Diabetology International. 2024;15(3):456-64.

35. Nilsson LM, et al. Low-carbohydrate, high-protein score and mortality in a northern Swedish population-based cohort. Eur J Clin Nutr. 2012;66(6):694-700.

36. Pierucci P, et al. Diet and myocardial infarction: A nested case-control study in a cohort of elderly subjects in a Mediterranean area of southern Italy. Nutrition, Metabolism and Cardiovascular Diseases. 2012;22(9):727-33.

37. Rui F, et al. The Association Between the Plant-Based Dietary Pattern and Cardiovascular Events Risk Across Various Cardiovascular Disease Risk Populations in Northern China: A Cross-Sectional and Longitudinal Analysis from the China PEACE Project. J Nutr. 2025;155(8):2534-44.

38. Sattler ELP, et al. Changes in Cardiovascular Health at Midlife and Subsequent Cardiovascular Outcomes in Individuals With Diabetes. JACC Adv. 2025;4(1):101450.

39. Sijtsma FP, et al. Healthy eating and lower mortality risk in a large cohort of cardiac patients who received state-of-the-art drug treatment. Am J Clin Nutr. 2015;102(6):1527-33.

40. Song Y, et al. Association Between MIND Diet Adherence and Mortality: Insights from Diabetic and Non-Diabetic Cohorts. Nutr Diabetes. 2023;13(1):18.

41. Sotos-Prieto M, et al. Association between Planetary Health Diet and Cardiovascular Disease: A Prospective Study from the UK Biobank. Eur J Prev Cardiol. 2024.

42. Su J, et al. Association of lifestyle with reduced stroke risk in 41 314 individuals with diabetes: Two prospective cohort studies in China. Diabetes Obes Metab. 2024;26(7):2869-80.

43. Sun C, et al. Low-Carbohydrate Diets and Mortality in Older Asian People: A 15-Year Follow-Up from a Prospective Cohort Study. Nutrients. 2022;14(7):1406.

44. Tao HW, et al. Plant-based diets, mediating biomarkers, and mortality risk among adults with diabetes or prediabetes. Food Funct. 2024;15(8):4223-32.

45. Tektonidis TG, et al. Adherence to a Mediterranean diet is associated with reduced risk of heart failure in men. Eur J Heart Fail. 2016;18(3):253-9.

46. Wang Q, et al. Prospective Association of the Mediterranean Diet with the Onset of Cardiometabolic Multimorbidity in a UK-Based Cohort: The EPIC-Norfolk Study. Journal of Nutrition. 2024;154(12):3761-9.

47. Wang JS, et al. Associations of Adherence to the DASH Diet and the Mediterranean Diet With All-Cause Mortality in Subjects With Various Glucose Regulation States. Front Nutr. 2022;9:828792.

48. Wang W, et al. Dietary Antioxidant Indices in Relation to All-Cause and Cause-Specific Mortality Among Adults With Diabetes: A Prospective Cohort Study. Front Nutr. 2022;9:849727.

49. Weston LJ, et al. Plant-based diets and incident cardiovascular disease and all-cause mortality in African Americans: A cohort study. PLoS Med. 2022;19(1):e1003863.

50. Xie J, et al. Association between daily eating frequency and mortality in people with diabetes: Findings from NHANES 1999-2014. Front Nutr. 2023;10:937771.

51. Youqi Z, et al. Sex-specific associations between diet quality and mortality in adults with diabetes: findings from NHANES 2001-2018. Front Nutr. 2025;12:1576983.

52. Yu H, et al. A healthy lifestyle pattern and mortality risk in patients of type 2 diabetes mellitus: a prospective cohort study in China. J Biomed Res. 2025:1-9.

53. Yuan S, et al. Trends in dietary patterns over the last decade and their association with long-term mortality in general US populations with undiagnosed and diagnosed diabetes. Nutrition and Diabetes. 2023;13(1).

54. Zhang S, et al. The EAT-Lancet Diet Index, Plasma Proteins, and Risk of Heart Failure in a Population-Based Cohort. JACC: Heart Failure. 2024;12(7):1197-208.

55. Zhang YB, et al. Association of Combined Healthy Lifestyles With Cardiovascular Disease and Mortality of Patients With Diabetes: An International Multicohort Study. Mayo Clin Proc. 2023;98(1):60-74.

56. Zhang J, et al. Dose-response relationship between dietary antioxidant intake and diabetic kidney disease in the US adults with diabetes. Acta Diabetol. 2023;60(10):1365-75.

57. Zhuang P, et al. Unhealthy plant-based diet is associated with a higher cardiovascular disease risk in patients with prediabetes and diabetes: a large-scale population-based study. BMC Med. 2024;22(1):485.

58. Zhu K, et al. Modifiable Lifestyle Factors, Genetic Risk, and Incident Peripheral Artery Disease Among Individuals With Type 2 Diabetes: A Prospective Study. Diabetes Care. 2024;47(3):435-43.

59. Abris GP, et al. Cause-specific and all-cause mortalities in vegetarian compared with those in nonvegetarian participants from the Adventist Health Study-2 cohort. American Journal of Clinical Nutrition. 2024;120(4):907-17.

60. Ahmed HN, et al. Coffee consumption and risk of heart failure in men: An analysis from the Cohort of Swedish Men. American Heart Journal. 2009;158(4):667-72.

61. Åkesson A, et al. Dietary exposure to polychlorinated biphenyls and risk of heart failure - A population-based prospective cohort study. Environ Int. 2019;126:1-6.

62. Åkesson A, et al. Low-risk diet and lifestyle habits in the primary prevention of myocardial infarction in men: A population-based prospective cohort study. Journal of the American College of Cardiology. 2014;64(13):1299-306.

63. Åkesson A, et al. Combined effect of low-risk dietary and lifestyle behaviors in primary prevention of myocardial infarction in women. Archives of Internal Medicine. 2007;167(19):2122-7.

64. Akter S, et al. Dietary acid load and mortality among Japanese men and women: The Japan Public Health Center-based Prospective Study. American Journal of Clinical Nutrition. 2017;106(1):146-54.

65. Al-Ramady O, et al. Egg consumption and risk of acute stroke in the Million Veteran Program. Clinical Nutrition ESPEN. 2022;50:178-82.

66. Aleksova A, et al. The Co-Existence of Hypovitaminosis D and Diabetes Mellitus Triples the Incidence of Severe Coronary Artery Disease in Women. J Clin Med. 2024;13(22).

67. Amba V, et al. Nut and peanut butter consumption and mortality in the national institutes of health-AARP diet and health study. Nutrients. 2019;11(7).

68. Andersen LF, et al. Consumption of coffee is associated with reduced risk of death attributed to inflammatory and cardiovascular diseases in the Iowa Women's Health Study. American Journal of Clinical Nutrition. 2006;83(5):1039-46.

69. Anderson JL, et al. Relation of vitamin D deficiency to cardiovascular risk factors, disease status, and incident events in a general healthcare population. Am J Cardiol. 2010;106(7):963-8.

70. Anderson JL, et al. Parathyroid hormone, vitamin D, renal dysfunction, and cardiovascular disease: dependent or independent risk factors? Am Heart J. 2011;162(2):331-9.e2.

71. Arts ICW, et al. Dietary catechins in relation to coronary heart disease death among postmenopausal women. Epidemiology. 2001;12(6):668-75.

72. Asghari G, et al. Association of Dietary Diabetes Risk Reduction Score With Risk of Cardiovascular Diseases in the Iranian Population: Tehran Lipid and Glucose Study. Heart Lung Circ. 2022;31(1):101-9.

73. Avalos EE, et al. Is dairy product consumption associated with the incidence of CHD? Public health nutrition. 2013;16(11):2055-63.

74. Baden MY, et al. Changes in Plant-Based Diet Quality and Total and Cause-Specific Mortality. Circulation. 2019;140(12):979-91.

75. Baer HJ, et al. Risk factors for mortality in the nurses' health study: a competing risks analysis. Am J Epidemiol. 2011;173(3):319-29.

76. Bao Y, et al. Association of nut consumption with total and cause-specific mortality. N Engl J Med. 2013;369(21):2001-11.

77. Bernstein AM, et al. Cereal fiber and coronary heart disease: A comparison of modeling approaches for repeated dietary measurements, intermediate outcomes, and long follow-up. European Journal of Epidemiology. 2011;26(11):877-86.

78. Block RC, et al. Predicting Risk for Incident Heart Failure With Omega-3 Fatty Acids: From MESA. JACC Heart Fail. 2019;7(8):651-61.

79. Bodar V, et al. Coffee consumption and risk of heart failure in the Physicians' Health Study. Clinical Nutrition ESPEN. 2020;40:133-7.

80. Bodar V, et al. Consumption of potatoes and incidence rate of coronary artery disease: The Million Veteran Program. Clin Nutr ESPEN. 2021;42:201-5.

81. Boekholdt SM, et al. Plasma concentrations of ascorbic acid and C-reactive protein, and risk of future coronary artery disease, in apparently healthy men and women: the EPIC-Norfolk prospective population study. Br J Nutr. 2006;96(3):516-22.

82. Bos MJ, et al. Modifiable Etiological Factors and the Burden of Stroke from the Rotterdam Study: A Population-Based Cohort Study. PLoS Medicine. 2014;11(4).

83. Brassard D, et al. Greater adherence to the 2019 Canada's Food Guide recommendations on healthy food choices reduces the risk of cardiovascular disease in adults: a prospective analysis of UK Biobank data. American Journal of Clinical Nutrition. 2022;116(6):1748-58.

84. Braverman-Bronstein A, et al. Mortality attributable to sugar sweetened beverages consumption in Mexico: an update. International Journal of Obesity. 2020;44(6):1341-9.

85. Brazionis L, et al. Plasma retinol: a novel marker for cardiovascular disease mortality in Australian adults. Nutr Metab Cardiovasc Dis. 2012;22(10):914-20.

86. Bui LP, et al. Planetary Health Diet Index and risk of total and cause-specific mortality in three prospective cohorts. American Journal of Clinical Nutrition. 2024;120(1):80-91.

87. Buil-Cosiales P, et al. Association between dietary fibre intake and fruit, vegetable or whole-grain consumption and the risk of CVD: results from the PREvención con DIeta MEDiterránea (PREDIMED) trial. Br J Nutr. 2016;116(3):534-46.

88. Cahill LE, et al. Prospective study of breakfast eating and incident coronary heart disease in a cohort of male US health professionals. Circulation. 2013;128(4):337-43.

89. Cai H, et al. A prospective study of dietary patterns and mortality in Chinese women. Epidemiology. 2007;18(3):393-401.

90. Cangemi R, et al. Cholesterol-adjusted vitamin E serum levels are associated with cardiovascular events in patients with non-valvular atrial fibrillation. Int J Cardiol. 2013;168(4):3241-7.

91. Cao Y, Yu Y. Associations between Cholesterol Intake, Food Sources and Cardiovascular Disease in Chinese Residents. Nutrients. 2024;16(5).

92. Carroll HA, et al. The association between water intake and future cardiometabolic disease outcomes in the Malmö Diet and Cancer cardiovascular cohort. PLoS One. 2024;19(1):e0296778.

93. Casiglia E, et al. High dietary fiber intake prevents stroke at a population level. Clin Nutr. 2013;32(5):811-8.

94. Chang AR, et al. High dietary phosphorus intake is associated with all-cause mortality: results from NHANES III. Am J Clin Nutr. 2014;99(2):320-7.

95. Chang Y, et al. Impact of modifiable healthy lifestyles on mortality in Chinese older adults. Scientific reports. 2024;14(1):28869.

96. Chasan-Taber L, et al. A prospective study of folate and vitamin B6 and risk of myocardial infarction in US physicians. Journal of the American College of Nutrition. 1996;15(2):136-43.

97. Chazelas E, et al. Sugary Drinks, Artificially-Sweetened Beverages, and Cardiovascular Disease in the NutriNet-Santé Cohort. Journal of the American College of Cardiology. 2020;76(18):2175-7.

98. Che J, et al. Dietary n-3 Fatty Acids Intake and All-Cause and Cardiovascular Mortality in Patients With Prediabetes and Diabetes. Journal of Clinical Endocrinology and Metabolism. 2024;109(11):2847-56.

99. Chen W, et al. Household air pollution, adherence to a healthy lifestyle, and risk of cardiometabolic multimorbidity: Results from the China health and retirement longitudinal study. Sci Total Environ. 2023;855:158896.

100. Chen Z, et al. Dietary phytoestrogens and total and cause-specific mortality: results from 2 prospective cohort studies. American Journal of Clinical Nutrition. 2023;117(1):130-40.

101. Chiu THT, et al. Vegetarian diet and incidence of total, ischemic, and hemorrhagic stroke in 2 cohorts in Taiwan. Neurology. 2020;94(11):e1112-e21.

102. Chiuve SE, et al. Primary prevention of stroke by healthy lifestyle. Circulation. 2008;118(9):947-54.

103. Chiuve SE, et al. Dietary and plasma magnesium and risk of coronary heart disease among women. Journal of the American Heart Association. 2013;2(2).

104. Cho HJ, et al. Association of coffee drinking with all-cause and cause-specific mortality in over 190,000 individuals: data from two prospective studies. Int J Food Sci Nutr. 2022;73(4):513-21.

105. Chomistek AK, et al. Healthy lifestyle in the primordial prevention of cardiovascular disease among young women. Journal of the American College of Cardiology. 2015;65(1):43-51.

106. Collin LJ, et al. Association of Sugary Beverage Consumption With Mortality Risk in US Adults: A Secondary Analysis of Data From the REGARDS Study. JAMA Netw Open. 2019;2(5):e193121.

107. Conrad Z, et al. Prospective Analysis of Vegetable Amount and Variety on the Risk of All-Cause and Cause-Specific Mortality among US Adults, 1999⁻2011. Nutrients. 2018;10(10).

108. Cruijsen E, et al. Potato Consumption and Risk of Cardiovascular Mortality and Type 2 Diabetes After Myocardial Infarction: A Prospective Analysis in the Alpha Omega Cohort. Front Nutr. 2021;8:813851.

109. Currenti W, et al. Dietary Fats and Cardio-Metabolic Outcomes in a Cohort of Italian Adults. Nutrients. 2022;14(20).

110. Da H, et al. Association of a low-inflammatory diet with survival among adults: The role of cardiometabolic diseases and lifestyle. Clin Nutr. 2024;43(4):943-50.

111. da Silva Canhos MM, et al. Association between vitamin D levels and mortality in hemodialysis patients: a cohort study. Ren Fail. 2020;42(1):225-33.

112. Damigou E, et al. Mediterranean Diet and Cardiovascular Disease: The Moderating Role of Adequate Sleep—Results from the ATTICA Cohort Study (2002–2022). Nutrients. 2024;16(1).

113. Damigou E, et al. Diet Quality and Consumption of Healthy and Unhealthy Foods Measured via the Global Diet Quality Score in Relation to Cardiometabolic Outcomes in Apparently Healthy Adults from the Mediterranean Region: The ATTICA Epidemiological Cohort Study (2002-2022). Nutrients. 2023;15(20).

114. Damigou E, et al. Lifestyle Trajectories Are Associated with Incidence of Cardiovascular Disease: Highlights from the ATTICA Epidemiological Cohort Study (2002-2022). Life (Basel). 2023;13(5).

115. Daraghmeh AH, et al. Evidence for the vitamin D hypothesis: The NHANES III extended mortality follow-up. Atherosclerosis. 2016;255:96-101.

116. Daviglus ML, et al. Dietary vitamin C, beta-carotene and 30-year risk of stroke: Results from the western electric study. Neuroepidemiology. 1997;16(2):69-77.

117. De Koning L, et al. Sweetened beverage consumption, incident coronary heart disease, and biomarkers of risk in men. Circulation. 2012;125(14):1735-41.

118. de Rooij ENM, et al. Serum Potassium and Mortality Risk in Hemodialysis Patients: A Cohort Study. Kidney Medicine. 2022;4(1).

119. Deraz O, et al. Person-centered and measured life's simple 7 cardiovascular health concordance and association with incident cardiovascular disease. Scientific reports. 2023;13(1):5247.

120. Ding L, et al. Ideal cardiovascular health and risk of death in a large Swedish cohort. BMC public health. 2024;24(1):358.

121. Djoussé L, et al. Plasma free fatty acids and risk of heart failure: the Cardiovascular Health Study. Circ Heart Fail. 2013;6(5):964-9.

122. Djoussé L, et al. Consumption of fried foods and risk of heart failure in the physicians' health study. Journal of the American Heart Association. 2015;4(4).

123. Domei T, et al. Ratio of serum n-3 to n-6 polyunsaturated fatty acids and the incidence of major adverse cardiac events in patients undergoing percutaneous coronary intervention. Circ J. 2012;76(2):423-9.

124. Donat-Vargas C, et al. Cardiovascular and cancer mortality in relation to dietary polychlorinated biphenyls and marine polyunsaturated fatty acids: a nutritional-toxicological aspect of fish consumption. Journal of Internal Medicine. 2020;287(2):197-209.

125. Dong JY, et al. Chocolate consumption and risk of stroke among men and women: A large population-based, prospective cohort study. Atherosclerosis. 2017;260:8-12.

126. Dong Z, et al. Association of dietary sulfur amino acid intake with mortality from diabetes and other causes. Eur J Nutr. 2022;61(1):289-98.

127. Dukuzimana J, et al. High consumption of dairy products and risk of major adverse coronary events and stroke in a Swedish population. British Journal of Nutrition. 2024;131(3):500-11.

128. Elliott P, et al. Estimated 24-Hour Urinary Sodium Excretion and Incident Cardiovascular Disease and Mortality among 398628 Individuals in UK Biobank. Hypertension. 2020;76(3):683-91.

129. Erkent I, et al. Determinants of preventable stroke-Ankara ACROSS stroke preventability study. J Stroke Cerebrovasc Dis. 2020;29(7):104825.

130. Eshak ES, et al. Soft drink intake in relation to incident ischemic heart disease, stroke, and stroke subtypes in Japanese men and women: The Japan Public Health Centre-based study cohort I. American Journal of Clinical Nutrition. 2012;96(6):1390-7.

131. Eshak ES, et al. Rice consumption is not associated with risk of cardiovascular disease morbidity or mortality in Japanese men and women: A large population-based, prospective cohort study. American Journal of Clinical Nutrition. 2014;100(1):199-207.

132. Fan X, et al. Vitamin D Status and Risk of All-Cause and Cause-Specific Mortality in a Large Cohort: Results From the UK Biobank. J Clin Endocrinol Metab. 2020;105(10).

133. Fang Z, et al. Association of ultra-processed food consumption with all cause and cause specific mortality: population based cohort study. BMJ. 2024.

134. Farhadnejad H, et al. Low-carbohydrate diet and cardiovascular diseases in Iranian population: Tehran Lipid and Glucose Study. Nutrition, Metabolism and Cardiovascular Diseases. 2020;30(4):581-8.

135. Ferrero-Hernández P, et al. Association between lifestyle risk factors and mortality in the Mexico City prospective study. Sci Rep. 2025;15(1):145.

136. Feskens EJ, et al. Association between fish intake and coronary heart disease mortality. Differences in normoglycemic and glucose intolerant elderly subjects. Diabetes Care. 1993;16(7):1029-34.

137. Fraser GE, et al. Effect of risk factor values on lifetime risk of and age at first coronary event. The Adventist Health Study. Am J Epidemiol. 1995;142(7):746-58.

138. Fretts AM, et al. Plasma phospholipid and dietary α-linolenic acid, mortality, CHD and stroke: the Cardiovascular Health Study. The British journal of nutrition. 2014;112(7):1206-13.

139. Fung TT, et al. Adherence to a DASH-style diet and risk of coronary heart disease and stroke in women. Arch Intern Med. 2008;168(7):713-20.

140. Fung TT, et al. Sweetened beverage consumption and risk of coronary heart disease in women. American Journal of Clinical Nutrition. 2009;89(4):1037-42.

141. Fung TT, et al. Mediterranean diet and incidence of and mortality from coronary heart disease and stroke in women. Circulation. 2009;119(8):1093-100.

142. Fung TT, et al. Prospective study of major dietary patterns and stroke risk in women. Stroke. 2004;35(9):2014-9.

143. Fung TT, et al. Low-carbohydrate diets and all-cause and cause-specific mortality: Two cohort studies. Annals of Internal Medicine. 2010;153(5):289-98.

144. Galbete C, et al. Nordic diet, Mediterranean diet, and the risk of chronic diseases: The EPIC-Potsdam study. BMC Medicine. 2018;16(1).

145. Gao Q, et al. Consumption of flavonoid-rich fruits, flavonoids from fruits and stroke risk: A prospective cohort study. British Journal of Nutrition. 2021;126(11):1717-24.

146. Garagarza C, et al. Hypophosphatemia: nutritional status, body composition, and mortality in hemodialysis patients. International Urology and Nephrology. 2017;49(7):1243-50.

147. Gardener H, et al. Diet soft drink consumption is associated with an increased risk of vascular events in the Northern Manhattan study. Journal of General Internal Medicine. 2012;27(9):1120-6.

148. Geleijnse JM, et al. Dietary intake of menaquinone is associated with a reduced risk of coronary heart disease: the Rotterdam Study. J Nutr. 2004;134(11):3100-5.

149. Georgoulis M, et al. Mediterranean diet trajectories and 20-year incidence of cardiovascular disease: The ATTICA cohort study (2002-2022). Nutr Metab Cardiovasc Dis. 2024;34(1):153-66.

150. Giles WH, et al. Serum folate and risk for ischemic stroke. First National Health and Nutrition Examination Survey epidemiologic follow-up study. Stroke. 1995;26(7):1166-70.

151. Giovannucci E, et al. 25-hydroxyvitamin D and risk of myocardial infarction in men: a prospective study. Arch Intern Med. 2008;168(11):1174-80.

152. Gooding HC, et al. Application of a lifestyle-based tool to estimate premature cardiovascular disease events in young adults the coronary artery risk development in young adults (CARDIA) study. JAMA Internal Medicine. 2017;177(9):1354-60.

153. Gribbin S, et al. Association of carbohydrate and saturated fat intake with cardiovascular disease and mortality in Australian women. Heart. 2022;108(12):932-9.

154. Guo J, et al. Vitamin D intake and risk of CVD and all-cause mortality: evidence from the Caerphilly Prospective Cohort Study. Public health nutrition. 2017;20(15):2744-53.

155. Guo J, et al. Association between egg consumption and cardiovascular disease events, diabetes and all-cause mortality. Eur J Nutr. 2018;57(8):2943-52.

156. Gyntelberg F, et al. Coffee consumption and risk of ischaemic heart disease--a settled issue? J Intern Med. 1995;237(1):55-61.

157. Habibzadeh A, et al. Association between dietary total antioxidant capacity and the risk of stroke: a nested case-control study. BMC Nutr. 2024;10(1):56.

158. Hadaegh F, et al. Ideal cardiovascular health status and risk of cardiovascular disease and all-cause mortality: over a decade of follow-up in the Tehran lipid and glucose study. Frontiers in Cardiovascular Medicine. 2022;9.

159. Hanley-Cook GT, et al. FAU ood:biodiversity Pleasecheckandconfirmwhethertheeditstothea and total and cause-specific mortality in 9 European countries: An analysis of a prospective cohort study. PLoS Medicine. 2021;18(10).

160. Happonen P, et al. Coffee consumption and mortality in a 14-year follow-up of an elderly northern Finnish population. British Journal of Nutrition. 2008;99(6):1354-61.

161. Happonen P, et al. Coffee drinking is dose-dependently related to the risk of acute coronary events in middle-aged men. Journal of Nutrition. 2004;134(9):2381-6.

162. Harbers MC, et al. Adherence to the Dutch dietary guidelines and 15-year incidence of heart failure in the EPIC-NL cohort. European Journal of Nutrition. 2020;59(8):3405-13.

163. Haring B, et al. Dietary protein intake and coronary heart disease in a large community based cohort: results from the Atherosclerosis Risk in Communities (ARIC) study [corrected]. PLoS One. 2014;9(10):e109552.

164. Haring B, et al. Association of Dietary Protein Consumption With Incident Silent Cerebral Infarcts and Stroke: The Atherosclerosis Risk in Communities (ARIC) Study. Stroke. 2015;46(12):3443-50.

165. Hashemian M, et al. Potato consumption and the risk of overall and cause specific mortality in the NIH-AARP study. PLoS ONE. 2019;14(5).

166. Havmoeller R, et al. Elevated plasma free fatty acids are associated with sudden death: a prospective community-based evaluation at the time of cardiac arrest. Heart Rhythm. 2014;11(4):691-6.

167. He K, et al. Dietary fat intake and risk of stroke in male US healthcare professionals: 14 year prospective cohort study. Bmj. 2003;327(7418):777-82.

168. He K, et al. Folate, Vitamin B6, and B12 Intakes in Relation to Risk of Stroke among Men. Stroke. 2004;35(1):169-74.

169. Heath AK, et al. Circulating 25-hydroxyvitamin D concentration and cause-specific mortality in the Melbourne Collaborative Cohort Study. J Steroid Biochem Mol Biol. 2020;198:105612.

170. Heianza Y, et al. Genetic susceptibility, plant-based dietary patterns, and risk of cardiovascular disease. American Journal of Clinical Nutrition. 2020;112(1):220-8.

171. Heidemann C, et al. Dietary patterns and risk of mortality from cardiovascular disease, cancer, and all causes in a prospective cohort of women. Circulation. 2008;118(3):230-7.

172. Hlaing-Hlaing H, et al. Alternative Healthy Eating Index-2010 and Incident Non-Communicable Diseases: Findings from a 15-Year Follow Up of Women from the 1973–78 Cohort of the Australian Longitudinal Study on Women's Health. Nutrients. 2022;14(20).

173. Hodge AM, et al. Dietary inflammatory index or Mediterranean diet score as risk factors for total and cardiovascular mortality. Nutrition, Metabolism and Cardiovascular Diseases. 2018;28(5):461-9.

174. Hodge AM, et al. Does a Mediterranean diet reduce the mortality risk associated with diabetes: Evidence from the Melbourne Collaborative Cohort Study. Nutrition, Metabolism and Cardiovascular Diseases. 2011;21(9):733-9.

175. Hoque B, Shi Z. Association between selenium intake, diabetes and mortality in adults: findings from National Health and Nutrition Examination Survey (NHANES) 2003-2014. Br J Nutr. 2022;127(7):1098-105.

176. Hou W, et al. Relationship Between Carbohydrate Intake (Quantity, Quality, and Time Eaten) and Mortality (Total, Cardiovascular, and Diabetes): Assessment of 2003-2014 National Health and Nutrition Examination Survey Participants. Diabetes Care. 2022;45(12):3024-31.

177. Hsiao PY, et al. Dietary patterns and relationship to obesity-related health outcomes and mortality in adults 75 years of age or greater. Journal of Nutrition, Health and Aging. 2013;17(6):566-72.

178. Hu FB, et al. Dietary saturated fats and their food sources in relation to the risk of coronary heart disease in women. American Journal of Clinical Nutrition. 1999;70(6):1001-8.

179. Hu FB, et al. Dietary fat intake and the risk of coronary heart disease in women. New England Journal of Medicine. 1997;337(21):1491-9.

180. Hu FB, et al. Dietary protein and risk of ischemic heart disease in women. American Journal of Clinical Nutrition. 1999;70(2):221-7.

181. Hu MJ, et al. Effect of Cheese Intake on Cardiovascular Diseases and Cardiovascular Biomarkers. Nutrients. 2022;14(14).

182. Hu Y, et al. Interplay between diet, circulating indolepropionate concentrations and cardiometabolic health in US populations. Gut. 2023;72(12):2260-71.

183. Huang F, et al. Effect of dietary cholesterol intake on stroke incidence among Chinese adults: evidence from China Health and Nutrition Survey. Wei sheng yan jiu = Journal of hygiene research. 2016;45(3):383-7.

184. Huang J, et al. Serum Beta Carotene and Overall and Cause-Specific Mortality. Circ Res. 2018;123(12):1339-49.

185. Huang J, et al. Relationship Between Serum Alpha-Tocopherol and Overall and Cause-Specific Mortality. Circ Res. 2019;125(1):29-40.

186. Huang J, et al. Association between serum retinol and overall and cause-specific mortality in a 30-year prospective cohort study. Nat Commun. 2021;12(1):6418.

187. Huang T, et al. Consumption of whole grains and cereal fiber and total and cause-specific mortality: Prospective analysis of 367,442 individuals. BMC Medicine. 2015;13(1).

188. Hung HC, et al. The association between fruit and vegetable consumption and peripheral arterial disease. Epidemiology. 2003;14(6):659-65.

189. Inagaki Y, et al. Greater change in the eicosapentaenoic acid to arachidonic acid ratio is associated with decreased incidence of cardiovascular events in acute coronary syndrome patients with elevated triglyceride levels. Circulation journal. 2021;85(10):1746-53.

190. Isiozor NM, et al. Serum copper and the risk of cardiovascular disease death in Finnish men. Nutrition, Metabolism and Cardiovascular Diseases. 2023;33(1):151-7.

191. Isiozor NM, et al. Ideal cardiovascular health and risk of acute myocardial infarction among Finnish men. Atherosclerosis. 2019;289:126-31.

192. Iso H, et al. Intake of fish and omega-3 fatty acids and risk of stroke in women. JAMA. 2001;285(3):304-12.

193. Iso H, et al. Linoleic acid, other fatty acids, and the risk of stroke. Stroke. 2002;33(8):2086-93.

194. Ivey KL, et al. Flavonoid intake and all-cause mortality. American journal of clinical nutrition. 2015;101(5):1012-20.

195. Jackson JK, et al. Better diet quality scores are associated with a lower risk of hypertension and non-fatal CVD in middle-aged Australian women over 15 years of follow-up. Public Health Nutr. 2020;23(5):882-93.

196. Janszky I, et al. Chocolate consumption and mortality following a first acute myocardial infarction: The Stockholm Heart Epidemiology Program. Journal of Internal Medicine. 2009;266(3):248-57.

197. Järvinen R, et al. Intake of fish and long-chain n-3 fatty acids and the risk of coronary heart mortality in men and women. British Journal of Nutrition. 2006;95(4):824-9.

198. Jauhiainen R, et al. Novel biomarkers associated with incident heart failure in 10 106 Finnish men. ESC Heart Fail. 2021;8(1):605-14.

199. Jensen MK, et al. Intakes of whole grains, bran, and germ and the risk of coronary heart disease in men. American Journal of Clinical Nutrition. 2004;80(6):1492-9.

200. Jin JL, et al. Impact of free fatty acids on prognosis in coronary artery disease patients under different glucose metabolism status. Cardiovasc Diabetol. 2019;18(1):134.

201. Juanola-Falgarona M, et al. Dietary intake of vitamin K is inversely associated with mortality risk. Journal of nutrition. 2014;144(5):743-50.

202. Juul F, et al. Ultra-Processed Foods and Incident Cardiovascular Disease in the Framingham Offspring Study. Journal of the American College of Cardiology. 2021;77(12):1520-31.

203. Kanbay M, et al. Relationship between serum magnesium levels and cardiovascular events in chronic kidney disease patients. Am J Nephrol. 2012;36(3):228-37.

204. Kant AK, Graubard BI. A prospective study of Water intake and subsequent risk of all-cause mortality in a national cohort. American Journal of Clinical Nutrition. 2017;105(1):212-20.

205. Kant AK, Graubard BI. A prospective study of frequency of eating restaurant prepared meals and subsequent 9-year risk of all-cause and cardiometabolic mortality in US adults. PLoS ONE. 2018;13(1).

206. Karppi J, et al. Serum β-carotene and the risk of sudden cardiac death in men: A population-based follow-up study. Atherosclerosis. 2013;226(1):172-7.

207. Karppi J, et al. Serum lycopene decreases the risk of stroke in men; A population-based follow-up study. Neurology. 2012;79(15):1540-7.

208. Kelemen LE, et al. Associations of dietary protein with disease and mortality in a prospective study of postmenopausal women. Am J Epidemiol. 2005;161(3):239-49.

209. Kelly RK, et al. Associations between types and sources of dietary carbohydrates and cardiovascular disease risk: a prospective cohort study of UK Biobank participants. BMC Med. 2023;21(1):34.

210. Kershaw KN, et al. Quantifying the contributions of behavioral and biological risk factors to socioeconomic disparities in coronary heart disease incidence: The MORGEN study. European Journal of Epidemiology. 2013;28(10):807-14.

211. Khaw KT, et al. Relation between plasma ascorbic acid and mortality in men and women in EPIC-Norfolk prospective study: a prospective population study. European Prospective Investigation into Cancer and Nutrition. Lancet. 2001;357(9257):657-63.

212. Khaw KT, et al. Serum 25-hydroxyvitamin D, mortality, and incident cardiovascular disease, respiratory disease, cancers, and fractures: A 13-y prospective population study. American Journal of Clinical Nutrition. 2014;100(5):1361-70.

213. Khawaja O, et al. Plasma free fatty acids and risk of stroke in the Cardiovascular Health Study. Int J Stroke. 2014;9(7):917-20.

214. Kiage JN, et al. Intake of trans fat and all-cause mortality in the Reasons for Geographical and Racial Differences in Stroke (REGARDS) cohort. Am J Clin Nutr. 2013;97(5):1121-8.

215. Kiage JN, et al. Intake of polyunsaturated fat in relation to mortality among statin users and non-users in the Southern Community Cohort Study. Nutrition, Metabolism and Cardiovascular Diseases. 2015;25(11):1016-24.

216. Kim HN, et al. Serum folate and vitamin B(12) levels are not associated with the incidence risk of atherosclerotic events over 12 years: the Korean Genome and Epidemiology Study. Nutr Res. 2019;63:34-41.

217. Kim SA, et al. Coffee Consumption and the Risk of All-Cause and Cause-Specific Mortality in the Korean Population. Journal of the Academy of Nutrition and Dietetics. 2021;121(11):2221-32.

218. King DE, Xiang J. A relationship between mortality and eating breakfast and fiber. Journal of the American Board of Family Medicine. 2021;34(4):678-87.

219. Klag MJ, et al. Coffee intake and coronary heart disease. Ann Epidemiol. 1994;4(6):425-33.

220. Kodjoe E. Low sodium intake and cardiovascular disease mortality among adults with hypertension. International Journal of Cardiology: Cardiovascular Risk and Prevention. 2022;15.

221. Kojima G, et al. Low dietary vitamin d predicts 34-year incident stroke: The honolulu heart program. Stroke. 2012;43(8):2163-7.

222. Kondo I, et al. Consumption of dairy products and death from cardiovascular disease in the Japanese General Population: The NIPPON DATA80. Journal of Epidemiology. 2013;23(1):47-54.

223. Kumakura H, et al. Eicosapentaenoic Acid Level Predicts Long-Term Survival and Cardiovascular or Limb Event in Peripheral Arterial Disease. Ann Vasc Dis. 2024;17(2):135-41.

224. Kuwamura Y, et al. Altered Serum n-6 Polyunsaturated Fatty Acid Profile and Risks of Mortality and Cardiovascular Events in a Cohort of Hemodialysis Patients. J Ren Nutr. 2018;28(1):54-63.

225. Kwon YJ, et al. Association between dietary sodium, potassium, and the sodium-to-potassium ratio and mortality: A 10-year analysis. Front Nutr. 2022;9:1053585.

226. Kwon YJ, et al. Association Between Dietary Fiber Intake and All-Cause and Cardiovascular Mortality in Middle Aged and Elderly Adults With Chronic Kidney Disease. Front Nutr. 2022;9:863391.

227. Lagström H, et al. Diet quality as a predictor of cardiometabolic disease-free life expectancy: The Whitehall II cohort study. American Journal of Clinical Nutrition. 2020;111(4):787-94.

228. Lapidus L, et al. Dietary habits in relation to incidence of cardiovascular disease and death in women: A 12-year follow-up of participants in the population study of women in Gothenburg, Sweden. American Journal of Clinical Nutrition. 1986;44(4):444-8.

229. Larsson SC, et al. Sweetened beverage consumption is associated with increased risk of stroke in women and men. Journal of Nutrition. 2014;144(6):856-60.

230. Larsson SC, et al. Primary prevention of stroke by a healthy lifestyle in a high-risk group. Neurology. 2015;84(22):2224-8.

231. Larsson SC, Wolk A. Dietary fiber intake is inversely associated with stroke incidence in healthy Swedish adults. Journal of Nutrition. 2014;144(12):1952-5.

232. Larsson SC, Wolk A. Potato consumption and risk of cardiovascular disease: 2 prospective cohort studies. American Journal of Clinical Nutrition. 2016;104(5):1245-52.

233. Lee CH, et al. Dietary intake of anti-oxidant vitamins A, C, and E is inversely associated with adverse cardiovascular outcomes in Chinese—A 22-years population-based prospective study. Nutrients. 2018;10(11).

234. Lee MS, et al. A simple food quality index predicts mortality in Elderly Taiwanese. Journal of Nutrition, Health and Aging. 2011;15(10):815-21.

235. Leurs LJ, et al. Total fluid and specific beverage intake and mortality due to IHD and stroke in the Netherlands Cohort Study. British Journal of Nutrition. 2010;104(8):1212-21.

236. Levitan EB, et al. Coffee consumption and incidence of heart failure in women. Circulation: Heart Failure. 2011;4(4):414-8.

237. Levitan EB, et al. Dietary glycemic index, dietary glycemic load, and cardiovascular disease in middle-aged and older Swedish men. Am J Clin Nutr. 2007;85(6):1521-6.

238. Levitan EB, et al. Dietary glycemic index, dietary glycemic load, and incidence of heart failure events: a prospective study of middle-aged and elderly women. J Am Coll Nutr. 2010;29(1):65-71.

239. Levitan EB, et al. Consistency with the DASH diet and incidence of heart failure. Archives of Internal Medicine. 2009;169(9):851-7.

240. Levitan EB, et al. Fatty fish, marine-3 fatty acids and incidence of heart failure. European Journal of Clinical Nutrition. 2010;64(6):587-94.

241. Levitan EB, et al. Fatty fish, marine omega-3 fatty acids and incidence of heart failure. Eur J Clin Nutr. 2010;64(6):587-94.

242. Li M, et al. Effects of tryptophan, serotonin, and kynurenine on ischemic heart diseases and its risk factors: a Mendelian Randomization study. European Journal of Clinical Nutrition. 2020;74(4):613-21.

243. Li Y, et al. Saturated Fats Compared with Unsaturated Fats and Sources of Carbohydrates in Relation to Risk of Coronary Heart Disease A Prospective Cohort Study. Journal of the American College of Cardiology. 2015;66(14):1538-48.

244. Li Y, et al. Plant-based diets and the incidence of cardiovascular disease: The Million Veteran Program. BMJ Nutrition, Prevention and Health. 2023;6(2):212-20.

245. Liang J, et al. Utilization of plant-based foods for effective prevention of chronic diseases: a longitudinal cohort study. NPJ Sci Food. 2024;8(1):113.

246. Liebeskind DS, et al. The coffee paradox in stroke: Increased consumption linked with fewer strokes. Nutritional Neuroscience. 2016;19(9):406-13.

247. Lin B, et al. Associations of serum carotenoids with all-cause and cardiovascular mortality in adults with MAFLD. Nutrition, Metabolism and Cardiovascular Diseases. 2024;34(10):2315-24.

248. Lin HP, et al. Dietary cholesterol, lipid levels, and cardiovascular risk among adults with diabetes or impaired fasting glucose in the Framingham Offspring study. Nutrients. 2018;10(6).

249. Liu AH, et al. Relationship of dietary nitrate intake from vegetables with cardiovascular disease mortality: a prospective study in a cohort of older Australians. European Journal of Nutrition. 2019;58(7):2741-53.

250. Liu D, et al. Markers of Iron Metabolism and Stroke Risk: Cross-Sectional and Longitudinal Findings from the China Health and Nutrition Survey (CHNS). Iran J Public Health. 2022;51(1):115-23.

251. Liu L, et al. Serum 25-hydroxyvitamin D, frailty, and mortality among the Chinese oldest old: Results from the CLHLS study. Nutrition, Metabolism and Cardiovascular Diseases. 2021;31(9):2707-15.

252. Liu S, et al. Intake of vegetables rich in carotenoids and risk of coronary heart disease in men: the Physicians' Health Study. International journal of epidemiology. 2001;30(1):130-5.

253. Liu S, et al. Fruit and vegetable intake and risk of cardiovascular disease: the Women's Health Study. American journal of clinical nutrition. 2000;72(4):922-8.

254. Liu S, et al. Whole grain consumption and risk of ischemic stroke in women: A prospective study. JAMA. 2000;284(12):1534-40.

255. Liu S, et al. Whole-grain consumption and risk of coronary heart disease: Results from the Nurses' Health Study. American Journal of Clinical Nutrition. 1999;70(3):412-9.

256. Liu S, et al. A prospective study of dietary glycemic load, carbohydrate intake, and risk of coronary heart disease in US women. American Journal of Clinical Nutrition. 2000;71(6):1455-61.

257. Liu Y, et al. Changes in fatty acid intake and subsequent risk of all-cause and cause-specific mortality in males and females: a prospective cohort study. American Journal of Clinical Nutrition. 2025;121(1):141-50.

258. Liu Z, et al. Dietary Patterns and Long-Term Outcomes in Patients with NAFLD: A Prospective Analysis of 128,695 UK Biobank Participants. Nutrients. 2023;15(2).

259. Livingstone KM, et al. Nineteen-Year Associations between Three Diet Quality Indices and All-Cause and Cardiovascular Disease Mortality: The Australian Diabetes, Obesity, and Lifestyle Study. J Nutr. 2022;152(3):805-15.

260. Lopez-Garcia E, et al. Coffee consumption and mortality in women with cardiovascular disease. American Journal of Clinical Nutrition. 2011;94(1):218-24.

261. Lopez-Pineda A, et al. Lifestyle Habits and Risk of Cardiovascular Mortality in Menopausal Women with Cardiovascular Risk Factors: A Retrospective Cohort Study. Journal of Cardiovascular Development and Disease. 2024;11(9).

262. Lv J, et al. Adherence to Healthy Lifestyle and Cardiovascular Diseases in the Chinese Population. J Am Coll Cardiol. 2017;69(9):1116-25.

263. Ma T, et al. Use of fish oil and mortality of patients with cardiometabolic multimorbidity: A prospective study of UK biobank. Nutrition, Metabolism and Cardiovascular Diseases. 2022;32(12):2751-9.

264. Mao Y, et al. Association of serum 25-hydroxyvitamin d concentrations with all-cause and cause-specific mortality among individuals with depression: A cohort study. J Affect Disord. 2024;352:10-8.

265. Matheson EM, et al. Shellfish Consumption and Risk of Coronary Heart Disease. Journal of the American Dietetic Association. 2009;109(8):1422-6.

266. Mazidi M, et al. Egg Consumption and Risk of Total and Cause-Specific Mortality: An Individual-Based Cohort Study and Pooling Prospective Studies on Behalf of the Lipid and Blood Pressure Meta-analysis Collaboration (LBPMC) Group. Journal of the American College of Nutrition. 2019;38(6):552-63.

267. Mazidi M, et al. Association of types of dietary fats and all-cause and cause-specific mortality: A prospective cohort study and meta-analysis of prospective studies with 1,164,029 participants. Clin Nutr. 2020;39(12):3677-86.

268. McKay GJ, et al. Association of low plasma antioxidant levels with all-cause mortality and coronary events in healthy middle-aged men from France and Northern Ireland in the PRIME study. European Journal of Nutrition. 2021;60(5):2631-41.

269. Meinitzer A, et al. Asymmetrical dimethylarginine independently predicts total and cardiovascular mortality in individuals with angiographic coronary artery disease (the Ludwigshafen Risk and Cardiovascular Health study). Clin Chem. 2007;53(2):273-83.

270. Meishuo O, et al. Association between Dietary Manganese Intake and Mortality from Cardiovascular Disease in Japanese Population: The Japan Collaborative Cohort Study. J Atheroscler Thromb. 2022;29(10):1432-47.

271. Melamed ML, et al. 25-hydroxyvitamin D levels and the risk of mortality in the general population. Arch Intern Med. 2008;168(15):1629-37.

272. Micha R, et al. Association Between Dietary Factors and Mortality From Heart Disease, Stroke, and Type 2 Diabetes in the United States. Jama. 2017;317(9):912-24.

273. Michaëlsson K, et al. Combined associations of body mass index and adherence to a Mediterranean-like diet with all-cause and cardiovascular mortality: A cohort study. PLoS Medicine. 2020;17(9).

274. Michos ED, et al. Vitamin D and subclinical cerebrovascular disease: the Atherosclerosis Risk in Communities brain magnetic resonance imaging study. JAMA Neurol. 2014;71(7):863-71.

275. Mihrshahi S, et al. Vegetarian diet and all-cause mortality: Evidence from a large population-based Australian cohort - the 45 and Up Study. Preventive Medicine. 2017;97:1-7.

276. Mizuiri S, et al. Absolute iron deficiency, coronary artery calcification and cardiovascular mortality in maintenance haemodialysis patients. Nephrology (Carlton). 2024;29(7):415-21.

277. Mohseni G, et al. Egg consumption and risk of cardiovascular disease: a PERSIAN cohort-based study. BMC Cardiovascular Disorders. 2023;23(1).

278. Montonen J, et al. Fish consumption and the incidence of cerebrovascular disease. British Journal of Nutrition. 2009;102(5):750-6.

279. Mosharraf S, et al. Impact of the components of Mediterranean nutrition regimen on long-term prognosis of diabetic patients with coronary artery disease. ARYA Atherosclerosis. 2013;9(6):337-42.

280. Mossavar-Rahmani Y, et al. Artificially Sweetened Beverages and Stroke, Coronary Heart Disease, and All-Cause Mortality in the Women's Health Initiative. Stroke. 2019;50(3):555-62.

281. Mostofsky E, et al. Chocolate intake and incidence of heart failure a population-based prospective study of middle-aged and elderly women. Circulation: Heart Failure. 2010;3(5):612-6.

282. Mullee A, et al. Association Between Soft Drink Consumption and Mortality in 10 European Countries. JAMA Intern Med. 2019;179(11):1479-90.

283. Muñoz-Bravo C, et al. Association between serum copper levels and risk of cardiovascular disease: A nested case-control study in the PREDIMED trial. Nutrition, Metabolism and Cardiovascular Diseases. 2023;33(11):2199-208.

284. Mursu J, et al. Diet quality indexes and mortality in postmenopausal women: the Iowa Women's Health Study. Am J Clin Nutr. 2013;98(2):444-53.

285. Mursu J, et al. Flavonoid intake and the risk of ischaemic stroke and CVD mortality in middle-aged Finnish men: The Kuopio Ischaemic Heart Disease Risk Factor Study. British Journal of Nutrition. 2008;100(4):890-5.

286. Musicus AA, et al. Health and environmental impacts of plant-rich dietary patterns: a US prospective cohort study. The Lancet Planetary Health. 2022;6(11):e892-e900.

287. Myint PK, et al. Combined effect of health behaviours and risk of first ever stroke in 20 040 men and women over 11 years' follow-up in Norfolk cohort of European Prospective Investigation of Cancer (EPIC Norfolk): Prospective population study. BMJ (Online). 2009;338(7695).

288. Myint PK, et al. Plasma vitamin C concentrations predict risk of incident stroke over 10 y in 20 649 participants of the European Prospective Investigation into Cancer Norfolk prospective population study. Am J Clin Nutr. 2008;87(1):64-9.

289. Myint PK, et al. Habitual fish consumption and risk of incident stroke: The European Prospective Investigation into Cancer (EPIC)-Norfolk prospective population study. Public Health Nutrition. 2006;9(7):882-8.

290. Nakamura T, et al. Serum fatty acid levels, dietary style and coronary heart disease in three neighbouring areas in Japan: the Kumihama study. Br J Nutr. 2003;89(2):267-72.

291. Nargesi AA, et al. Contribution of vitamin D deficiency to the risk of coronary heart disease in subjects with essential hypertension. Atherosclerosis. 2016;244:165-71.

292. Naves-Díaz M, et al. Calcium, phosphorus, PTH and death rates in a large sample of dialysis patients from Latin America. The CORES Study. Nephrol Dial Transplant. 2011;26(6):1938-47.

293. Nettleton JA, et al. Dietary patterns and incident cardiovascular disease in the Multi-Ethnic Study of Atherosclerosis. Am J Clin Nutr. 2009;90(3):647-54.

294. Nettleton JA, et al. Incident Heart Failure Is Associated with Lower Whole-Grain Intake and Greater High-Fat Dairy and Egg Intake in the Atherosclerosis Risk in Communities (ARIC) Study. Journal of the American Dietetic Association. 2008;108(11):1881-7.

295. Neuhouser ML, et al. Associations of Biomarker-Calibrated Healthy Eating Index-2010 Scores with Chronic Disease Risk and Their Dependency on Energy Intake and Body Mass Index in Postmenopausal Women. J Nutr. 2023;152(12):2808-17.

296. Nickel DV, et al. Healthy food diversity and the risk of major chronic diseases in the EPIC-Potsdam study. Scientific reports. 2024;14(1):28635.

297. Nilsson LM, et al. A traditional Sami diet score as a determinant of mortality in a general northern Swedish population. Int J Circumpolar Health. 2012;71(0):1-12.

298. Nomura SO, et al. Free fatty acids and heart failure in the Multi-Ethnic Study of Atherosclerosis (MESA). Journal of Clinical Lipidology. 2021;15(4):608-17.

299. O'Donnell MJ, et al. Urinary sodium and potassium excretion and risk of cardiovascular events. JAMA. 2011;306(20):2229-38.

300. Odegaard AO, et al. Combined lifestyle factors and cardiovascular disease mortality in Chinese men and women: the Singapore Chinese health study. Circulation. 2011;124(25):2847-54.

301. Oh K, et al. Carbohydrate intake, glycemic index, glycemic load, and dietary fiber in relation to risk of stroke in women. Am J Epidemiol. 2005;161(2):161-9.

302. Ohira T, et al. Serum and dietary magnesium and risk of ischemic stroke: the Atherosclerosis Risk in Communities Study. Am J Epidemiol. 2009;169(12):1437-44.

303. Oomen CM, et al. Arginine intake and risk of coronary heart disease mortality in elderly men. Arterioscler Thromb Vasc Biol. 2000;20(9):2134-9.

304. Orencia AJ, et al. Fish consumption and stroke in men: 30-year findings of the Chicago Western Electric study. Stroke. 1996;27(2):204-9.

305. Owen AJ, et al. Polyunsaturated fatty acid intake and risk of cardiovascular mortality in a low fish-consuming population: a prospective cohort analysis. European Journal of Nutrition. 2016;55(4):1605-13.

306. Pacheco LS, et al. Sugar-sweetened beverage intake and cardiovascular disease risk in the california teachers study. Journal of the American Heart Association. 2020;9(10).

307. Pacheco LS, et al. Association Between Sugar-Sweetened Beverage Intake and Mortality Risk in Women: The California Teachers Study. J Acad Nutr Diet. 2022;122(2):320-33.e6.

308. Pacheco LS, et al. Avocado Consumption and Risk of Cardiovascular Disease in US Adults. Journal of the American Heart Association. 2022;11(7).

309. Paganini-Hill A, et al. Antioxidant vitamin intake and mortality: the Leisure World Cohort Study. Am J Epidemiol. 2015;181(2):120-6.

310. Palatini P, et al. Coffee consumption and risk of cardiovascular events in hypertensive patients. Results from the HARVEST. International Journal of Cardiology. 2016;212:131-7.

311. Palmer CR, et al. Association between vitamin K(1) intake and mortality in the Danish Diet, Cancer, and Health cohort. Eur J Epidemiol. 2021;36(10):1005-14.

312. Pan J, et al. Dietary Potassium and Clinical Outcomes among Patients on Peritoneal Dialysis. Nutrients. 2023;15(19).

313. Pan XF, et al. Seventeen-year associations between diet quality defined by the health star rating and mortality in australians: The australian diabetes, obesity and lifestyle study (ausdiab). Current Developments in Nutrition. 2020;4(11).

314. Panagiotakos DB, et al. Five-year incidence of cardiovascular disease and its predictors in Greece: The ATTICA study. Vascular Medicine. 2008;13(2):113-21.

315. Panagiotakos DB, et al. The effect of clinical characteristics and dietary habits on the relationship between education status and 5-year incidence of cardiovascular disease: The ATTICA study. European Journal of Nutrition. 2008;47(5):258-65.

316. Pant A, et al. Ultra-processed foods and incident cardiovascular disease and hypertension in middle-aged women. European Journal of Nutrition. 2024;63(3):713-25.

317. Papanikolaou Y, Fulgoni VL, 3rd. Dairy Food Consumption Is Associated with Reduced Risk of Heart Disease Mortality, but Not All-Cause and Cancer Mortality in US Adults. Nutrients. 2023;15(2).

318. Papier K, et al. Vegetarian diets and risk of hospitalisation or death with diabetes in British adults: results from the EPIC-Oxford study. Nutrition and Diabetes. 2019;9(1).

319. Park S, et al. Causal effects of serum levels of n-3 or n-6 polyunsaturated fatty acids on coronary artery disease: mendelian randomization study. Nutrients. 2021;13(5).

320. Park SY, et al. Association of Coffee Consumption With Total and Cause-Specific Mortality Among Nonwhite Populations. Ann Intern Med. 2017;167(4):228-35.

321. Park Y, et al. Dietary fiber intake and mortality in the NIH-AARP diet and health study. Archives of Internal Medicine. 2011;171(12):1061-8.

322. Park YM, et al. Mediterranean diet and mortality risk in metabolically healthy obese and metabolically unhealthy obese phenotypes. International Journal of Obesity. 2016;40(10):1541-9.

323. Parker HW, et al. Modified-Weight Healthy Eating Index-2015 Scores Are More Strongly Associated With Mortality Risk Than Standard Scores. Journal of the Academy of Nutrition and Dietetics. 2024;124(3):331-45.

324. Patel YR, et al. Mediterranean, dash, and alternate healthy eating index dietary patterns and risk of death in the physicians' health study. Nutrients. 2021;13(6).

325. Patterson E, et al. Association between dairy food consumption and risk of myocardial infarction in women differs by type of dairy food. J Nutr. 2013;143(1):74-9.

326. Peila R, et al. Healthy Lifestyle Index and Risk of Cardiovascular Disease Among Postmenopausal Women With Normal Body Mass Index. Journal of the American Heart Association. 2023;12(12).

327. Petrakis I, et al. Cardiovascular and All-Cause Mortality Is Affected by Serum Magnesium and Diet Pattern in a Cohort of Dialysis Patients. Journal of Clinical Medicine. 2024;13(14).

328. Pfister R, et al. Estimated urinary sodium excretion and risk of heart failure in men and women in the EPIC-Norfolk study. European Journal of Heart Failure. 2014;16(4):394-402.

329. Pfister R, et al. Plasma vitamin C predicts incident heart failure in men and women in European Prospective Investigation into Cancer and Nutrition-Norfolk prospective study. American Heart Journal. 2011;162(2):246-53.

330. Pilz S, et al. Vitamin D and mortality in older men and women. Clin Endocrinol (Oxf). 2009;71(5):666-72.

331. Pirro M, et al. Plasma free fatty acid levels and the risk of ischemic heart disease in men: Prospective results from the Québec cardiovascular study. Atherosclerosis. 2002;160(2):377-84.

332. Porto CM, et al. Association between vitamin D deficiency and heart failure risk in the elderly. ESC Heart Fail. 2018;5(1):63-74.

333. Praagman J, et al. Dairy products and the risk of stroke and coronary heart disease: the Rotterdam Study. European Journal of Nutrition. 2015;54(6):981-90.

334. Preis SR, et al. Dietary protein and risk of ischemic heart disease in middle-aged men. American Journal of Clinical Nutrition. 2010;92(5):1265-72.

335. Prentice RL, et al. Four-Day Food Record Macronutrient Intake, with and Without Biomarker Calibration, and Chronic Disease Risk in Postmenopausal Women. American Journal of Epidemiology. 2022;191(6):1061-70.

336. Qin C, et al. Dietary patterns and cardiometabolic diseases in 0.5 million Chinese adults: a 10-year cohort study. Nutrition Journal. 2021;20(1).

337. Rassy N, et al. Association of Healthy Lifestyle Factors and Obesity-Related Diseases in Adults in the UK. JAMA Network Open. 2023;6(5):E2314741.

338. Rauber F, et al. Implications of food ultra-processing on cardiovascular risk considering plant origin foods: an analysis of the UK Biobank cohort. The Lancet Regional Health - Europe. 2024;43.

339. Rimm EB, et al. Relation between intake of flavonoids and risk for coronary heart disease in male health professionals. Ann Intern Med. 1996;125(5):384-9.

340. Rosner SA, et al. Coffee consumption and risk of myocardial infarction among older Swedish women. American Journal of Epidemiology. 2007;165(3):288-93.

341. Rossi M, et al. Relation of dietary glycemic load with ischemic and hemorrhagic stroke: a cohort study in Greece and a meta-analysis. European Journal of Nutrition. 2015;54(2):215-22.

342. Sawicki CM, et al. Planetary health diet and cardiovascular disease: results from three large prospective cohort studies in the USA. The Lancet Planetary Health. 2024;8(9):e666-e74.

343. Sawicki CM, et al. Metabolite Profiles of Plant-Based Diets and Cardiometabolic Risk in the Mediators of Atherosclerosis in South Asians Living in America Study. J Nutr. 2024;154(8):2501-13.

344. Sheehy S, et al. High Consumption of Red Meat Is Associated with Excess Mortality among African-American Women. Journal of Nutrition. 2020;150(12):3249-58.

345. Shi Y, et al. Eastern Diet - a healthful dietary pattern from Eastern China: Its characteristics and relation to adiposity, cardiometabolic diseases, mortality, and gut microbiota. 2024.

346. Shin J, et al. Effects of lifestyle-related factors on ischemic heart disease according to body mass index and fasting blood glucose levels in Korean adults. PLoS ONE. 2019;14(5).

347. Shivappa N, et al. Inflammatory potential of diet and all-cause, cardiovascular, and cancer mortality in National Health and Nutrition Examination Survey III Study. European Journal of Nutrition. 2017;56(2):683-92.

348. Simon TG, et al. Coffee consumption is not associated with prevalent subclinical cardiovascular disease (CVD) or the risk of CVD events, in nonalcoholic fatty liver disease: results from the multi-ethnic study of atherosclerosis. Metabolism: Clinical and Experimental. 2017;75:1-5.

349. Singh-Manoux A, et al. Clinical, socioeconomic, and behavioural factors at age 50 years and risk of cardiometabolic multimorbidity and mortality: A cohort study. PLoS Medicine. 2018;15(5).

350. Sjögren P, et al. Mediterranean and carbohydrate-restricted diets and mortality among elderly men: a cohort study in Sweden. Am J Clin Nutr. 2010;92(4):967-74.

351. Sonestedt E, et al. Dairy Consumption, Lactase Persistence, and Mortality Risk in a Cohort From Southern Sweden. Front Nutr. 2021;8:779034.

352. Song Y, et al. Association between dietary branched-chain amino acids and multiple chronic conditions among older adults in Chinese communities. Nutr Metab (Lond). 2024;21(1):56.

353. Soohoo M, et al. Association of serum vitamin B12 and folate with mortality in incident hemodialysis patients. Nephrol Dial Transplant. 2017;32(6):1024-32.

354. Sotos-Prieto M, et al. Association between the Mediterranean lifestyle, metabolic syndrome and mortality: a whole-country cohort in Spain. Cardiovasc Diabetol. 2021;20(1):5.

355. Stampfer MJ, et al. Primary prevention of coronary heart disease in women through diet and lifestyle. New England Journal of Medicine. 2000;343(1):16-22.

356. Stewart RA, et al. Dietary patterns and the risk of major adverse cardiovascular events in a global study of high-risk patients with stable coronary heart disease. European heart journal. 2016;37(25):1993-2001.

357. Strengers JG, et al. The association of the Mediterranean diet with heart failure risk in a Dutch population. Nutrition, Metabolism and Cardiovascular Diseases. 2021;31(1):60-6.

358. Sun L, et al. Associations of erythrocyte polyunsaturated fatty acids with incidence of stroke and stroke types in adult Chinese: a prospective study of over 8000 individuals. Eur J Nutr. 2022;61(6):3235-46.

359. Sun T, et al. Artificial sweeteners and risk of incident cardiovascular disease and mortality: evidence from UK Biobank. Cardiovasc Diabetol. 2024;23(1):233.

360. Takata Y, et al. Fish intake and risks of total and cause-specific mortality in 2 population-based cohort studies of 134,296 men and women. American Journal of Epidemiology. 2013;178(1):46-57.

361. Taveira TH, et al. Relation of Magnesium Intake With Cardiac Function and Heart Failure Hospitalizations in Black Adults: The Jackson Heart Study. Circ Heart Fail. 2016;9(4):e002698.

362. Tertsunen HM, et al. Healthy Nordic diet and risk of disease death among men: the Kuopio Ischaemic Heart Disease Risk Factor Study. European Journal of Nutrition. 2020;59(8):3545-53.

363. Thomas GN, et al. Vitamin D levels predict all-cause and cardiovascular disease mortality in subjects with the metabolic syndrome: The Ludwigshafen risk and cardiovascular health (LURIC) study. Diabetes Care. 2012;35(5):1158-64.

364. Toda M, et al. Relationship between daily eating habits and occurrence of stroke in the O City Cohort I survey: a 26-year follow-up of residents in rural Japan. J Rural Med. 2025;20(1):28-38.

365. Tong TYN, et al. Risks of ischaemic heart disease and stroke in meat eaters, fish eaters, and vegetarians over 18 years of follow-up: Results from the prospective EPIC-Oxford study. The BMJ. 2019;366.

366. Toussaint ND, et al. Relationship Between Urinary Phosphate and All-Cause and Cardiovascular Mortality in a National Population-Based Longitudinal Cohort Study. Journal of renal nutrition : the official journal of the Council on Renal Nutrition of the National Kidney Foundation. 2022;32(5):510-9.

367. Treskes RW, et al. Use of sugar in coffee and tea and long-term risk of mortality in older adult Danish men: 32 years of follow-up from a prospective cohort study. PLoS ONE. 2023;18(10 October).

368. Tverdal A, et al. Coffee consumption and death from coronary heart disease in middle aged Norwegian men and women. Bmj. 1990;300(6724):566-9.

369. Twum F, et al. High red blood cell folate is associated with an increased risk of diabetes death among a hypertensive cohort. Nutrition Research. 2024;126:204-14.

370. Ushula TW, et al. Dietary patterns explaining variations in blood biomarkers in young adults are associated with the 30-year predicted cardiovascular disease risks in midlife: A follow-up study. Nutrition, Metabolism and Cardiovascular Diseases. 2023;33(5):1007-18.

371. Vacek JL, et al. Vitamin D deficiency and supplementation and relation to cardiovascular health. Am J Cardiol. 2012;109(3):359-63.

372. Valenzuela PL, et al. Obesity, cardiovascular risk, and lifestyle: cross-sectional and prospective analyses in a nationwide Spanish cohort. European Journal of Preventive Cardiology. 2023;30(14):1493-501.

373. Van Dam RM, et al. Dietary glycemic index in relation to metabolic risk factors and incidence of coronary heart disease: The Zutphen elderly study. European Journal of Clinical Nutrition. 2000;54(9):726-31.

374. van den Brandt PA, Schouten LJ. Relationship of tree nut, peanut and peanut butter intake with total and cause-specific mortality: A cohort study and meta-analysis. International Journal of Epidemiology. 2015;44(3):1038-49.

375. Vega-Cabello V, et al. Plant-Based Diets and Risk of Multimorbidity: The Health and Retirement Study. Journal of Nutrition. 2024;154(7):2264-72.

376. Vermeulen EA, et al. Magnesium intake and vascular structure and function: the Hoorn Study. European Journal of Nutrition. 2022;61(2):653-64.

377. Virtanen HEK, et al. Dietary proteins and protein sources and risk of death: The Kuopio ischaemic heart disease risk factor study. American Journal of Clinical Nutrition. 2019;109(5):1462-71.

378. Virtanen JK, et al. Serum n-6 polyunsaturated fatty acids and risk of death: The Kuopio Ischaemic Heart Disease Risk Factor Study. American Journal of Clinical Nutrition. 2018;107(3):427-35.

379. Vissers LET, et al. Fatty acids from dairy and meat and their association with risk of coronary heart disease. European Journal of Nutrition. 2019;58(7):2639-47.

380. Voortman T, et al. Adherence to the 2015 Dutch dietary guidelines and risk of non-communicable diseases and mortality in the Rotterdam Study. Eur J Epidemiol. 2017;32(11):993-1005.

381. Vuori MA, et al. 24-h urinary sodium excretion and the risk of adverse outcomes. Ann Med. 2020;52(8):488-96.

382. Wallström P, et al. Dietary fiber and saturated fat intake associations with cardiovascular disease differ by sex in the Malmö diet and cancer cohort: A prospective study. PLoS ONE. 2012;7(2).

383. Wang DD, et al. Red Meat Intake and the Risk of Cardiovascular Diseases: A Prospective Cohort Study in the Million Veteran Program. Journal of Nutrition. 2024;154(3):886-95.

384. Wang J, et al. Ultra-processed food, genetic risk, and the risk of cardiometabolic diseases and cardiometabolic multimorbidity: A prospective study. Nutrition, Metabolism and Cardiovascular Diseases. 2024;34(12):2799-806.

385. Wang K, et al. Association of caffeine consumption with all-cause and cause-specific mortality in adult Americans with hypertension. Food Sci Nutr. 2024;12(6):4185-95.

386. Wang L, et al. Consumption of ultra-processed foods and all-cause and cause-specific mortality in the Southern Community Cohort Study. Clinical Nutrition. 2023;42(10):1866-74.

387. Wang P, et al. Socioeconomic Status, Diet, and Behavioral Factors and Cardiometabolic Diseases and Mortality. JAMA network open. 2024;7(12):e2451837.

388. Wang P, et al. Night eating in timing, frequency, and food quality and risks of all-cause, cancer, and diabetes mortality: findings from national health and nutrition examination survey. Nutrition and Diabetes. 2024;14(1).

389. Wang Q, et al. Association of Life's Simple 7 lifestyle metric with cardiometabolic disease-free life expectancy in older British men. Commun Med (Lond). 2024;4(1):104.

390. Wang Q, et al. Prospective associations between diet quality, dietary components, and risk of cardiometabolic multimorbidity in older British men. European Journal of Nutrition. 2023;62(7):2793-804.

391. Wang S, et al. Decreased risk of all-cause and heart-specific mortality is associated with low-fat or skimmed milk consumption compared with whole milk intake: A cohort study. Clinical Nutrition. 2021;40(11):5568-75.

392. Wang T, et al. Age-specific modifiable risk factor profiles for cardiovascular disease and all-cause mortality: a nationwide, population-based, prospective cohort study. Lancet Reg Health West Pac. 2021;17:100277.

393. Wang Y, et al. Unfavorable Dietary Quality Contributes to Elevated Risk of Ischemic Stroke among Residents in Southwest China: Based on the Chinese Diet Balance Index 2016 (DBI-16). Nutrients. 2022;14(3).

394. Wang Y, et al. Lifestyle factors in relation to heart failure among Finnish men and women. Circulation: Heart Failure. 2011;4(5):607-12.

395. Wannamethee G, et al. Serum sodium concentration and risk of stroke in middle-aged males. Journal of Hypertension. 1994;12(8):971-9.

396. Wannamethee SG, et al. Plasma vitamin C, but not vitamin E, is associated with reduced risk of heart failure in older men. Circulation: Heart Failure. 2013;6(4):647-54.

397. Ward HA, et al. Fibre intake in relation to serum total cholesterol levels and CHD risk: A comparison of dietary assessment methods. European Journal of Clinical Nutrition. 2012;66(3):296-304.

398. Warfa K, et al. Association between sucrose intake and acute coronary event risk and effect modification by lifestyle factors: Malmö Diet and Cancer Cohort Study. Br J Nutr. 2016;116(9):1611-20.

399. Welsh CE, et al. Urinary Sodium Excretion, Blood Pressure, and Risk of Future Cardiovascular Disease and Mortality in Subjects Without Prior Cardiovascular Disease. Hypertension (Dallas, Tex : 1979). 2019;73(6):1202-9.

400. Wen X, et al. Plasma lipidomic markers of diet quality are associated with incident coronary heart disease in American Indian adults: the Strong Heart Family Study. American Journal of Clinical Nutrition. 2024;119(3):748-55.

401. Wu H, et al. Association between dietary whole grain intake and risk of mortality: two large prospective studies in US men and women. JAMA Intern Med. 2015;175(3):373-84.

402. Wu M, et al. Associations between the inflammatory potential of diets with adherence to plant-based dietary patterns and the risk of new-onset cardiometabolic diseases in Chinese adults: findings from a nation-wide prospective cohort study. Food Funct. 2023;14(19):9018-34.

403. Xia T, et al. Serum iron status is associated with all-cause mortality in metabolic dysfunction-associated steatotic liver disease: a prospective, observational study. Frontiers in Endocrinology. 2024;15.

404. Xiong Z, et al. Nonesterified fatty acids and cardiovascular mortality in elderly men with CKD. Clin J Am Soc Nephrol. 2015;10(4):584-91.

405. Xu H, et al. Modest U-shaped association between dietary acid load and risk of all-cause and cardiovascular mortality in adults. Journal of Nutrition. 2016;146(8):1580-5.

406. Xu M, et al. Ready-to-Eat Cereal Consumption with Total and Cause-Specific Mortality: Prospective Analysis of 367,442 Individuals. Journal of the American College of Nutrition. 2016;35(3):217-23.

407. Xu X, et al. Eating and healthy ageing: a longitudinal study on the association between food consumption, memory loss and its comorbidities. International journal of public health. 2020;65(5):571-82.

408. Xu Z, et al. Diet quality, change in diet quality and risk of incident CVD and diabetes. Public health nutrition. 2020;23(2):329-38.

409. Yan H, et al. Association of seafood consumption with cardiovascular disease among adults in Qingdao, China. Nutr Metab Cardiovasc Dis. 2024;34(3):651-60.

410. Yang J, et al. Coarse Grain Consumption and Risk of Cardiometabolic Diseases: A Prospective Cohort Study of Chinese Adults. Journal of Nutrition. 2022;152(6):1476-86.

411. Yeung SM, et al. Urinary potassium excretion and mortality risk in community-dwelling individuals with and without obesity. American Journal of Clinical Nutrition. 2022;116(3):741-9.

412. Yu D, et al. Dietary carbohydrates, refined grains, glycemic load, and risk of coronary heart disease in Chinese adults. Am J Epidemiol. 2013;178(10):1542-9.

413. Yu D, et al. Fruit and vegetable intake and risk of CHD: Results from prospective cohort studies of Chinese adults in Shanghai. British Journal of Nutrition. 2014;111(2):353-62.

414. Yu D, et al. Dietary glycemic index, glycemic load, and refined carbohydrates are associated with risk of stroke: A prospective cohort study in urban Chinese women. American Journal of Clinical Nutrition. 2016;104(5):1345-51.

415. Yu D, et al. Adherence to dietary guidelines and mortality: a report from prospective cohort studies of 134,000 Chinese adults in urban Shanghai. Am J Clin Nutr. 2014;100(2):693-700.

416. Yu Y, et al. Serum Free Fatty Acids Independently Predict Adverse Outcomes in Acute Heart Failure Patients. Front Cardiovasc Med. 2021;8:761537.

417. Yu Y, et al. Life's Essential 8 and risk of non-communicable chronic diseases: Outcome-wide analyses. Chinese Medical Journal. 2024;137(13):1553-62.

418. Yusuf S, et al. Modifiable risk factors, cardiovascular disease, and mortality in 155 722 individuals from 21 high-income, middle-income, and low-income countries (PURE): a prospective cohort study. The Lancet. 2020;395(10226):795-808.

419. Zhang N, et al. Lifestyle factors and their relative contributions to longitudinal progression of cardio-renal-metabolic multimorbidity: a prospective cohort study. Cardiovascular Diabetology. 2024;23(1).

420. Zhang X, et al. Healthy lifestyle behaviours and all-cause and cardiovascular mortality among 0.9 million Chinese adults. Int J Behav Nutr Phys Act. 2021;18(1):162.

421. Zhang X, et al. Soy food consumption is associated with lower risk of coronary heart disease in Chinese women. Journal of Nutrition. 2003;133(9):2874-8.

422. Zhang Y, et al. Life's essential 8 and mortality in US adults with metabolic dysfunction-associated steatotic liver disease. BMC public health. 2024;24(1):3411.

423. Zhang Y, et al. Consumption of coffee and tea and risk of developing stroke, dementia, and poststroke dementia: A cohort study in the UK Biobank. PLoS Medicine. 2021;18(11).

424. Zhang Y, et al. Cooking oil/fat consumption and deaths from cardiometabolic diseases and other causes: prospective analysis of 521,120 individuals. BMC Medicine. 2021;19(1).

425. Zhao Y, et al. Associations of dietary flavonoids and subclasses with total and cardiovascular mortality among 369,827 older people: The NIH-AARP Diet and Health Study. Atherosclerosis. 2023;365:1-8.

426. Zhong P, et al. Cardiovascular and microvascular outcomes according to vitamin D level and genetic variants among individuals with prediabetes: a prospective study. J Transl Med. 2023;21(1):724.

427. Zhou J, et al. Dietary diversity indices v. dietary guideline-based indices and their associations with non-communicable diseases, overweight and energy intake: evidence from China. Public health nutrition. 2023;26(5):911-33.

428. Zhuang P, et al. Dietary Fats in Relation to Total and Cause-Specific Mortality in a Prospective Cohort of 521 120 Individuals With 16 Years of Follow-Up. Circ Res. 2019;124(5):757-68.

429. Diabetes Nutrition and Complications Trial: adherence to the ADA nutritional recommendations, targets of metabolic control, and onset of diabetes complications. A 7-year, prospective, population-based, observational multicenter study. Journal of Diabetes and its Complications. 2006;20(6):361-6.

430. Afriyie-Gyawu E, et al. Serum folate levels and fatality among diabetic adults: A 15-y follow-up study of a national cohort. Nutrition. 2016;32(4):468-73.

431. Alele JD, et al. Relationship between vitamin D status and incidence of vascular events in the Veterans Affairs Diabetes Trial. Atherosclerosis. 2013;228(2):502-7.

432. Araki SI, et al. Urinary potassium excretion and renal and cardiovascular complications in patients with type 2 diabetes and normal renal function. Clinical Journal of the American Society of Nephrology. 2015;10(12):2152-8.

433. Bener A, et al. Assessment of the Role of Serum 25-Hydroxy Vitamin D Level on Coronary Heart Disease Risk With Stratification Among Patients With Type 2 Diabetes Mellitus. Angiology. 2021;72(1):86-92.

434. Bidel S, et al. Coffee consumption and risk of total and cardiovascular mortality among patients with type 2 diabetes. Diabetologia. 2006;49(11):2618-26.

435. Bonaccio M, et al. Impact of combined healthy lifestyle factors on survival in an adult general population and in high-risk groups: prospective results from the Moli-sani Study. Journal of Internal Medicine. 2019;286(2):207-20.

436. Bonaccio M, et al. Ultraprocessed food consumption is associated with all-cause and cardiovascular mortality in participants with type 2 diabetes independent of diet quality: a prospective observational cohort study. American Journal of Clinical Nutrition. 2023;118(3):627-36.

437. Buziau AM, et al. Total Fermented Dairy Food Intake Is Inversely Associated with Cardiovascular Disease Risk in Women. Journal of Nutrition. 2019;149(10):1797-804.

438. Campmans-Kuijpers MJ, et al. The association of substituting carbohydrates with total fat and different types of fatty acids with mortality and weight change among diabetes patients. Clin Nutr. 2016;35(5):1096-102.

439. Campmans-Kuijpers MJE, et al. Isocaloric substitution of carbohydrates with protein: The association with weight change and mortality among patients with type 2 diabetes. Cardiovascular Diabetology. 2015;14(1).

440. Chen CH, et al. Association of Sugar-Sweetened Beverages and Cardiovascular Diseases Mortality in a Large Young Cohort of Nearly 300,000 Adults (Age 20-39). Nutrients. 2022;14(13).

441. Chen GC, et al. Microvascular Disease, Cardiovascular Health, and Risk of Coronary Heart Disease in Type 2 Diabetes: A UK Biobank Study. Journal of Clinical Endocrinology and Metabolism. 2024;109(9):2335-42.

442. Chen HC, et al. Associations of fish oil with cardiovascular disease events: results from the Taiwan longitudinal study in aging. BMC Public Health. 2024;24(1):1979.

443. Chen Q, et al. Associations between serum calcium, phosphorus and mortality among patients with coronary heart disease. Eur J Nutr. 2018;57(7):2457-67.

444. Chen X, et al. Vitamin D and heart failure risk among individuals with type 2 diabetes: observational and Mendelian randomization studies. Am J Clin Nutr. 2024;120(3):491-8.

445. Chen Y, et al. Fresh fruit consumption, physical activity, and five-year risk of mortality among patients with type 2 diabetes: A prospective follow-up study. Nutrition, Metabolism and Cardiovascular Diseases. 2022;32(4):878-88.

446. Chen Z, et al. Dietary Glutamine and Glutamate in Relation to Cardiovascular Disease Incidence and Mortality in the United States Men and Women with Diabetes Mellitus. Journal of Nutrition. 2023;153(11):3247-58.

447. Chien KL, et al. Total 25-hydroxyvitamin D concentration as a predictor for all-cause death and cardiovascular event risk among ethnic Chinese adults: a cohort study in a Taiwan community. Plos One. 2015;10(3):e0123097.

448. Choi Y, et al. Association of Cardiovascular Health Score With Early- and Later-Onset Diabetes and With Subsequent Vascular Complications of Diabetes. J Am Heart Assoc. 2023;12(1):e027558.

449. Chonchol M, et al. Serum phosphorus and cardiovascular mortality in type 2 diabetes. American journal of medicine. 2009;122(4):380-6.

450. Cruijsen E, et al. Dairy consumption and mortality after myocardial infarction: A prospective analysis in the Alpha Omega Cohort. American Journal of Clinical Nutrition. 2021;114(1):59-69.

451. Cruijsen E, et al. Vitamin D status, physical activity and long-term mortality risk after myocardial infarction: a prospective analysis in the Alpha Omega Cohort. Eur J Prev Cardiol. 2024.

452. de Fine Olivarius N, et al. Predictors of mortality of patients newly diagnosed with clinical type 2 diabetes: A 5-year follow up study. BMC Endocrine Disorders. 2010;10.

453. Del Gobbo LC, et al. ω-3 Polyunsaturated fatty acid biomarkers and coronary heart disease: Pooling project of 19 cohort studies. JAMA Internal Medicine. 2016;176(8):1155-66.

454. Dhar I, et al. Plasma methionine and risk of acute myocardial infarction: Effect modification by established risk factors. Atherosclerosis. 2018;272:175-81.

455. Díez-Espino J, et al. Egg consumption and cardiovascular disease according to diabetic status: The PREDIMED study. Clinical Nutrition. 2017;36(4):1015-21.

456. Dijkstra SC, et al. Intake of very long chain n-3 fatty acids from fish and the incidence of heart failure: the Rotterdam Study. Eur J Heart Fail. 2009;11(10):922-8.

457. Djoussé L, Gaziano JM. Egg consumption in relation to cardiovascular disease and mortality: the Physicians' Health Study. Am J Clin Nutr. 2008;87(4):964-9.

458. Djoussé L, et al. Egg consumption and risk of coronary artery disease in the Million Veteran Program. Clinical Nutrition. 2020;39(9):2842-7.

459. Djoussé L, et al. Egg consumption, overall diet quality, and risk of type 2 diabetes and coronary heart disease: A pooling project of US prospective cohorts. Clinical Nutrition. 2021;40(5):2475-82.

460. dos Santos ALT, et al. Dietary fat composition and cardiac events in patients with type 2 diabetes. Atherosclerosis. 2014;236(1):31e8.

461. Drouin-Chartier JP, et al. Egg consumption and risk of cardiovascular disease: Three large prospective US cohort studies, systematic review, and updated meta-analysis. The BMJ. 2020;368.

462. Du H, et al. Fresh fruit consumption in relation to incident diabetes and diabetic vascular complications: A 7-y prospective study of 0.5 million Chinese adults. PLoS Medicine. 2017;14(4).

463. Dziopa K, et al. Identifying and ranking novel independent features for cardiovascular disease prediction in people with type 2 diabetes. 2023.

464. Ekinci EI, et al. Dietary salt intake and mortality in patients with type 2 diabetes. Diabetes Care. 2011;34(3):703-9.

465. Espe KM, et al. Low plasma α-tocopherol concentrations and adverse clinical outcomes in diabetic hemodialysis patients. Clinical journal of the American Society of Nephrology. 2013;8(3):452-8.

466. Espe KM, et al. Impact of vitamin A on clinical outcomes in haemodialysis patients. Nephrol Dial Transplant. 2011;26(12):4054-61.

467. Estlin AFL, et al. Modification of cardiovascular disease risk by health behaviour change following type 2 diabetes diagnosis. Diabetic Medicine. 2021;38(10).

468. Evers I, et al. Dietary magnesium and risk of cardiovascular and all-cause mortality after myocardial infarction: A prospective analysis in the Alpha Omega Cohort. Frontiers in Cardiovascular Medicine. 2022;9.

469. Fang X, et al. Association between fresh fruit intake and cardiovascular disease risk in patients with diabetes: A prospective cohort study. Chin Med J (Engl). 2024.

470. Gao Y, et al. Lifestyle trajectories and ischaemic heart diseases: a prospective cohort study in UK Biobank. European Journal of Preventive Cardiology. 2023;30(5):393-403.

471. Gutiérrez-Bedmar M, et al. Chromium exposure and risk of cardiovascular disease in high cardiovascular risk subjects: Nested case-control study in the prevention with mediterranean diet (PREDIMED) study. Circulation Journal. 2017;81(8):1183-90.

472. Han H, et al. Association of a Healthy Lifestyle With All-Cause and Cause-Specific Mortality Among Individuals With Type 2 Diabetes: A Prospective Study in UK Biobank. Diabetes Care. 2022;45(2):319-29.

473. Han T, et al. The Association of Energy and Macronutrient Intake at Dinner Versus Breakfast With Disease-Specific and All-Cause Mortality Among People With Diabetes: The U.S. National Health and Nutrition Examination Survey, 2003-2014. Diabetes Care. 2020;43(7):1442-8.

474. Harris K, et al. Plasma fatty acids and the risk of vascular disease and mortality outcomes in individuals with type 2 diabetes: results from the ADVANCE study. Diabetologia. 2020;63(8):1637-47.

475. He M, et al. Whole-grain, cereal fiber, bran, and germ intake and the risks of all-cause and cardiovascular disease-specific mortality among women with type 2 diabetes mellitus. Circulation. 2010;121(20):2162-8.

476. Heianza Y, et al. Plasma Levels of Polyols Erythritol, Mannitol, and Sorbitol and Incident Coronary Heart Disease Among Women. European journal of preventive cardiology. 2024.

477. Heidari B, et al. Assessment of serum 25-hydroxy vitamin D improves coronary heart disease risk stratification in patients with type 2 diabetes. Am Heart J. 2015;170(3):573-9.e5.

478. Ho YL, et al. Chocolate consumption and risk of coronary artery disease: The Million Veteran Program. American Journal of Clinical Nutrition. 2021;113(5):1137-44.

479. Horikawa C, et al. Sodium intake and incidence of diabetes complications in elderly patients with type 2 diabetes—analysis of data from the japanese elderly diabetes intervention study (J-edit). Nutrients. 2021;13(2):1-13.

480. Horikawa C, et al. Higher calcium intake is associated with lower incidence of diabetic nephropathy in Japanese patients with type 2 diabetes. Diabetes. 2019;68.

481. Horikawa C, et al. Meat intake and incidence of cardiovascular disease in Japanese patients with type 2 diabetes: analysis of the Japan Diabetes Complications Study (JDCS). European Journal of Nutrition. 2019;58(1):281-90.

482. Horikawa C, et al. Dietary potassium intake and its interaction with sodium intake on risk of developing cardiovascular disease in persons with type 2 diabetes: The Japan Diabetes Complication and its Prevention Prospective study (JDCP study 12). Diabetes, Obesity and Metabolism. 2025;27(1):394-406.

483. Horikawa C, et al. Dietary sodium intake and incidence of diabetes complications in Japanese patients with type 2 diabetes: Analysis of the Japan diabetes complications study (JDCS). Journal of Clinical Endocrinology and Metabolism. 2014;99(10):3635-43.

484. Horikawa C, et al. Is the proportion of carbohydrate intake associated with the incidence of diabetes complications?—an analysis of the Japan diabetes complications study. Nutrients. 2017;9(2).

485. Houston DK, et al. Dietary fat and cholesterol and risk of cardiovascular disease in older adults: The Health ABC Study. Nutrition, Metabolism and Cardiovascular Diseases. 2011;21(6):430-7.

486. Hu FB, et al. Fish and long-chain omega-3 fatty acid intake and risk of coronary heart disease and total mortality in diabetic women. Circulation. 2003;107(14):1852-7.

487. Hu J, et al. Weight Change, Lifestyle, and Mortality in Patients with Type 2 Diabetes. Journal of Clinical Endocrinology and Metabolism. 2022;107(3):627-37.

488. Hu L, et al. Association between plasma copper levels and first stroke: a community-based nested case–control study. Nutritional Neuroscience. 2022;25(7):1524-33.

489. Hua R, et al. Association between dietary supplement use and mortality among US adults with diabetes: a longitudinal cohort study. Nutr Metab (Lond). 2023;20(1):33.

490. Hua R, et al. Regular Use of Fish Oil Supplements, Life's Essential 8 Score, and Cardiovascular Mortality in People With Type 2 Diabetes: A Longitudinal Cohort Study. Journal of Nutrition. 2024.

491. Huang N, et al. Associations of eicosapentaenoic acid and docosahexaenoic acid intakes with cardiovascular and all-cause mortality in patients with diabetes: Result from National Health and Nutrition Examination Survey 1999-2008. Front Cardiovasc Med. 2022;9:1031168.

492. Huang R, et al. Associations of dietary magnesium intake with the risk of atherosclerotic cardiovascular disease and mortality in individuals with and without type 2 diabetes: A prospective study in the UK Biobank. Diabetes Metab. 2024;50(5):101554.

493. Huang ZG, et al. Comprehensive Multiple Risk Factor Control in Type 2 Diabetes to Mitigate Heart Failure Risk: Insights From a Prospective Cohort Study. Diabetes Care. 2024;47(10):1818-25.

494. Ikehara S, et al. Peanut Consumption and Risk of Stroke and Ischemic Heart Disease in Japanese Men and Women: The JPHC Study. Stroke. 2021;52(11):3543-50.

495. Inan-Eroglu E, et al. Association between Protein Intake and Diabetes Complications Risk Following Incident Type 2 Diabetes: The EPIC-Potsdam Study. Metabolites. 2024;14(3).

496. Iwase M, et al. Incidence of stroke and its association with glycemic control and lifestyle in Japanese patients with type 2 diabetes mellitus: The Fukuoka diabetes registry. Diabetes Research and Clinical Practice. 2021;172.

497. Jang J, et al. Longitudinal association between egg consumption and the risk of cardiovascular disease: Interaction with type 2 diabetes mellitus. Nutrition and Diabetes. 2018;8(1).

498. Jiang W, et al. The Association of Consumption Time for Food With Cardiovascular Disease and All-Cause Mortality Among Diabetic Patients. J Clin Endocrinol Metab. 2022;107(7):e3066-e75.

499. Jiao J, et al. Dietary fats and mortality among patients with type 2 diabetes: Analysis in two population based cohort studies. The BMJ. 2019;366.

500. Jin Q, et al. Circulating metabolomic markers linking diabetic kidney disease and incident cardiovascular disease in type 2 diabetes: analyses from the Hong Kong Diabetes Biobank. Diabetologia. 2024;67(5):837-49.

501. Joergensen C, et al. Vitamin D levels and mortality in type 2 diabetes. Diabetes Care. 2010;33(10):2238-43.

502. Joshipura KJ, et al. The effect of fruit and vegetable intake on risk for coronary heart disease. Annals of Internal Medicine. 2001;134(12):1106-14+I-28.

503. Klipstein-Grobusch K, et al. Serum ferritin and risk of myocardial infarction in the elderly: The Rotterdam Study. American Journal of Clinical Nutrition. 1999;69(6):1231-6.

504. Knekt P, et al. Quercetin intake and the incidence of cerebrovascular disease. European Journal of Clinical Nutrition. 2000;54(5):415-7.

505. Komorita Y, et al. Additive effects of green tea and coffee on all-cause mortality in patients with type 2 diabetes mellitus: the Fukuoka Diabetes Registry. BMJ Open Diabetes Res Care. 2020;8(1).

506. Kuntsevich AK, et al. ASSOCIATION OF DIETARY CHOLESTEROL INTAKE WITH THE RISK OF FATAL CARDIOVASCULAR EVENTS IN INDIVIDUALS WITH TYPE 2 DIABETES. Diabetes Mellitus. 2023;26(4):318-27.

507. Kunutsor SK, et al. Serum copper-to-zinc ratio is associated with heart failure and improves risk prediction in middle-aged and older Caucasian men: A prospective study. Nutrition, Metabolism and Cardiovascular Diseases. 2022;32(8):1924-35.

508. Kwon YJ, et al. Association of Dietary Fiber Intake with All-Cause Mortality and Cardiovascular Disease Mortality: A 10-Year Prospective Cohort Study. Nutrients. 2022;14(15).

509. Kyte B, et al. High red blood cell folate is associated with an increased risk of death among adults with diabetes, a 15-year follow-up of a national cohort. Nutr Metab Cardiovasc Dis. 2015;25(11):997-1006.

510. Laguna JC, et al. Simple sugar intake and cancer incidence, cancer mortality and all-cause mortality: a cohort study from the PREDIMED trial. Clinical nutrition (Edinburgh, Scotland). 2021;40(10):5269-77.

511. Larsson SC, et al. Egg consumption and risk of heart failure, myocardial infarction, and stroke: Results from 2 prospective cohorts. American Journal of Clinical Nutrition. 2015;102(5):1007-13.

512. Larsson SC, et al. Coffee consumption and risk of stroke in women. Stroke. 2011;42(4):908-12.

513. Lee DH, et al. Does supplemental vitamin C increase cardiovascular disease risk in women with diabetes? Am J Clin Nutr. 2004;80(5):1194-200.

514. Lemaitre RN, et al. Plasma epoxyeicosatrienoic acids and diabetes-related cardiovascular disease: The cardiovascular health study. eBioMedicine. 2022;83.

515. Lemaitre RN, et al. Circulating very long-chain saturated fatty acids and heart failure: The cardiovascular health study. Journal of the American Heart Association. 2018;7(21).

516. Lemaitre RN, et al. Circulating and dietary α-linolenic acid and incidence of congestive heart failure in older adults: The Cardiovascular Health Study. American Journal of Clinical Nutrition. 2012;96(2):269-74.

517. Li D, et al. Adherence to a Healthy Lifestyle and the Risk of All-Cause Mortality and Cardiovascular Events in Individuals With Diabetes: The ARIC Study. Front Nutr. 2021;8:698608.

518. Li F, et al. Vitamin B6 Turnover Predicts Long-term Mortality Risk in Patients with Type 2 Diabetes. Curr Dev Nutr. 2024;8(2):102073.

519. Li J, et al. Impact of live microbe intake on cardiovascular disease and mortality in adults with diabetes: A nationwide cohort study. Diabetes Research and Clinical Practice. 2025;219.

520. Li Q, et al. Potential benefits of spicy food consumption on cardiovascular outcomes in patients with diabetes: A cohort study of the China Kadoorie Biobank. Nutrition. 2023;112.

521. Li Q, et al. Associations of serum magnesium levels and calcium-magnesium ratios with mortality in patients with coronary artery disease. Diabetes & metabolism. 2020;46(5):384-91.

522. Li TY, et al. Regular consumption of nuts is associated with a lower risk of cardiovascular disease in women with type 2 diabetes. Journal of Nutrition. 2009;139(7):1333-8.

523. Li X, et al. Tea consumption and risk of ischaemic heart disease. Heart. 2017;103(10):783-9.

524. Lilja E, et al. The association between dietary intake, lifestyle and incident symptomatic peripheral arterial disease among individuals with diabetes mellitus: insights from the Malmö Diet and Cancer study. Ther Adv Endocrinol Metab. 2019;10:2042018819890532.

525. Lim LL, et al. Circulating branched-chain amino acids and incident heart failure in type 2 diabetes: The Hong Kong Diabetes Register. Diabetes/Metabolism Research and Reviews. 2020;36(3).

526. Lin CC, et al. Impact of lifestyle-related factors on all-cause and cause-specific mortality in patients with type 2 diabetes: the Taichung Diabetes Study. Diabetes Care. 2012;35(1):105-12.

527. Lin CC, et al. Dietary macronutrient intakes and mortality among patients with type 2 diabetes. Nutrients. 2020;12(6):1-13.

528. Lin L, et al. Association of dietary niacin intake with all-cause and cardiovascular mortality: National Health and Nutrition Examination Survey (NHANES) 2003-2018. Sci Rep. 2024;14(1):28313.

529. Liu B, et al. Green tea consumption and incidence of cardiovascular disease in type 2 diabetic patients with overweight/obesity: a community-based cohort study. Arch Public Health. 2024;82(1):18.

530. Liu D, et al. Trimethylamine N-oxide, β-alanine, tryptophan index, and vitamin B6-related dietary patterns in association with stroke risk. Nutrition, Metabolism and Cardiovascular Diseases. 2024;34(5):1179-88.

531. Liu G, et al. Nut Consumption in Relation to Cardiovascular Disease Incidence and Mortality among Patients with Diabetes Mellitus. Circulation Research. 2019;124(6):920-9.

532. Liu JJ, et al. Estimated potassium intake and major adverse cardiovascular events in individuals with type 2 diabetes: a prospective cohort study with trans-ethnic validation. Cardiovascular Diabetology. 2024;23(1).

533. Liu M, et al. Relationship of ultra-processed food consumption and new-onset chronic kidney diseases among participants with or without diabetes. Diabetes and Metabolism. 2023;49(4).

534. Liu S, et al. Is intake of breakfast cereals related to total and cause-specific mortality in men? American journal of clinical nutrition. 2003;77(3):594-9.

535. Liu W, et al. Daily folate consumption is associated with reduced all-cause and cardiovascular disease mortality among US adults with diabetes, prediabetes, or insulin resistance. Nutrition Research. 2023;114:71-80.

536. Liu W, et al. Association between dietary vitamin intake and mortality in US adults with diabetes: A prospective cohort study. Diabetes Metab Res Rev. 2024;40(2):e3729.

537. Liu X, et al. Association of Fish Oil Supplementation with Risk of Coronary Heart Disease in Individuals with Diabetes and Prediabetes: A Prospective Study in the UK Biobank. Nutrients. 2023;15(14).

538. Liu Y, et al. Associations of Serum Folate and Vitamin B12 Levels With Cardiovascular Disease Mortality Among Patients With Type 2 Diabetes. JAMA Netw Open. 2022;5(1):e2146124.

539. Liu Y, et al. Association of Serum 25(OH)D, Cadmium, CRP With All-Cause, Cause-Specific Mortality: A Prospective Cohort Study. Front Nutr. 2022;9:803985.

540. Liu YJ, et al. Coffee Consumption and Incidence of Cardiovascular and Microvascular Diseases in Never-Smoking Adults with Type 2 Diabetes Mellitus. Nutrients. 2023;15(18).

541. Long GH, et al. Healthy behavior change and cardiovascular outcomes in newly diagnosed type 2 diabetic patients: a cohort analysis of the ADDITION-Cambridge study. Diabetes care. 2014;37(6):1712-20.

542. Long T, et al. Plasma metals and cardiovascular disease in patients with type 2 diabetes. Environment International. 2019;129:497-506.

543. Looker HC, et al. Homocysteine and vitamin B(12) concentrations and mortality rates in type 2 diabetes. Diabetes Metab Res Rev. 2007;23(3):193-201.

544. Lopez-Garcia E, et al. Coffee consumption and risk of stroke in women. Circulation. 2009;119(8):1116-23.

545. Lopez-Garcia E, et al. Coffee consumption and coronary heart disease in men and women: A prospective cohort study. Circulation. 2006;113(17):2045-53.

546. Lu J, et al. Association of serum iron with all-cause mortality and cardiovascular mortality in the cardiovascular patients: a retrospective cohort study based on the NHANES 1999–2018. Frontiers in Cardiovascular Medicine. 2024;11.

547. Lu X, et al. Habitual Coffee, Tea, and Caffeine Consumption, Circulating Metabolites, and the Risk of Cardiometabolic Multimorbidity. J Clin Endocrinol Metab. 2024.

548. Ma L, et al. Beverage consumption and mortality among adults with type 2 diabetes: prospective cohort study. BMJ. 2023.

549. Marklund M, et al. Biomarkers of Dietary Omega-6 Fatty Acids and Incident Cardiovascular Disease and Mortality: An Individual-Level Pooled Analysis of 30 Cohort Studies. Circulation. 2019;139(21):2422-36.

550. Mozaffarian D, et al. Cereal, Fruit, and Vegetable Fiber Intake and the Risk of Cardiovascular Disease in Elderly Individuals. JAMA. 2003;289(13):1659-66.

551. Nie J, et al. Tea consumption and long-term risk of type 2 diabetes and diabetic complications: A cohort study of 0.5 million Chinese adults. American Journal of Clinical Nutrition. 2021;114(1):194-202.

552. Nöthlings U, et al. Intake of vegetables, legumes, and fruit, and risk for all-cause, cardiovascular, and cancer mortality in a European diabetic population. Journal of Nutrition. 2008;138(4):775-81.

553. Ottosson F, et al. Plasma metabolites associate with all-cause mortality in individuals with type 2 diabetes. Metabolites. 2020;10(8):1-11.

554. Ou Y, et al. Associations of serum vitamin C concentrations with risk of all-cause and cause-specific mortality among individuals with and without type 2 diabetes. Eur J Nutr. 2023;62(6):2555-65.

555. Pan Y, et al. Decreased free fatty acid levels associated with adverse clinical outcomes in coronary artery disease patients with type 2 diabetes: findings from the PRACTICE study. Eur J Prev Cardiol. 2023;30(8):730-9.

556. Papandreou C, et al. Legume consumption and risk of all-cause, cardiovascular, and cancer mortality in the PREDIMED study. Clin Nutr. 2019;38(1):348-56.

557. Qi L, et al. Heme iron from diet as a risk factor for coronary heart disease in women with type 2 diabetes. Diabetes Care. 2007;30(1):101-6.

558. Qin C, et al. Associations of egg consumption with cardiovascular disease in a cohort study of 0.5 million Chinese adults. Heart. 2018;104(21):1756-63.

559. Qiu Z, et al. Associations of serum carotenoids with risk of cardiovascular mortality among individuals with type 2 diabetes: Results from nhanes. Diabetes Care. 2022;45(6):1453-61.

560. Qiu Z, et al. Associations of Habitual Calcium Supplementation With Risk of Cardiovascular Disease and Mortality in Individuals With and Without Diabetes. Diabetes Care. 2024;47(2):199-207.

561. Qureshi AI, et al. Regular egg consumption does not increase the risk of stroke and cardiovascular diseases. Medical Science Monitor. 2007;13(1):CR1-CR8.

562. Raffield LM, et al. Cross-sectional analysis of calcium intake for associations with vascular calcification and mortality in individuals with type 2 diabetes from the Diabetes Heart Study. American Journal of Clinical Nutrition. 2014;100(4):1029-35.

563. Samefors M, et al. Association between serum 25(OH)D(3) and cardiovascular morbidity and mortality in people with Type 2 diabetes: a community-based cohort study. Diabet Med. 2017;34(3):372-9.

564. Saulnier PJ, et al. Urinary Sodium Concentration Is an Independent Predictor of All-Cause and Cardiovascular Mortality in a Type 2 Diabetes Cohort Population. Journal of Diabetes Research. 2017;2017.

565. Schrieks IC, et al. Adiponectin, Free Fatty Acids, and Cardiovascular Outcomes in Patients With Type 2 Diabetes and Acute Coronary Syndrome. Diabetes care. 2018;41(8):1792-800.

566. Scrafford CG, et al. Egg consumption and CHD and stroke mortality: a prospective study of US adults. Public health nutrition. 2011;14(2):261-70.

567. Silletta MG, et al. Coffee consumption and risk of cardiovascular events after acute myocardial infarction: results from the GISSI (Gruppo Italiano per lo Studio della Sopravvivenza nell'Infarto miocardico)-Prevenzione trial. Circulation. 2007;116(25):2944-51.

568. Soinio M, et al. Dietary fat predicts coronary heart disease events in subjects with type 2 diabetes. Diabetes Care. 2003;26(3):619-24.

569. Sotos-Prieto M, et al. Application of a Lifestyle-Based Score to Predict Cardiovascular Risk in African Americans: The Jackson Heart Study. J Clin Med. 2021;10(11).

570. Strand E, et al. Dietary intake of n-3 long-chain polyunsaturated fatty acids and risk of myocardial infarction in coronary artery disease patients with or without diabetes mellitus: a prospective cohort study. BMC medicine. 2013;11:216.

571. Su H, et al. Associations of folate intake with all-cause and cause-specific mortality among individuals with diabetes. Front Nutr. 2022;9:1021709.

572. Su S, et al. Vitamin D deficiency in diabetes exacerbates longitudinal risk for atherosclerotic cardiovascular disease in Lanzhou, China. Asia Pac J Clin Nutr. 2021;30(4):557-65.

573. Sun D, et al. Association of coffee consumption with cardiometabolic multimorbidity: A prospective cohort study in the UK biobank. Nutr Metab Cardiovasc Dis. 2024;34(12):2779-88.

574. Sun Z, et al. Low-risk Lifestyle and Health Factors and Risk of Mortality and Vascular Complications in Chinese Patients With Diabetes. Journal of Clinical Endocrinology and Metabolism. 2022;107(9):E3919-E28.

575. Tanaka S, et al. Intakes of dietary fiber, vegetables, and fruits and incidence of cardiovascular disease in Japanese patients with type 2 diabetes. Diabetes care. 2013;36(12):3916-22.

576. Tanasescu M, et al. Dietary fat and cholesterol and the risk of cardiovascular disease among women with type 2 diabetes. Am J Clin Nutr. 2004;79(6):999-1005.

577. Tang S, et al. Individual cereals intake is associated with progression of diabetes and diabetic chronic complications. Diabetes and Metabolic Syndrome: Clinical Research and Reviews. 2024;18(9).

578. Tian C, et al. Green tea consumption is associated with reduced incident CHD and improved CHD-related biomarkers in the Dongfeng-Tongji cohort. Scientific reports. 2016;6:24353.

579. Tian S, et al. Fish Oil, Plasma n-3 PUFAs, and Risk of Macro- and Microvascular Complications among Individuals with Type 2 Diabetes. The Journal of clinical endocrinology and metabolism. 2024.

580. Tobias DK, et al. Circulating branched-chain amino acids and incident cardiovascular disease in a prospective cohort of us women. Circulation. 2018;137.

581. Tougaard NH, et al. Vitamin D deficiency and development of complications in individuals with type 1 and type 2 diabetes: A cohort study. J Diabetes Complications. 2023;37(10):108611.

582. Veronese N, et al. Serum 25-Hydroxyvitamin D and the Incidence of Peripheral Artery Disease in the Elderly: The Pro.V.A Study. J Atheroscler Thromb. 2015;22(7):726-34.

583. Vissers LET, et al. The relationship between vitamin K and peripheral arterial disease. Atherosclerosis. 2016;252:15-20.

584. Wallin A, et al. Fish consumption in relation to myocardial infarction, stroke and mortality among women and men with type 2 diabetes: A prospective cohort study. Clinical Nutrition. 2018;37(2):590-6.

585. Wan Z, et al. Vitamin D status, genetic factors, and risks of cardiovascular disease among individuals with type 2 diabetes: a prospective study. Am J Clin Nutr. 2022;116(5):1389-99.

586. Wan Z, et al. Association of Serum 25-Hydroxyvitamin D Concentrations With All-Cause and Cause-Specific Mortality Among Individuals With Diabetes. Diabetes Care. 2021;44(2):350-7.

587. Wang DD, et al. Dietary Sodium and Potassium Intake and Risk of Non-Fatal Cardiovascular Diseases: The Million Veteran Program. Nutrients. 2022;14(5).

588. Wang H, et al. Association between oxidative balance scores and all-cause and cardiovascular disease-related mortality in patients with type 2 diabetes: data from the national health and nutrition examination survey (2007-2018). BMC Public Health. 2024;24(1):2642.

589. Wang HW, et al. Association of dietary magnesium intake and glycohemoglobin with mortality risk in diabetic patients. PLoS One. 2022;17(12):e0277180.

590. Wang Q, et al. Association of Dietary Flavonoids Intake With All-Cause and Cardiovascular Disease Mortality in Diabetic Kidney Disease: A Cohort Study From the NHANES Database. J Diabetes Res. 2024;2024:8359294.

591. Wang R, et al. Association of composite dietary antioxidant index with cardiovascular disease in adults: results from 2011 to 2020 NHANES. Front Cardiovasc Med. 2024;11:1379871.

592. Wang S, et al. Cobalamin Intake and Related Biomarkers: Examining Associations With Mortality Risk Among Adults With Type 2 Diabetes in NHANES. Diabetes Care. 2022;45(2):276-84.

593. Wang X, et al. Dietary soy consumption and cardiovascular mortality among Chinese people with type 2 diabetes. Nutrients. 2021;13(8).

594. Wang Y, Chen H. Usage of table salt and risk of all-cause and cardiovascular disease mortality among patients with diabetes: a national population-based cohort study. Diabetology and Metabolic Syndrome. 2024;16(1).

595. Wang Y, et al. Coffee consumption and the risk of heart failure in Finnish men and women. Heart. 2011;97(1):44-8.

596. Wargny M, et al. Nutritional biomarkers and heart failure requiring hospitalization in patients with type 2 diabetes: the SURDIAGENE cohort. Cardiovascular Diabetology. 2022;21(1).

597. Wei Y, et al. Joint Associations between Plasma 25-Hydroxyvitamin D, Glycemic Status, and First Stroke in General Hypertensive Adults: results from the China Stroke Primary Prevention Trial (CSPPT). Journal of nutrition. 2022;152(1):246-54.

598. Wikström K, et al. Clinical and lifestyle-related risk factors for incident multimorbidity: 10-year follow-up of Finnish population-based cohorts 1982-2012. European Journal of Internal Medicine. 2015;26(3):211-6.

599. Wu PY, et al. Alternative health eating index and the Dietary Guidelines from American Diabetes Association both may reduce the risk of cardiovascular disease in type 2 diabetes patients. J Hum Nutr Diet. 2016;29(3):363-73.

600. Wu Y, et al. Association between dietary protein intake and mortality among patients with diabetic kidney disease. Diabetes Metab Syndr. 2024;18(7):103091.

601. Xie J, et al. Intakes of omega-3 fatty acids and risks of all-cause and cause-specific mortality in people with diabetes: a cohort study based on NHANES 1999-2014. Acta Diabetol. 2023;60(3):353-62.

602. Xiong H, et al. Folate Status and Mortality in US Adults With Diabetes: A Nationally Representative Cohort Study. Frontiers in Cardiovascular Medicine. 2022;9.

603. Xu D, et al. Higher dietary intake of aromatic amino acids was associated with lower risk of cardiovascular disease mortality in adult participants in NHANES III. Nutrition Research. 2023;113:39-48.

604. Xu X, et al. Dietary fibre and mortality risk in patients on peritoneal dialysis. Br J Nutr. 2019;122(9):996-1005.

605. Xu Z, et al. Association between the oxidative balance score and all-cause and cardiovascular mortality in patients with diabetes and prediabetes. Redox Biol. 2024;76:103327.

606. Yakti FAH, et al. Higher egg consumption and incident cardiovascular disease in Chinese adults - 10-Year follow-up results from China health and nutrition survey. Nutrition, Metabolism and Cardiovascular Diseases. 2024;34(11):2537-45.

607. Yang C, et al. Dietary iron intake predicts all-cause and cardiovascular mortality in patients with diabetes. Nutrition and Diabetes. 2024;14(1).

608. Yang F, et al. Predicting life span of type 2 diabetes patients through alkaline phosphatase and vitamin D: Results from NHANES 1999-2018. Atherosclerosis. 2024;394:117318.

609. Yang R, et al. Relationship between timing of coffee and tea consumption with mortality (total, cardiovascular disease and diabetes) in people with diabetes: the U.S. National Health and Nutrition Examination Survey, 2003-2014. BMC Med. 2024;22(1):526.

610. Yang R, et al. A healthy lifestyle mitigates the risk of heart disease related to type 2 diabetes: a prospective nested case-control study in a nationwide Swedish twin cohort. Diabetologia. 2021;64(3):530-9.

611. Yang R, et al. Niacin intake and mortality (total and cardiovascular disease) in patients with cardiovascular disease: Insights from NHANES 2003–2018. Nutrition Journal. 2024;23(1).

612. Yao H, et al. Association of caffeine intake with all-cause and cardiovascular mortality in diabetes and prediabetes. Diabetol Metab Syndr. 2024;16(1):177.

613. Ye H, et al. Association between serum 25-hydroxyvitamin D and vitamin D dietary supplementation and risk of all-cause and cardiovascular mortality among adults with hypertension. Nutr J. 2024;23(1):33.

614. Ying AF, et al. Consumption of different types of meat and the risk of chronic limb-threatening ischemia: the Singapore Chinese Health Study. Nutrition Journal. 2024;23(1).

615. Yun JS, et al. Associations between polygenic risk of coronary artery disease and type 2 diabetes, lifestyle, and cardiovascular mortality: A prospective UK Biobank study. Frontiers in Cardiovascular Medicine. 2022;9.

616. Zhang A, et al. Associations of serum lead, cadmium, and mercury concentrations with all-cause and cause-specific mortality among individuals with cardiometabolic multimorbidity. Ecotoxicology and Environmental Safety. 2024;280.

617. Zhang J, et al. Prognostic nutritional index as a risk factor for diabetic kidney disease and mortality in patients with type 2 diabetes mellitus. Acta Diabetologica. 2023;60(2):235-45.

618. Zhang P, et al. Association of Serum 25-Hydroxyvitamin D With Cardiovascular Outcomes and All-Cause Mortality in Individuals With Prediabetes and Diabetes: Results From the UK Biobank Prospective Cohort Study. Diabetes Care. 2022;45(5):1219-29.

619. Zhang W, et al. Luteolin intake is negatively associated with all-cause and cardiac mortality among patients with type 2 diabetes mellitus. Diabetology and Metabolic Syndrome. 2023;15(1).

620. Zhang W, et al. Coffee consumption and risk of cardiovascular diseases and all-cause mortality among men with type 2 diabetes. Diabetes Care. 2009;32(6):1043-5.

621. Zhang WL, et al. Coffee consumption and risk of cardiovascular events and all-cause mortality among women with type 2 diabetes. Diabetologia. 2009;52(5):810-7.

622. Zhang Y, et al. Lifestyle factors on the risks of ischemic and hemorrhagic stroke. Archives of Internal Medicine. 2011;171(20):1811-8.

623. Zhang Y, et al. Association of cardiovascular health with diabetic complications, all-cause mortality, and life expectancy among people with type 2 diabetes. Diabetol Metab Syndr. 2022;14(1):158.

624. Zhang Y, et al. Associations of different isomeric forms of serum lycopene with cardiovascular disease and all-cause mortality. Int J Vitam Nutr Res. 2024;94(2):108-19.

625. Zheng G, et al. Effect modification of dietary diversity on the association of air pollution with incidence, complications, and mortality of type 2 diabetes: Results from a large prospective cohort study. Science of the Total Environment. 2024;908.

626. Zheng Y, et al. Dietary phosphatidylcholine and risk of all-cause and cardiovascular-specific mortality among US women and men. American Journal of Clinical Nutrition. 2016;104(1):173-80.

627. Zhu K, et al. Beverage Consumption, Genetic Predisposition, and Risk of Cardiovascular Disease Among Adults With Type 2 Diabetes. J Clin Endocrinol Metab. 2024;109(11):e2038-e47.

628. Zhuang J. Effect of dietary iron intake on all-cause and cardiovascular mortality in patients with diabetes mellitus. Zhongguo Dongmai Yinghua Zazhi. 2024;32(9):777-82.

629. Zuo H, et al. The PAr index, an indicator reflecting altered vitamin B-6 homeostasis, is associated with long-term risk of stroke in the general population: the Hordaland Health Study (HUSK). Am J Clin Nutr. 2018;107(1):105-12.

630. Hu FB, et al. A prospective study of egg consumption and risk of cardiovascular disease in men and women. JAMA. 1999;281(15):1387-94.

631. Lacson E, et al. Serum magnesium and mortality in hemodialysis patients in the United States: A cohort study. American Journal of Kidney Diseases. 2015;66(6):1056-66.

632. Morris MC, et al. Fish consumption and cardiovascular disease in the physicians' health study: a prospective study. American journal of epidemiology. 1995;142(2):166-75.

633. Asmar J, et al. Anemia biomarkers and mortality in hemodialysis patients with or without diabetes: A 10-year follow-up study. PLoS ONE. 2023;18(1 January).

634. Chen L, et al. Association between dietary magnesium intake and all-cause mortality among patients with diabetic retinopathy: a retrospective cohort study of the NHANES 1999-2018. Magnes Res. 2024;37(1):22-32.

635. Cordova R, et al. Consumption of ultra-processed foods and risk of multimorbidity of cancer and cardiometabolic diseases: a multinational cohort study. The Lancet Regional Health - Europe. 2023;35.

636. Ekinci EI, et al. Relationship between urinary sodium excretion over time and mortality in type 2 diabetes. Diabetes Care. 2014;37(4):e62-e3.

637. Freedman ND, et al. Association of coffee drinking with total and cause-specific mortality. N Engl J Med. 2012;366(20):1891-904.

638. Garcia-Arellano A, et al. Dietary inflammatory index and all-cause mortality in large cohorts: The SUN and PREDIMED studies. Clinical Nutrition. 2019;38(3):1221-31.

639. Horikawa C, et al. Dietary intake in Japanese patients with type 2 diabetes: Analysis from Japan Diabetes Complications Study. Journal of Diabetes Investigation. 2014;5(2):176-87.

640. Hshieh TT, et al. Nut consumption and risk of mortality in the physicians' health study. American Journal of Clinical Nutrition. 2015;101(2):407-12.

641. Huang J, et al. Association Between Plant and Animal Protein Intake and Overall and Cause-Specific Mortality. JAMA Intern Med. 2020;180(9):1173-84.

642. Iimuro S, et al. Dietary pattern and mortality in Japanese elderly patients with type2 diabetes mellitus: Does a vegetable- and fish-rich diet improve mortality? An explanatory study. Geriatrics and Gerontology International. 2012;12(SUPPL.1):59-67.

643. Jayedi A, et al. Intake of animal and plant proteins and risk of all-cause mortality in patients with type 2 diabetes: results from NHANES. European Journal of Clinical Nutrition. 2024.

644. Jayedi A, et al. Patterns of protein intake and mortality in patients with type 2 diabetes: results from NHANES. Sci Rep. 2024;14(1):26644.

645. Jenq CC, et al. Serum ferritin levels predict all-cause and infection-cause 1-year mortality in diabetic patients on maintenance hemodialysis. Am J Med Sci. 2009;337(3):188-94.

646. Ke C, et al. Association between dietary inflammatory index and all-cause mortality in patients with osteopenia or osteoporosis: A retrospective cohort study from the NHANES 2007-2018. Prev Med Rep. 2024;45:102826.

647. Krzyzanowska K, et al. Asymmetric dimethylarginine predicts cardiovascular events in patients with type 2 diabetes. Diabetes Care. 2007;30(7):1834-9.

648. Li Z, et al. Associations of healthy lifestyle and family income to poverty ratio with all-cause mortality among people with prediabetes and diabetes: a prospective cohort study. BMC Public Health. 2025;25(1):24.

649. Lindberg M, et al. Plasma phospholipid EPA and DHA are divergently associated with overall mortality in newly diagnosed diabetic patients: results from a follow-up of the Nord-Trondelag Health (HUNT) Study, Norway. J Nutr Sci. 2013;2:e35.

650. Liu Y, et al. Associations Between Dietary Intake of Tomato and Lycopene with All-Cause and Cancer-Specific Mortality in US Adults with Diabetes: Results From a Cohort Study. Nutr Cancer. 2024;76(10):974-84.

651. Loftfield E, et al. Association of Coffee Consumption With Overall and Cause-Specific Mortality in a Large US Prospective Cohort Study. Am J Epidemiol. 2015;182(12):1010-22.

652. Nöthlings U, et al. Lifestyle factors and mortality among adults with diabetes: findings from the European Prospective Investigation into Cancer and Nutrition-Potsdam study*. J Diabetes. 2010;2(2):112-7.

653. Omura T, et al. Assessing the association between optimal energy intake and all-cause mortality in older patients with diabetes mellitus using the Japanese Elderly Diabetes Intervention Trial. Geriatr Gerontol Int. 2020;20(1):59-65.

654. Park YH, et al. Difference in association of carbohydrate intake with all-cause mortality between middle-aged and older Korean adults with and without diabetes mellitus: A prospective study. Clinical Nutrition. 2024;43(5):1117-24.

655. Patel YR, et al. Adherence to healthy lifestyle factors and risk of death in men with diabetes mellitus: The Physicians' Health Study. Clin Nutr. 2018;37(1):139-43.

656. Srinonprasert V, et al. Vitamin D insufficiency predicts mortality among older men, but not women: A nationwide retrospective cohort from Thailand. Geriatr Gerontol Int. 2018;18(12):1585-90.

657. Stančáková Yaluri A, et al. Decreased 25-Hydroxy Vitamin D Level Is Associated with All-Cause Mortality in Patients with Type 2 Diabetes at High Cardiovascular Risk. Metabolites. 2023;13(8).

658. Tan J, et al. A Proinflammatory Diet May Increase Mortality Risk in Patients with Diabetes Mellitus. Nutrients. 2022;14(10).

659. Tang Y, et al. Association between dietary vitamin A intake and risk of cardiometabolic multimorbidity. Sci Rep. 2024;14(1):16656.

660. Trichopoulou A, et al. Diet and physical activity in relation to overall mortality amongst adult diabetics in a general population cohort. Journal of Internal Medicine. 2006;259(6):583-91.

661. Villegas R, et al. Fish, omega-3 long-chain fatty acids, and all-cause mortality in a low-income US population: Results from the Southern Community Cohort Study. Nutrition, Metabolism and Cardiovascular Diseases. 2015;25(7):651-8.

662. Wan Z, et al. Associations of Moderate Low-Carbohydrate Diets With Mortality Among Patients With Type 2 Diabetes: A Prospective Cohort Study. J Clin Endocrinol Metab. 2022;107(7):e2702-e9.

663. Wang J, et al. Association Between Dietary Antioxidant Quality Score (DAQS) and All-Cause Mortality in Hypertensive Adults: A Retrospective Cohort Study from the NHANES Database. Biol Trace Elem Res. 2024;202(11):4978-87.

664. Wang S, et al. Exploring flavonoid intake and all-cause mortality in diverse health conditions: Insights from NHANES 2007-2010 and 2017-2018. Nutrition. 2024;127:112556.

665. Wang Y, et al. Diets with higher insulinaemic potential are associated with increased risk of overall and cardiovascular disease-specific mortality. Br J Nutr. 2022;128(10):2011-20.

666. Yamaoka T, et al. Association between Low Protein Intake and Mortality in Patients with Type 2 Diabetes. Nutrients. 2020;12(6).

667. Yeung SM, et al. Low Urinary Potassium Excretion Is Associated with Higher Risk of All-Cause Mortality in Patients with Type 2 Diabetes: Results of the Dutch Diabetes and Lifestyle Cohort Twente (DIALECT). Journal of Nutrition. 2022;152(12):2856-64.

668. Zhang X, et al. Associations of Nut Consumption with All-Cause Mortality among Individuals with Type 2 Diabetes. J Nutr. 2023;153(10):3003-11.

669. Zhang Y, et al. Association between spicy foods consumption and cardiovascular disease risk factors: Guangzhou Biobank Cohort Study. BMC public health. 2022;22(1):1278.

670. Zhang YF, et al. Potassium supplementation and long-term outcomes in chronic peritoneal dialysis patients with end-stage renal disease: a propensity score matching study. Renal failure. 2016;38(10):1594-600.

671. Zheng G, et al. Dietary Diversity and Inflammatory Diet Associated with All-Cause Mortality and Incidence and Mortality of Type 2 Diabetes: Two Prospective Cohort Studies. Nutrients. 2023;15(9).

672. Zheng G, et al. Fresh fruit, dried fruit, raw vegetables, and cooked vegetables consumption associated with progression trajectory of type 2 diabetes: a multi-state analysis of a prospective cohort. European Journal of Nutrition. 2024;63(5):1719-30.

673. Zhou L, et al. Co-exposure to multiple vitamins and the risk of all-cause mortality in patients with diabetes. Front Endocrinol (Lausanne). 2023;14:1254133.

674. Zhuang P, et al. Current Level of Fish Consumption is Associated with Mortality in Chinese but not US Adults: New Findings From Two Nationwide Cohort Studies With 14 and 9.8 Years of Follow-Up. Molecular nutrition & food research. 2018;62(8):e1700898.

675. Ahmed LHM, et al. Vitamin D(3) metabolite ratio as an indicator of vitamin D status and its association with diabetes complications. BMC Endocr Disord. 2020;20(1):161.

676. Bianchetti G, et al. Erythrocyte membrane fluidity: A novel biomarker of residual cardiovascular risk in type 2 diabetes. Eur J Clin Invest. 2024;54(3):e14121.

677. Burch J, Tort S. Can vitamin C supplementation help to prevent cardiovascular disease in men? Cochrane Clinical Answers. 2017.

678. Burch J, Tort S. How does increasing omega 6 fat intake affect mortality and development of cardiovascular disease? Cochrane Clinical Answers. 2018.

679. Burch J, Tort S. How do high and low concentrations of alpha linolenic acid (ALA) compare for primary and secondary prevention of cardiovascular disease? Cochrane Clinical Answers. 2020.

680. Burch J, Tort S. How do high and low concentrations of long-chain fatty acids (LCn3) compare for primary and secondary prevention of cardiovascular disease? Cochrane Clinical Answers. 2020.

681. Gant CM, et al. Higher dietary magnesium intake and higher magnesium status are associated with lower prevalence of coronary heart disease in patients with type 2 diabetes. Nutrients. 2018;10(3).

682. Kataja-Tuomola MK, et al. Effect of alpha-tocopherol and beta-carotene supplementation on macrovascular complications and total mortality from diabetes: results of the ATBC Study. Annals of medicine. 2010;42(3):178-86.

683. Leppälä JM, et al. Vitamin E and beta carotene supplementation in high risk for stroke. A subgroup analysis of the Alpha-Tocopherol, Beta-Carotene Cancer Prevention Study. Archives of Neurology. 2000;57(10):1503-9.

684. Myhre P, et al. Changes in EPA and DHA during supplementation with omega-3 fatty acids and incident cardiovascular events: secondary analysis from the OMEMI trial. European heart journal. 2021;42(SUPPL 1):2429.

685. Nishizaki Y, et al. Association between the docosahexaenoic acid to arachidonic acid ratio and acute coronary syndrome: a multicenter observational study. BMC Cardiovasc Disord. 2016;16(1):143.

686. Zhang HQ, et al. Association between composite dietary antioxidant index and stroke among individuals with diabetes. World J Diabetes. 2024;15(8):1742-52.

687. A˚kesson A, et al. Dietary exposure to polychlorinated biphenyls and incidence of myocardial infarction in men-a population-based prospective cohort study. Circulation. 2015;131.

688. A˚kesson A, et al. Combined low-risk dietary and lifestyle practice and risk of myocardial infarction in men. American Journal of Epidemiology. 2013;177:S130.

689. Adebamowo SN, et al. Dietary carbohydrate quantity and quality measures and risk of stroke. Circulation. 2016;133.

690. Agarwal S, et al. The association of calcium supplementation and incident cardiovascular events: Multi-ethnic study of atherosclerosis (MESA). Circulation. 2012;126(21).

691. Aggarwal S, et al. Water consumption does not influence all cause or cardiovascular mortality: A followup study of patients in the national health and nutrition examination survey. Circulation. 2012;126(21).

692. Akarolo-Anthony SN, et al. Serum magnesium and the risk of ischemic stroke among women. Circulation. 2013;127(12).

693. Akarolo-Anthony SN, et al. Dietary magnesium and the risk of stroke among women. Circulation. 2014;129.

694. Akter S, et al. Predictors of incident heart failure in community-dwelling older adults with diabetes mellitus. Diabetologia. 2010;53:S85.

695. Al Essa HB, et al. Carbohydrate quality and quantity and risk of coronary heart disease among us women. Circulation. 2016;133.

696. Al-Ramady O, et al. Dairy consumption and risk of cardiovascular disease and mortality in the million veteran program. Circulation. 2020;141(SUPPL 1).

697. Al-Shaar L, et al. Cumulative Consumption of Sulfur Amino Acids and Risks of Cardiovascular Disease and Mortality; Analysis of Two Prospective Cohort Studies. Circulation. 2022;145.

698. AlEssa HB, et al. Carbohydrate quality and quantity and risk of coronary heart disease among US women and men. Am J Clin Nutr. 2018;107(2):257-67.

699. Baden MY, et al. Association of changes in plant-based diet indices with total and cause-specific mortality. Circulation. 2019;139.

700. Benderly M, et al. The association between ideal cardiovascular health behavior and long-term risk profile and outcomes. European Heart Journal. 2019;40:3841.

701. Bergkvist C, et al. Dietary exposure to polychlorinated biphenyls and risk of myocardial infarction in women-a population-based prospective cohort study. American Journal of Epidemiology. 2013;177:S158.

702. Bertoia ML, et al. Estimated risk of coronary heart disease associated with the replacement of various foods with vegetables in two large prospective cohorts. Circulation. 2016;133.

703. Beulens JWJ, et al. Dietary fat intake in low-carbohydrate diets and subsequent mortality and weight change in type 2 diabetes. Diabetologia. 2014;57(1):S311.

704. Bhave VM, et al. Ultra-Processed Food Intake Predicts Stroke Risk in the Reasons for Geographic and Racial Differences in Stroke (REGARDS) Study. Stroke. 2024;55.

705. Bianco H, et al. Predictors of long-term cardiovascular outcomes in patients with type 2 diabetes mellitus. European Heart Journal. 2012;33:893.

706. Bloom A, et al. High salt intake and its effects on non-hypertensive complications in geriatric patients with type 2 diabetes mellitus. Clinical Nutrition. 2015;34:S80.

707. Bodar V, et al. Coffee consumption and risk of heart failure in the physicians' health study. Circulation. 2018;137.

708. Bonaccio M, et al. Ultra-processed Food Intake and All-cause and Cause-specific Mortality in Subjects with Type 2 Diabetes: Longitudinal Findings from the Moli-sani Study. Circulation. 2022;145.

709. Bonaccio M, et al. Combined impact of healthy lifestyle factors on survival in high risk groups: Results from the Moli-sani study. European Journal of Preventive Cardiology. 2018;25(2):S8-S9.

710. Bonaccio M, et al. Increased adherence to a Mediterranean dietary pattern is associated with lower incidence of coronary artery disease and stroke: Prospective findings from the MOLI-SANI study. European Journal of Preventive Cardiology. 2015;22(1):S5.

711. Bonaccio M, et al. Adherence to the traditional Mediterranean diet and mortality in subjects with diabetes. Prospective results from the MOLI-SANI study. Thrombosis Research. 2014;134:S35.

712. Bonaccio M, et al. Higher adherence to Mediterranean diet is associated with lower risk of overall mortality in subjects with cardiovascular disease: Prospective results from the MOLI-SANI study. European Heart Journal. 2016;37:555-6.

713. Bonaccio M, et al. Higher adherence to the traditional Mediterranean diet is associated with lower cardiovascular risk and all-cause mortality in the elderly: Prospective findings from the Moli-sani study. European Journal of Preventive Cardiology. 2017;24(1):S8.

714. Booth JN, et al. Healthy lifestyle factors are uncommon and associated with reduced risk of cardiovascular disease and mortality in candidates for primary prevention with statin therapy. Circulation. 2015;131.

715. Brar SS, et al. The association between dietary fiber intake and clinical outcomes in CKD: A report from the chronic renal insufficiency cohort (CRIC). Journal of the American Society of Nephrology. 2020;31:190.

716. Buendia JR, et al. Increased yogurt intake lowers risk of cardiovascular disease among middle-aged adults with high blood pressure. FASEB Journal. 2017;31(1).

717. Bui L, et al. Planetary Health Diet Index and Risk of Total and Cause-Specific Mortality in Two Prospective Cohort Studies. Current Developments in Nutrition. 2023;7.

718. Burger K, et al. Dietary glycemic load and glycemic index and risk of coronary heart disease and stroke. Annals of Nutrition and Metabolism. 2011;58:309.

719. Campmans-Kuijpers MJE, et al. Dietary protein intake in low-carb diets and subsequent weight change and mortality in type 2 diabetes. Diabetologia. 2014;57(1):S312.

720. Cespedes S, et al. Dietary sodium to potassium ratio and risk of stroke in a multi-ethnic urban dwelling population: The Northern Manhattan study. Circulation. 2016;133.

721. Chen Z, et al. Dietary Phytoestrogens and Total and Cause-Specific Mortality: Results From Two Prospective Cohort Studies. Current Developments in Nutrition. 2022;6:890.

722. Chiusolo S, et al. The ratio between N-3 and N-6 polynsaturated fatty acids in the adipose tissue is more predictive of myocardial infarction than absolute levels of N-3 fatty acids: results from the Danish Diet, Cancer and Health cohort study. European heart journal, supplement. 2020;22(SUPPL N):N24.

723. Chiuve S, et al. Do nutrition rating systems promote a healthy diet? An evaluation of the Overall Nutritional Quality Index (ONQI) and risk of chronic disease. FASEB Journal. 2010;24.

724. Chiuve SE, et al. Derivation and validation of a lifestyle CVD risk score for the prediction of CVD among middle-aged women and men. Circulation. 2013;127(12).

725. Chiuve SE, et al. Dietary and plasma magnesium and risk of coronary heart disease among women. Circulation. 2012;125(10).

726. Choi HS, et al. Relationships of 24-hour urinary phosphate excretion and serum phosphate with clinical outcomes in CKD: From the Korean cohort study for outcome in patients with chronic kidney disease (Knowckd). Journal of the American Society of Nephrology. 2017;28:995.

727. Chrysohoou C, et al. Adherence to Mediterranean diet protects against cardiovascular disease independently of creatinine clearance rate: The 10-year (2002-12) Follow-up of Attica study. European Heart Journal. 2015;36:474.

728. Cidade-Rodrigues C, et al. The prognostic impact of magnesium in acute heart failure is different according to diabetes mellitus status. European Journal of Heart Failure. 2021;23(SUPPL 2):190.

729. Cole N, et al. Plasma sodium concentration and the risk of cardiovascular disease: A large community-based cohort study. Journal of Hypertension. 2017;35:e2.

730. Cole N, et al. Serum sodium concentration and the risk of cardiovascular disease: A large community-based cohort study. Journal of Human Hypertension. 2017;31(10):673.

731. Cole NI, et al. The association between serum sodium and potassium concentration and the risk of cardiovascular disease: A large community-based cohort study. Journal of the American Society of Nephrology. 2017;28:397.

732. Colin-Ramirez E, et al. Dietary sodium intake and outcomes: a secondary analysis from SODIUM - HF. European heart journal. 2023;44.

733. Collin L, et al. Sugar-sweetened beverage and food intake and mortality risk among U.S. adults. Circulation. 2018;137.

734. Costanzo S, et al. Total antioxidant capacity of diet and all-cause mortality in healthy elderly of the MOLI-SANI project. European Journal of Preventive Cardiology. 2014;21(1):S116.

735. Crowe FL, et al. Risk of ischaemic heart disease among British vegetarians and non-vegetarians: Results from the EPIC-Oxford cohort study. Proceedings of the Nutrition Society. 2012;71.

736. Cruijsen E, et al. Vitamin D status and 12-year mortality risk after myocardial infarction. European Heart Journal. 2023;44.

737. Cruijsen E, et al. Diet quality and long-term cardiovascular mortality after myocardial infarction in the Alpha Omega Cohort. European Heart Journal. 2023;44.

738. De Goede J, et al. Dietary intake of saturated fat by food source and incident coronary heart disease: The zutphen elderly study. Circulation. 2015;131.

739. De Oliveira Otto MC, et al. Biomarkers of dairy fatty acids and incidence of cardiovascular disease in the multi-ethnic study of atherosclerosis (MESA). Circulation. 2013;127(12).

740. Degerud EM, et al. Vitamin D status and mortality in patients with stable angina. Clinical Nutrition. 2014;33:S15.

741. Deschasaux M, et al. Prospective associations between the nutritional quality of foods consumed (graded by the FSAm-NPS underlying the Nutri-Score) and mortality in Europe. Proceedings of the Nutrition Society. 2020;79(OCE2).

742. Di Castelnuovo AF, et al. Prediction of all-cause mortality in diabetic patients. Circulation. 2019;139.

743. Diaz-Gutierrez J, et al. A score of traditional and novel lifestyles for optimal cardiovascular health: The SUN cohort. European Heart Journal. 2017;38:241.

744. Ditah I, et al. Low 25-hydroxy vitamin d levels and mortality among U.S. Adults: Results of NHANES 18-year mortality-linked data. American Journal of Gastroenterology. 2014;109:S636.

745. Djoussé L. Association of modifiable lifestyle factors and risk of total and cardiovascular mortality among older us male physicians. Cardiology (Switzerland). 2015;131:333.

746. Djousse L, et al. Plasma free fatty acids are positively associated with incident heart failure in the cardiovascular health study. Circulation. 2013;127(12).

747. Djousse L, Gaziano JM. Statin modifies the association of fish consumption with risk of heart failure: The physicians' health study. Cardiology (Switzerland). 2017;137:257.

748. Djousse L, et al. Egg consumption is positively associated with ischemic stroke: The million veteran program. Circulation. 2020;141(SUPPL 1).

749. Djousse L, et al. Nut consumption is associated with a lower risk of death among us male physicians. Circulation. 2014;129.

750. Djousse L, et al. Fried food consumption is associated with a higher risk of heart failure among us male physicians. Circulation. 2015;131.

751. Djousse L, et al. Plasma phospholipid concentration of palmitoleic acid is associated with an increased risk of heart failure in male physicians. Circulation. 2012;125(10).

752. Dos Santos ALT, et al. Association between nutritional recommendations for cardiovascular disease and a lower incidence of cardiac events in patients with type 2 diabetes. Diabetes. 2010.

753. Du H, et al. Fresh fruit consumption in relation to mortality and incidence of vascular events among 26,000 individuals with diabetes: A 7-year prospective study. European Heart Journal. 2015;36:884-5.

754. Du H, et al. Fresh fruit consumption in relation to incident diabetes and diabetic vascular complications: Findings from the China Kadoorie Biobank Study. The Lancet Diabetes and Endocrinology. 2016;4(SPEC. ISSUE 3):S12.

755. Eaton CB, et al. High intake of dietary protein is associated with increased risk of heart failure with preserved ejection fraction. Circulation. 2017;135.

756. Ekinci EI, et al. Dietary salt intake and mortality in patients with type 2 diabetes. Diabetes. 2010.

757. Ekinci EI, et al. Relationship between serial 24h urinary sodium excretion and mortality in type 2 diabetes. Diabetes. 2013;62:A401.

758. Engelen AI, et al. Vitamin k intake and risk of coronary heart disease and stroke in the Rotterdam study. Circulation. 2015;131.

759. Fan W, et al. Multifactorial Risk Control in Atherosclerotic Cardiovascular Disease Among Patients With Type 2 Diabetes Mellitus: Findings From the UK Biobank. Circulation. 2024;149.

760. Fang Z, et al. Association of Ultra-Processed Food Intake With All-Cause and Cause-Specific Mortality. Current Developments in Nutrition. 2023;7.

761. Floegel A, et al. Coffee consumption and risk of chronic disease in the European Prospective Investigation into Cancer and Nutrition (EPIC)-Germany study. American Journal of Clinical Nutrition. 2012;95(4):901-8.

762. Fortin E, et al. High mannose correlates with surrogate indexes of insulin resistance and predicts cardiovascular events independently of glycaemic status and traditional risk factors. Diabetologia. 2023;66:S493.

763. Fung T, et al. Mediterranean diet and incidence and mortality of coronary heart disease and stroke in women. The FASEB Journal. 2009;23(S1).

764. Gadiraju T, et al. Fried food consumption is associated with a higher risk of cardiovascular mortality in the physicians' health study. Journal of the American College of Cardiology. 2016;67(13):1913.

765. Galbete Ciaurriz C, et al. Nordic diet, mediterranean diet, and the risk of chronic diseases: The epic-potsdam study. Annals of Nutrition and Metabolism. 2017;71:320.

766. Ganbaatar G, et al. The association of dietary Inflammatory Index with long-term all-cause and cardiovascular mortality risk: NIPPON DATA80. Annals of Nutrition and Metabolism. 2023;79:336.

767. Gao M, et al. Associations between dietary patterns with CVD and total mortality among 118 554 adults from the UK Biobank: A prospective cohort study. Obesity Reviews. 2020;21(SUPPL 1).

768. Gardener H, et al. Northern manhattan study (NOMAS). Stroke. 2011;42(3):e50.

769. Gardener H, et al. Soda consumption and risk of vascular events in the northern manhattan study. Stroke. 2011;42(3):e273.

770. Georgousopoulou EN, et al. Adherence to mediterranean is the most important protector against the development of fatal and non-fatal cardiovascular event: 10-year follow-up (2002-12) of the Attica study. Journal of the American College of Cardiology. 2015;65(10):A1449.

771. Glenn AJ, et al. Association of the Portfolio Diet with Total and Cardiovascular Disease Mortality in the Women's Health Initiative. Annals of Nutrition and Metabolism. 2023;79:337-8.

772. Gonzalez MÁM, et al. Mediterranean diet and health outcomes in the SUN cohort. European Journal of Clinical Investigation. 2018;48:178.

773. Gribbin S, et al. Higher Dietary Carbohydrate Intake and Not Saturated Fat is Inversely Associated With Cardiovascular Disease in Australian Women. Heart Lung and Circulation. 2021;30:S279-S80.

774. Guo J, et al. Egg consumption and cardiovascular disease events-evidence from the Caerphilly prospective cohort study. Proceedings of the Nutrition Society. 2015;74(OCE5).

775. Han H, et al. Adherence to healthy lifestyle is associated with lower total and cause-specific mortality risk among patients with diabetes: The uk biobank study. Circulation. 2021;144(SUPPL 1).

776. Hansen-Krone IJ, et al. Impact of dietary patterns on the risk of myocardial infarction and venous thromboembolism. the tromso study 1994-2005. Journal of Thrombosis and Haemostasis. 2011;9:24.

777. Harrington DM, et al. Ideal cardiovascular health criteria are inversely related to cardiovascular disease and all-cause mortality in a sample of canadian adults. Circulation. 2013;127(12).

778. Harris K, et al. Plasma fatty acids and the risk of vascular and mortality outcomes in individuals with type 2 diabetes: Results from the advance study. Journal of Hypertension. 2021;39(SUPPL 1):e45.

779. Harvey NC, et al. Calcium supplementation is not associated with ischaemic heart disease regardless of cardiac risk factors: The uk biobank cohort. Osteoporosis International. 2017;28:S258.

780. Haugsgjerd TR, et al. Association between dietary fat intake and coronary heart disease in middle-aged and senior adults. the Hordaland Health Studies (HUSK). European Journal of Preventive Cardiology. 2017;24(1):S74.

781. Heffron S, et al. Increasing frequency of fruit and vegetable consumption is associated with lower prevalence of peripheral arterial disease in a very large community cohort. Journal of the American College of Cardiology. 2014;63(12):A2048.

782. Heffron SP, et al. Frequency of nut consumption is associated with reduced prevalence of peripheral arterial disease in a very large community cohort. Circulation. 2014;129.

783. Hefzy HM, et al. Effect of caffeine consumption on carotid intimal media thickness and risk of stroke: The framingham offspring study. Stroke. 2011;42(3):e191.

784. Hirahatake KM, et al. Diet quality and cardiovascular disease risk in postmenopausal women with type 2 diabetes: The women's health initiative. Circulation. 2017;135.

785. Horikawa C, et al. Sodium intake and incidence of diabetes retinopathy in elderly patients with type 2 diabetes: Analysis of data from the japanese elderly diabetes intervention study (J-edit). Diabetes. 2021;70(SUPPL 1).

786. Horikawa C, et al. Impact of sodium intake on the risk of cardiovascular disease in patients with type 2 diabetes: Analysis of data from the Japan diabetes complications study and the Japanese elderly diabetes intervention trial. Diabetes. 2021;70(SUPPL 1).

787. Horikawa C, et al. Higher dietary intake of vitamin d is associated with lower incidence of diabetic nephropathy in Japanese patients with type 2 diabetes. Diabetes. 2018;67:A418.

788. Horikawa C, et al. Effect of Dietary Potassium Intake and Its Interaction with Sodium Intake on Risk of Developing Cardiovascular Disease (CVD) in Persons with Type 2 Diabetes (T2D)-Japan Diabetes Complication and Its Prevention Prospective Study (JDCP Study). Diabetes. 2023;72.

789. Horikawa C, et al. Is carbohydrate intake associated with the incidence of diabetes complications? Japan Diabetes Complication Study (JDCS). Diabetes. 2016;65:A198.

790. Horikawa C, et al. Dietary sodium intake and incidence of diabetic complications in Japanese patients with type 2 diabetes: Analysis of the Japan Diabetes Complications Study (JDCS). Diabetologia. 2013;56:S104.

791. Horikawa C, et al. Meat intake and incidence of cardiovascular disease in japanese patients with type 2 diabetes: Japan diabetes complication study (JDCS). Diabetes. 2015;64:A209-A10.

792. Howlett C, et al. Associations of Urinary Zinc With Incident Peripheral Arterial Disease and Amputation in the Strong Heart Study. Circulation. 2024;149.

793. Hu EA, et al. Adherence to the healthy eating index-2015 may reduce the risk of incident cardiovascular disease, cardiovascular disease mortality, and all-cause mortality. Circulation. 2019;139.

794. Hu T, et al. Low-carbohydrate dietary pattern and mortality in us adults: The third national health and nutritional examination survey (NHANES III). Circulation. 2013;127(12).

795. Hu Y, et al. Low-Carbohydrate Diets Score and Mortality Among Adults with Incident Type 2 Diabetes. Current Developments in Nutrition. 2022;6:907.

796. Huang NK, et al. Serum non-esterified fatty acids and risk of incident stroke in older adults: The cardiovascular health study. Circulation. 2020;141(SUPPL 1).

797. Huang T, et al. Contribution of aha life's simple 7 to sex differences in the incidence of coronary heart disease and stroke. Circulation. 2020;141(SUPPL 1).

798. Iacoviello L, et al. The role of diet in the increase of cardiovascular risk in the prospective cohort of Moli-Sani Study. Annals of Nutrition and Metabolism. 2023;79:187-8.

799. Ibrahim M, et al. THE EMPIRICAL DIETARY INFLAMMATORY PATTERN SCORE AND THE RISK OF CARDIOVASCULAR DISEASE IN PATIENTS WITH NAFLD. Hepatology. 2022;76:S713-S4.

800. Isiozor N, et al. Change in Cardiovascular Health, Cardiovascular Disease, and Mortality in Patients With Diabetes. Circulation. 2022;146.

801. Joosten M, et al. Urinary magnesium excretion and risk of cardiovascular disease in the general population. Kidney Research and Clinical Practice. 2012;31(2):A40.

802. Judd SE, et al. Vitamin D deficiency is associated with stroke in black and white participants of the regards study. Stroke. 2014;45.

803. Julian-Serrano S, et al. Does Socioeconomic Deprivation Modify the Association Between Healthy Eating Index-2015 and Mortality in the NIH-AARP Diet and Health Study? Current Developments in Nutrition. 2023;7.

804. Kang YU, et al. Relationships of Dietary Phosphate Intake and Serum Phosphate with Clinical Outcomes in Chronic Kidney Disease Stages 3-5: Findings from the KoreaN Cohort Study for Outcome in Patients with Chronic Kidney Disease (KNOW-CKD) Study. Journal of the American Society of Nephrology. 2016;27:809A.

805. Kaufman A, et al. Identifying dietary and nutritional risk factors for symptomatic peripheral arterial disease using the UK biobank cohort study. Vascular Medicine. 2020;25(3):NP5.

806. Kelly R, et al. Associations between Types and Sources of Dietary Carbohydrates and Cardiovascular Disease Risk in 110,505 UK Biobank Study Participants. Current Developments in Nutrition. 2022;6:912.

807. Khan SS, et al. Association of dietary patterns and lifetime risk of heart failure: The cardiovascular disease lifetime risk pooling project. Circulation. 2019;139.

808. Kiage JN, et al. Trans-fat intake and incidence of stroke in the reasons for geographical and racial differences in stroke cohort. Circulation. 2013;128(22).

809. Kippler M, et al. Dietary polychlorinated biphenyls and long-chain omega-3 fish fatty acids exposures and risk of heart failure. Circulation. 2018;137.

810. Kong SH, et al. Dietary calcium intake and risk of cardiovascular disease, stroke, and fracture in a low calcium intake population: A prospective community-based cohort study. Endocrine Reviews. 2016;37(2).

811. Kota H, et al. Dietary magnesium intake, risk of kidney stone, and survival in the women's health initiative (WHI). Journal of the American Society of Nephrology. 2020;31:792.

812. Lajous M, et al. Hypothetical interventions on fish consumption in mid and later life and the risk of coronary heart disease in women: An application of the parametric G-formula. Circulation. 2012;125(10).

813. Lee CD. Combined effects of lifestyle factors on chronic disease mortality in men and women. Circulation. 2013;127(12).

814. Lee CD. Effects of healthy lifestyle behaviors on lifetime risk of chronic disease mortality and life expectancy in men and women. Circulation. 2014;129.

815. Lemaitre RN, et al. Plasma phospholipid very long chain saturated fatty acids and healthy aging in older adults: The cardiovascular health study. Circulation. 2018;137.

816. Lemaitre RN, et al. Circulating epoxyeicosatrienoic acids, diabetes, and cardiovascular disease. Diabetes. 2019;68.

817. Lentjes MAH, et al. Total (food and supplement) n-3 PUFA intake is associated with lower Coronary Heart Disease mortality, independently of fish intake. Proceedings of the Nutrition Society. 2016;75(OCE1):E42.

818. Li S, et al. Dietary glycemic index and glycemic load and risk of coronary heart disease in a prospective study among us male health professionals. Circulation. 2012;125(10).

819. Li Y, et al. Association of Ultra-Processed Food Consumption With Risk of Cardiovascular Disease Among Individuals With Type 2 Diabetes in the UK Biobank. Current Developments in Nutrition. 2023;7.

820. Liebeskind DS, et al. Coffee protects? Reduced prevalence of stroke with more cups of coffee. Stroke. 2009;40(4):e191.

821. Lim LL, et al. High serum branched-chain amino acids level independently predicts incident heart failure-the Hong Kong diabetes register. Diabetes. 2018;67:A122.

822. Lim WH, et al. Total fluid intake and risk of mortality in older women. Nephrology. 2015;20:27.

823. Liu B, et al. Lignan Intake and Mortality Among Adults With Incident Type 2 Diabetes. Circulation. 2024;149.

824. Liu G, et al. Adherence to a healthy lifestyle in relation to cardiovascular disease incidence and mortality among adults with type 2 diabetes. Circulation. 2018;137.

825. Liu L. Micronutrients, inflammatory biomarkers, and risk of cardiovascular disease and all-cause mortality in the United States: Implications for a healthier diet and longevity. Circulation. 2010;122(2):e85-e6.

826. Liu Q, et al. Plasma phospholipid fatty acid and coronary heart disease risk. Circulation. 2016;133.

827. Lo A, et al. Tea is the Key! Green Tea Intake and All-Cause Mortality Over Five Decades in Japanese-American Men: The Kuakini Honolulu Heart Program. Journal of the American Geriatrics Society. 2022;70(SUPPL 1):S131-S2.

828. Loftfield E, et al. Ultra-Processed Food Intake and Mortality in the NIH-AARP Diet and Health Study. 2024.

829. Loomba RS, et al. Daily coffe consumption does not impact all-cause or specific cardiovascular mortality: Findings from the national health and nutrition examination survey. Circulation. 2012;126(21).

830. Lopez FL, et al. Serum phosphorus levels and the incidence of atrial fibrillation: The atherosclerosis risk in communities (ARIC) study. Circulation. 2012;125(10).

831. Magnusson M, et al. High levels of arginine, citrulline and ADMA are independent predictors of cardiovascular disease. European Heart Journal. 2013;34:1058-9.

832. Mahbub HM, et al. Dietary Consumption of Potatoes and Incidence of Cardiovascular Disease. Circulation. 2024;149.

833. Malik VS, et al. Sugar sweetened and artificially sweetened beverages and risk of mortality in us adults. Circulation. 2014;129.

834. Maria Laura Bonaccio M, et al. Adherence to the mediterranean diet is associated with reduced overall mortality in subjects with diabetes. Prospective results from the Moli-sani study. European Journal of Preventive Cardiology. 2014;21(1):S55.

835. Marklund M, et al. Serum pentadecanoic acid, a biomarker of dairy fat intake, is associated with lower risk of incident cardiovascular disease and all-cause mortality in Swedish men and women. Annals of Nutrition and Metabolism. 2017;71:322-3.

836. Mazidi M, et al. Association of types of dietary fats and all-cause and cause-specific mortality: A prospective cohort study and meta-analysis of prospective studies with 1,148,117 participants. European Heart Journal. 2019;40:7.

837. Mertens E, et al. Pulse pressure trajectories in relation to cardiovascular mortality and dietary protein intake: The Zutphen Study. Proceedings of the Nutrition Society. 2015;74(OCE5).

838. Metcalf PA, Scragg RK. Vitamin D and parathyroid hormone as predictors of CVD morbidity and CVD and all-cause mortality. Circulation. 2013;128(22).

839. Miyazawa I, et al. Relationship of dietary carbohydrate and fiber intake to risk of cardiovascular disease mortality in Japanese: NIPPON DATA80. Circulation. 2017;135.

840. Mölenberg FJ, et al. Dietary fatty acids and coronary heart disease in mortality in the alpha omega cohort. Circulation. 2017;135.

841. Mostofsky E, et al. Chocolate intake and incidence of heart failure: A population-based, prospective study of middle-aged and elderly women. American Journal of Epidemiology. 2010;171:S6.

842. Motonaga HM, et al. Low dietary vitamin D as a predictor of 34-year incident stroke: The Honolulu heart program. Journal of the American Geriatrics Society. 2010;58:S64.

843. Nagai M, et al. Impact of total energy intake on stroke mortality is greater than that of coronary heart disease: A 24-year follow-up of representative Japanese (nippon data80), 1980-2004. Stroke. 2013;44(2).

844. Nestel P. Dietary fatty acids including TFA and heart disease. Atherosclerosis Supplements. 2009;10(2).

845. Neves JS, et al. Caffeine consumption and mortality in diabetes: An analysis of NHANES 1999-2010. Diabetologia. 2017;60(1):S389-S90.

846. Ninomiya T, et al. Relationship between the ratio of serum eicosapentaenoic acid to arachidonic acid and the risk of death: The hisayama study. Circulation. 2011;124(21).

847. Nothlings U, et al. Healthy living and mortality among adults with diabetes: Findings from the European prospective investigation into cancer and nutrition-potsdam study. Diabetes. 2009;58.

848. Nuzzo V, et al. Analysis of 25-OH vitamin D in patients with type 2 diabetes mellitus. Italian Journal of Medicine. 2012;6(1):102.

849. Odegaard AO, et al. Dietary patterns and risk of all-cause and cause-specific mortality in chinese men and women: The singapore chinese health study. Circulation. 2012;125(10).

850. Odegaardl AO, et al. Diet beverage intake is positively associated with incident coronary heart disease in people with type 2 diabetes. Circulation. 2018;137.

851. Oikonomou E, et al. Dietary consumption of olive oil and cardiovascular outcome in patients with coronary artery disease. Journal of the American College of Cardiology. 2017;69(11):146.

852. Oikonomou E, et al. Olive oil consumption can favorably affect cardiovascular prognosis in patients after percutaneous coronary intervention. European Heart Journal. 2015;36:475.

853. Owen A, et al. Dietary polyunsaturated fat intake and risk of cardiovascular mortality in the ausdiab cohort. Heart Lung and Circulation. 2012;21:S307-S8.

854. Pan A, et al. Red meat consumption and mortality: Results from two cohorts of American adults. Circulation. 2011;124(21).

855. Panagiotakos DB, et al. The association between adherence to the mediterranean diet and indices of glucose homoeostasis in predicting cardiovascular disease events; 10-year follow-up (2001-2011) of the Attica study. European Heart Journal. 2014;35:725-6.

856. Panagiotakos DB, et al. The association between adherence to the mediterranean diet and diabetes mellitus on the 10-year (2004-2014) acute coronary syndrome (ACS) prognosis; the Greecs study. European Heart Journal. 2015;36:749.

857. Pearson KE, et al. Associations of dietary patterns and cardiovascular disease risk do not differ by diabetes status. Circulation. 2014;129.

858. Petersen K, et al. The combined impact of five lifestyle factors on all-cause, cancer, and cardiovascular mortality-a prospective cohort study among danes. Annals of Nutrition and Metabolism. 2013;63:1131.

859. Petrone AB, et al. Adherence to healthy lifestyle factors is associated with a lower risk of death among us male physicians with type 2 diabetes. Circulation. 2015;131.

860. Pfister R, et al. Urinary sodium excretion and risk of heart failure in men and women in the EPIC-Norfolk study. European Heart Journal. 2013;34:463.

861. Qi L, et al. Adherence to healthy lifestyle and cardiovascular diseases in chinese. Circulation. 2016;134.

862. Qi L, et al. Consumption of whole grain and cereal fiber with total and cause-specific mortality: Prospective analysis of 367,442 individuals. FASEB Journal. 2014;28(1).

863. Qi L, et al. Ready to eat cereal consumption with total and cause-specific mortality: Prospective analysis of 367,442 individuals. FASEB Journal. 2014;28(1).

864. Qi Q, et al. Consumption of branched chain amino acids and risk of coronary heart disease in us men and women. Circulation. 2013;127(12).

865. Qiu Z, et al. Associations of Habitual Calcium Supplementation With Risk of Cardiovascular Disease and Mortality in Individuals With and Without Diabetes. Current Developments in Nutrition. 2023;7.

866. Qureshi AI, Suri MFK. High intake of caffeine use in protective in survivors of stroke and myocardial infarction: The results of third national health and nutrition examination survey mortality follow-up study. Stroke. 2012;43(2).

867. Raimann JG, et al. Association of serum and dialysate sodium and sodium gradient with mortality in incident hemodialysis patients: Results from the international monitoring dialysis outcomes (Mondo) initiative. Nephrology Dialysis Transplantation. 2014;29:iii66.

868. Rautiainen S, et al. Fruit and vegetable consumption and the risk of heart failure in women. Circulation. 2014;129.

869. Rautiainen S, et al. Longitudinal changes in berry intake and the risk of cardiovascular disease in women. Circulation. 2017;135.

870. Rousseau MF, et al. High ferritin levels confer lower cardiovascular risk in type 2 diabetes. Journal of the American College of Cardiology. 2010;55(10):A157.E1466.

871. Saber H, et al. Circulating phospholipid N-3 polyunsaturated fatty acids and incident atherothrombotic and cardioembolic ischemic stroke in 3 large us cohorts. Circulation. 2016;133.

872. Saeed O, et al. Debunking the myth of regular soda beverages use and the risk of incident cardiovascular events. Stroke. 2018;49.

873. Satija A, et al. Plant-based diets and the risk of coronary heart disease in US adults. FASEB Journal. 2017;31(1).

874. Saulnier P, et al. Association between sodium and potassium urinary concentrations and cardiovascular and renal complications in a French cohort of type 2 diabetes patients. Diabetologia. 2013;56:S189.

875. Saulnier PJ, et al. Association between detetary sodium intake and cardiovascular complications in a French cohort of type 2 diabetes patients. Diabetes. 2013;62:A399-A400.

876. Sawicki C, et al. Planetary Health Diet and Risk of Incident Cardiovascular Disease: Findings From Two Cohorts of US Women. Current Developments in Nutrition. 2023;7.

877. Saydah S, et al. Healthy lifestyle behaviours and risk of mortality among adults with and without diabetes in the U.S. Canadian Journal of Diabetes. 2009;33(3):191.

878. Schartum-Hansen H, et al. Realtionship between plasma choline and betaine levels and risk of acute myocardial infarction in patients with stable coronary heart disease. European Heart Journal. 2012;33:132.

879. Schoufour J, et al. Adherence to the 2015 DUTCH dietary guidelines and its associations with mortality and incidence of non-communicable diseases in the rotterdam study. Annals of Nutrition and Metabolism. 2017;71:355-6.

880. Schwartz B, et al. DIET, ANGIOGRAPHIC CORONARY DISEASE, AND CARDIOVASCULAR OUTCOMES IN WOMEN. Journal of the American College of Cardiology. 2022;79(9):1598.

881. Seguro F, et al. Non adherence to cardiovascular preventive guidelines is associated with all-cause and cardiovascular mortality. European Heart Journal. 2014;35:195-6.

882. Shalaeva E, et al. Impact of compliance with lifestyle recommendations and medication adherence on 1-year prevention of major cardiovascular events in diabetic patients undergoing partial foot amputation. European Journal of Preventive Cardiology. 2021;28(SUPPL 1):i126.

883. Shalaeva E, et al. Tenfold risk increase of major cardiovascular events after high limb amputation with non-compliance for secondary prevention measures. European Heart Journal. 2016;37:1343.

884. Shan Z, et al. Healthy eating patterns and risk of cardiovascular disease: Results from three large prospective cohort studies. Circulation. 2020;141(SUPPL 1).

885. Shikany JM, et al. Specific dietary patterns are associated with risk of acute coronary heart disease in the reasons for geographic and racial differences in stroke (regards) study. Circulation. 2013;127(12).

886. Shin HJ, et al. The association between diet quality and mortality in incident type 2 diabetic women. Circulation. 2013;128(22).

887. Shin HJ, et al. The association between diet quality after diabetes diagnosis and major cardiovascular events in women with type 2 diabetes mellitus. Circulation. 2014;130.

888. Simon J, et al. Association of daily coffee consumption with cardiovascular health-results from the UK Biobank. European Heart Journal. 2021;42(SUPPL 1):2416.

889. Sonestedt E, et al. Carbohydrate-rich foods and risk of cardiovascular disease in the Malmö diet and cancer cohort. European Journal of Epidemiology. 2013;28(1):S184.

890. Sotos-Prieto M, et al. Association between a 20-year cvd-risk score based on modifiable lifestyles and total and cause specific mortality among us men and women. Circulation. 2017;135.

891. Sotos-Prieto M, et al. Application of a lifestyle-based score to predict cardiovascular risk in African americans: The jackson heart study. Circulation. 2021;143(SUPPL 1).

892. Spence JD, et al. Effect of dietary cholesterol and egg consumption on mortality and cardiovascular risk in the regards study. Stroke. 2015;46.

893. Steinhaus D, et al. Chocolate intake and incidence of heart failure: Findings from the cohort of swedish men (COSM). Circulation. 2014;129.

894. Suri M, Qureshi A. High intake of caffeine use in protective in survivors of stroke and myocardial infarction: The results of third national health and nutrition examination survey mortality follow-up study. Neurology. 2012;78(1).

895. Suzuki S, et al. Plasma branched chain amino acids levels and cardiovascular mortality in hemodialysis patients. Circulation. 2017;136.

896. Takata Y, et al. Dairy consumption and total and causespecific mortality in the Southern Community Cohort Study. FASEB Journal. 2017;31(1).

897. Tanaka S, et al. Intakes of dietary fiber, vegetables, and fruits and incidence of cardiovascular disease in Japanese patients with type 2 diabetes. Diabetes. 2013;62:A398.

898. Tasevska N, et al. Added sugars in diet and risk of total and cause-specific mortality in a large US cohort. FASEB Journal. 2010;24.

899. Taveira TH, et al. The relationship between dietary magnesium intake and heart failure hospitalizations in african american adults: The jackson heart study. Circulation. 2014;130.

900. Tikhonoff V, et al. Prognostic cut-off values of caffeine and cardiovascular events in a cohort of unselected men and women from general population. European Heart Journal. 2022;43:2436.

901. Van Maaren J, et al. VARIABILITY OF 24-HOUR URINE SODIUM EXCRETION AND LONG-TERM CARDIOVASCULAR AND RENAL OUTCOME. Journal of Hypertension. 2022;40:e46.

902. Vendeville ACJ, et al. Risk of urinary sodium excretion and sodium-to-potassium ratio on major cardiovascular events in patients with clinical manifest vascular disease. European Heart Journal. 2018;39:266-7.

903. Virtanen HE, et al. Animal & dairy protein intakes associate with increased risk of heart failure in men: The kuopio ischaemic heart disease risk factor study. Circulation. 2017;135.

904. Virtanen JK, et al. Saturated fat intake by food source and risk of incident coronary heart disease in men: The kuopio ischaemic heart disease risk factor study. Circulation. 2017;135.

905. Virtanen JK, et al. Dietary fatty acids and the risk of fatal and non-fatal coronary heart disease in middle-aged or older men: The kuopio ischaemic heart disease risk factor study. Circulation. 2013;127(12).

906. Vyas A, et al. Diet drink consumption and the risk of cardiovascular events: A report from the women's health initiative. Journal of the American College of Cardiology. 2014;63(12):A1290.

907. Walker ME, et al. Proteomic Signature of Healthy Dietary Patterns and Risk of Cardiovascular Disease in the Framingham Heart Study. Circulation. 2022;145.

908. Walsh J, et al. Serum calcium as a predictor of cardiovascular disease in the busselton health study. Osteoporosis International. 2011;22:S624.

909. Wan D, et al. DIETARY INTAKE AND CARDIOVASCULAR OUTCOMES IN PATIENTS WITH CHRONIC VASCULAR DISEASE IN THE COMPASS TRIAL. Canadian journal of cardiology. 2022;38(10):S217-S8.

910. Wang DD, et al. Higher fruit and vegetable intake was associated with lower total and cause-specific mortality in a nonlinear dose-response manner. Circulation. 2018;137.

911. Wang DD, et al. Specific dietary fats in relation to total and cause-specific mortality. Circulation. 2016;133.

912. Wang T, et al. Heart-Protective Diet Scores, Cardiometabolic Risk and Cardiovascular Disease Incidence and Mortality: A Prospective Study From UK Biobank. Current Developments in Nutrition. 2023;7.

913. Ward RE, et al. Fish consumption is not associated with the risk of coronary heart disease or stroke. Circulation. 2018;137.

914. Weinreich T, et al. Risk factors and course of peripheral arterial disease (PAD) in incident chronic dialysis patients (CDP). NDT Plus. 2010;3:iii292.

915. Wilechansky RM, et al. Healthy diet patterns and risk of cardiovascular outcomes among women with nonalcoholic fatty liver disease. Hepatology. 2021;74(SUPPL 1):1013A-4A.

916. Wu H, et al. Whole grain intake and risk of all-cause and cause-specific mortality in us men and women. Circulation. 2014;129.

917. Wu H, et al. Dietary lipophilic index and lipophilic load and risk of coronary heart disease in US men and women. Circulation. 2013;127(12).

918. Xu H, et al. Fiber intake, kidney function, inflammation, and mortality in acommunity-based cohort. Nephrology Dialysis Transplantation. 2014;29:iii49.

919. Yaemsiri S, et al. Dietary fat intake and incidence of ischemic stroke in postmenopausal US women: The womens health initiative. Stroke. 2010;41(4):e223.

920. Yazdanpanah MH, et al. Dietary Intake of Minerals and Risk of Cardiovascular Mortality in the Golestan Cohort Study. Circulation. 2022;146.

921. Ye YX, et al. Planetary Healthy Diet, Environmental Impacts, and Mortality Outcomes in the Chinese Adults. Current Developments in Nutrition. 2023;7.

922. Yen T, et al. 24-hour urine potassium as a predictor of cardiac events and death in chronic kidney disease. American Journal of the Medical Sciences. 2024;367:S485.

923. Yu D, et al. High intakes of dietary carbohydrate and rice were associated with increased risk of coronary heart disease in chinese men and women. Circulation. 2013;127(12).

924. Yu D, et al. Dietary carbohydrates, glycemic index, glycemic load, and risk of stroke in chinese women: A large population-based, prospective cohort study. Stroke. 2015;46.

925. Yu D, et al. Dietary trimethylamine intake and cardiovascular mortality among urban Chinese adults. Circulation. 2017;135.

926. Zarafshar S, et al. Predictors of fatal incident coronary heart disease in the women's health initiative. Circulation. 2016;134.

927. Zeng W, et al. Lipidomics profiling and risk for cardiovascular disease: A longitudinal study in American Indians. Circulation. 2021;144(SUPPL 1).

928. Zhang Y, et al. Is fish consumption related to lower risk of mortality in china and us? A joint CHNS-NHANES study. Annals of Nutrition and Metabolism. 2017;71:754-5.

929. Zheng Y, et al. Dietary phosphatidylcholine and risk of all-cause and cardiovascular-specific mortality among women and men with type 2 diabetes. Circulation. 2015;132.

930. Zhong VW, et al. Associations of dietary cholesterol or egg consumption with incident cardiovascular disease and mortality: The lifetime risk pooling project. Circulation. 2018;138.

931. Zong G, et al. Associations between individual saturated fatty acid intake and risk of coronary heart disease among American men and women. Circulation. 2015;132.

932. Daviglus M, et al. Health benefits from eating fish. Comments on Toxicology. 2002;8(4-6):345-74.

933. de Koning L, Anand SS. Vascular viewpoint. Vascular Medicine. 2004;9(2):145-6.

934. Qiao T, et al. Using Multiple Statistical Methods to Derive Dietary Patterns Associated with Cardiovascular Disease in Patients with Type 2 Diabetes: Results from a Multiethnic Population-Based Study. Evidence-based Complementary and Alternative Medicine. 2022;2022.

935. Yang C, et al. Associations of composite dietary antioxidant index with cardiovascular disease mortality among patients with type 2 diabetes. Diabetol Metab Syndr. 2023;15(1):131.

936. Lu Q, et al. Healthy lifestyle, plasma metabolites, and risk of cardiovascular disease among individuals with diabetes. Atherosclerosis. 2023;367:48-55.

937. Ni C, et al. Association of oxidative balance score, cardiovascular, and all-cause mortality among patients with type 2 diabetes mellitus. Front Endocrinol (Lausanne). 2024;15:1429662.

938. Yuan S, et al. Dietary Inflammation Index and Its Association with Long-Term All-Cause and Cardiovascular Mortality in the General US Population by Baseline Glycemic Status. Nutrients. 2022;14(13).
